# Regulation of stress tolerance by CREB1 sustains multiple myeloma cell survival

**DOI:** 10.1038/s41419-025-08246-z

**Published:** 2026-01-16

**Authors:** Ruchi Kudalkar, Johnathan Altom, Joshua Galloway, Vincent Manning, Sara Taranto, Francesca Cottini

**Affiliations:** Department of Internal Medicine, Division of Hematology, The Ohio State University, College of Medicine, Columbus, OH USA

**Keywords:** Mechanisms of disease, Oncogenesis, Translational research, Myeloma

## Abstract

Multiple myeloma (MM) cells originate from antibody-producing plasma cells and endure chronic oxidative and proteotoxic stress due to the excessive production of immunoglobulins and free light chains. We previously demonstrated that CD56 (also known as neuronal cell adhesion molecule 1) promotes cAMP-responsive element binding (CREB1) activation in MM cells to drive survival, without fully elucidating its mechanism of action. In this study, we describe the global role of CREB1 in regulating tolerance to cellular stresses in MM. Here, we present data to demonstrate that CREB1 directly or indirectly influences key proteins involved in the clearance of oxidants, the unfolded protein response (UPR), and autophagy. In silico data from real patients with MM showed that patients with high CREB1 expression have greater activation of gene sets associated with endurance of stress. We confirmed by genomic and pharmacological modulation that CREB1 activates the mTOR pathway, halting autophagy, and directly binds to the promoter of NRF2 and PERK, modulating genes involved in oxidation and protein stress adaptation. Of particular importance was the identification of TXNIP among the regulated genes. Notably, the *TXNIP* gene belongs to the 1q21 cytoband, which is amplified in 30 percent of patients with MM, leading to poor outcomes. We showed for the first time that TXNIP inhibition is also toxic against MM cells, interfering with UPR and autophagy. Thus, our data highlights the essential roles of CREB1 and TXNIP in MM cell survival under chronic stress, providing new insights into MM pathophysiology and novel therapeutic strategies for patients with high-risk disease.

## Introduction

Reliance on oxidative stress protection serves as a defense mechanism against elevated levels of reactive oxygen species (ROS), which arise from an imbalance between ROS production and elimination [1]. Cancer cells have high levels of intracellular ROS due to increased metabolic activity, alterations in mitochondrial energetics, or hypoxic conditions, and highly depend on antioxidant defense systems [2, 3].

Multiple myeloma (MM) cells, which are a type of cancer cell originating from antibody-producing plasma cells (PCs), endure chronic oxidative stress [4] as well as proteotoxic stress due to the excessive production of immunoglobulins and free light chains [5]. To prevent the accumulation of misfolded proteins and the resulting cytotoxic protein aggregation, MM cells meticulously regulate the proteostasis network at every level. This adaptation involves reliance on the proteasome and autophagosome systems [6], constitutive activation of the mammalian target of rapamycin (mTOR) pathway [7], greater expression of small heat shock chaperone proteins such as HSP60, HSP70, and HSP90 compared to normal PCs [8], and activation of the unfolded protein response (UPR) [9]. The use of proteasome inhibitors, like bortezomib and carfilzomib, has greatly improved the outcomes for patients with MM [6]. Targeting mTOR or chaperone proteins has also been studied in pre-clinical models or clinical trials [10, 11]. However, most patients develop resistance to these therapeutic strategies, resulting in disease relapse. Among the various mechanisms linked to proteasome inhibitor resistance, the induction of POMP and NFE2L2 (NFE2-like bZIP transcription factor 2, also called NRF2) not only enhances tolerance to ROS but also increases proteasome capacity by alleviating the imbalance between protein load and capacity [12].

We recently demonstrated that MM cells are characterized by the activation of the transcription factor CREB1 (cAMP-responsive element binding protein 1) mediated by CD56 (NCAM1 or neural cell adhesion molecule) [13]. However, the mechanistic contribution of CREB1 to MM survival remains largely unexplored. Interestingly, CREB1 is essential for regulating autophagy and oxidative sensing in both neurons and mesenchymal stem cells, primarily through signaling pathways involving brain-derived neurotrophic factor (BDNF) and NRF2 [14–16]. Therefore, we hypothesize that CREB1 could also be implicated in the regulation of oxidative stress and proteostasis in MM cells.

Here, we demonstrate that CREB1 is vital for promoting tolerance to oxidative stress caused by ROS and proteotoxic stress. CREB1 expression activates the mTOR pathway, enhances the adaptive UPR through PERK (Eukaryotic translation initiation factor 2-alpha kinase 3, EIF2AK3), and regulates genes that control oxidant levels, such as *NRF2* and *BACH1* (BTB and CNC homology 1). In contrast, inhibiting CREB1 results in increased ROS levels, triggering autophagy and apoptosis. Additionally, we discovered that *TXNIP* (Thioredoxin-interacting protein), a redox gene overexpressed in MM cells and amplified in the 1q cytoband, is also indirectly regulated by CREB1 through PERK. Silencing or inhibiting TXNIP proves detrimental to MM cells, disrupting the UPR response and impairing ROS clearance. Pharmacological inhibition of either CREB1 or TXNIP synergizes with proteasome inhibitors, offering a novel therapeutic strategy for patients with MM.

## Methods

### Cell lines and cultures

The human cell lines H929, RPMI-8226, MM.1S, MM.1R, and HEK293T cells were purchased from the American Type Culture Collection (ATCC), while the human cell lines U266, JJN-3, and OPM-2 were purchased from the Leibniz Institute DSMZ-German Collection of Microorganisms and Cell Cultures (DSMZ). KMS-11 and KMS-11 BTZ were provided by Dr. Arianna Giacomini, University of Brescia. All MM cell lines were cultured in RPMI-1640 (Gibco, Thermo Fisher Scientific, Cat. No. 11875093, Waltham, MA, USA) medium containing 10% Fetal Bovine Serum (FBS, Gibco, Thermo Fisher Scientific, Cat. No. 16140071), 2 µM/L glutamine, 10,000 U/mL penicillin G, and 10,000 μg/mL streptomycin (Gibco, Thermo Fisher Scientific), and maintained at 37 °C with 5% CO_2_. HEK293T cells were cultured in Dulbecco’s Modified Eagle Medium (DMEM) (Gibco, Thermo Fisher Scientific, Cat. No. 12430054), containing 10% FBS, 2 µM/L glutamine, 10,000 U/mL penicillin G, and 10,000 μg/mL streptomycin, and maintained at 37 °C with 5% CO_2_. Cells were used within 2–3 months after thawing (24–40 passages at maximum). Cell lines were also regularly evaluated for mycoplasma contamination using the MycoAlert mycoplasma detection kit (Lonza, Cat. No. LT07-418, Basel, Switzerland).

### RNA extraction and quantitative real-time PCR analysis

RNA for quantitative real-time PCR was extracted using the TRIzol method (Invitrogen, Life Technologies, Cat. No. 15596026, Carlsbad, CA, USA); cDNA was synthesized using the ProtoScript® II First Strand cDNA Synthesis Kit (New England Biolabs, Ipswich, MA, USA). Quantitative real-time PCR analysis was performed using the SYBR GREEN PCR Master Mix protocol (Applied Biosystems, CA, USA, Cat. No. 4309155). Quantitative real-time PCR analysis was performed on a ViiA 7 Real-Time PCR System (Applied Biosystems, CA, USA). Data were analyzed using the ∆∆Ct method. Gene-specific primers are reported in the *Supplementary Methods*. GAPDH was used for normalization.

### Western blot analysis

MM cells were harvested and lysed using RIPA lysis buffer (Cell signaling, Cat. No. 9806), with the addition of 1 mM PMSF (Cell signaling, Cat. No. 8553). Laemmli Sample Buffer (BIO-RAD, Cat. No. 1610747) was added, and samples were boiled at 95 °C. Cell lysates were subjected to SDS–PAGE, transferred to nitrocellulose membranes, and immunoblotted with the specific antibodies reported in the *Supplementary Methods*. All antibodies were diluted to a concentration of 1:1000 and prepared in milk, 5% diluted in TBS (BIO-RAD, Cat. No. 1706435) with Tween 20 (BIO-RAD, Cat. No. 1706531). Optical densitometry analysis of specific bands was performed using ImageJ software (ImageJ, RRID:SCR_003070).

### CREB1 and TXNIP silencing and overexpression in MM cell lines

For overexpression experiments, U266 cells were transiently transfected using Nucleofector 4D Unit X, Kit SF, program DY-100 (Lonza, Amaxa Biosystems, Köln, Germany). For silencing experiments, viruses were produced by co-transfection of specific plasmids and packaging vectors (psPAX2, Addgene Cat. No. 12260, RRID:Addgene_12260 and pMD2.G, Addgene Cat. No. 12259, RRID:Addgene_12259) into HEK293T cells. H929 and OPM-2 cells were infected with lentiviral particles using polybrene followed by spinoculation of suspended cells. Stably infected cells were selected using 0.5–1 μg/mL of puromycin (InvivoGen, Cat. No. ant-pr-1). After transfection or infection, MM cells were subjected to mRNA analysis, western blotting, and flow cytometry analysis.

### Specific assays for autophagy, reactive oxygen species quantification, nascent protein synthesis, and apoptosis by flow cytometry

Autophagy, redox status, mitosox activity, nascent protein synthesis, and Annexin V-PI were quantified using specific kits as described in the *Supplementary Methods*. All flow cytometry experiments were acquired on the Attune NxT Flow cytometry machine using Attune NxT Software v 3.1 (Thermo Fisher Scientific). At least 10,000 events were acquired. Post-acquisition analyses were performed using FlowJo Software (BD Biosciences).

### RNA-sequencing, copy number variation, and gene-expression profiling analysis

RNA-sequencing (gene and transcript-based) data were downloaded from the MMRF CoMMpass database, with data generated as part of the Multiple Myeloma Research Foundation Personalized Medicine Initiatives (https://research.themmrf.org and www.themmrf.org). mRNA expression data were obtained from GSE4452, a collection of 12 healthy donors and 65 newly diagnosed MM patients, and analyzed by HG-U133 Plus 2.0 array. RNA-sequencing data for normal immune populations were collected from the GSE107011 dataset as described in Monaco et al. [17]. Median value of expression of CREB1 or TXNIP was used as a threshold to define patients with high or low expression of these two genes in the CoMMpass and GSE4452 datasets. Gene sets of canonical pathways (C2_CP_BIOCARTA, C2_CP_KEGG, C2_CP_PID, C2_CP_REACTOME, C2_CP_WIKIPATHWAYS) or gene ontology (C5_GOBP) were used to perform enrichment analysis at http://www.broad.mit.edu/gsea. Student’s *t*-test and linear regression analysis were performed to correlate CREB1 levels with the expression levels of specific genes, including TXNIP, NRF2, BACH1, PERK, EI2FA, IRE1, XBP1, and ATF6 in the CoMMpass and GSE4452 datasets. RNA-sequencing data were also used to correlate the expression of TXNIP to the presence or absence of t(4;14). CREB1 transcript analysis was used to evaluate the various CREB1 isoforms. Whole *TXNIP* amplification data were obtained from the MMRF CoMMpass database, and exponential coefficient copy number variation analysis was performed. Two-sided *p*-values < 0.05 were considered statistically significant. Statistical analyses were performed using GraphPad software (GraphPad Prism, RRID:SCR_002798).

### Chromatin immunoprecipitation (ChIP) protocol and ChIP-sequencing

H929 cells were treated with DMSO or 666-15 (CREB1 inhibitor) at 2 μM for 24 h. Protein–DNA complex chromatin extraction was performed using Magna ChIP™ A/G kit (Millipore, Burlington, MA, USA, Cat. No. 17-10085), starting from five million cells per condition. The following CREB1 antibody was used: CREB1 (Cell Signaling Technology, Cat. No. 9197, RRID:AB_331277). ChIP-Sequencing and post-processing of the raw data were performed by Active Motif, Inc. (Carlsbad, CA, USA), and data are included as Supplementary Table S1. Images were obtained using Integrative Genomics Viewer software version 2.16 (Broad Institute).

## Results

### CREB1 regulates the response to oxidative stress in MM cells

**Fig. 1:**
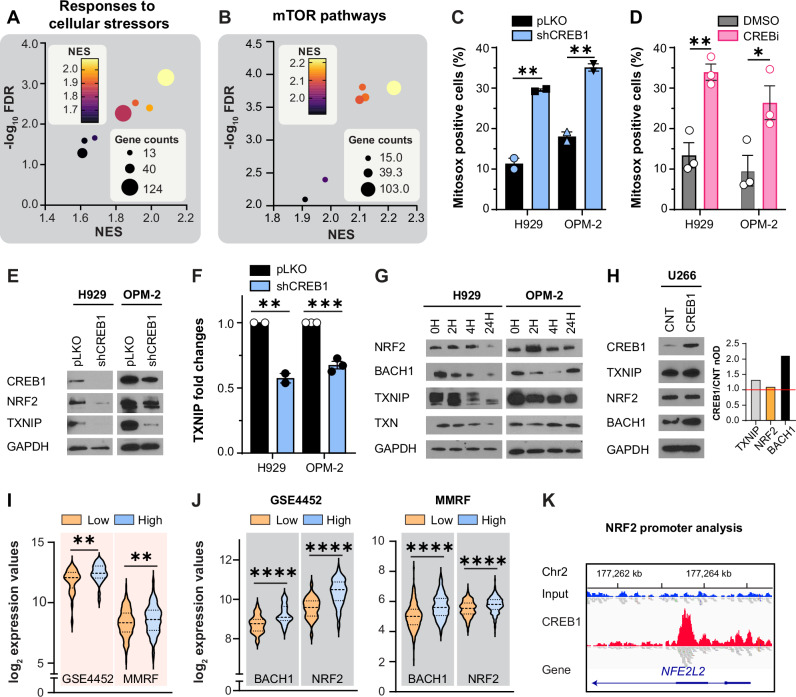
CREB1 regulates tolerance to ROS-induced oxidative stress. **A** Pathway analysis of the upregulated gene sets related to responses to cellular stressors in the MMRF CoMMpass database. *n* = 770 patients; patients are divided based on median CREB1 expression. NES, normalized enrichment score; FDR, false discovery rate; gene counts include the number of significant genes in the pathway. **B** Pathway analysis of the upregulated mTOR gene sets in the MMRF CoMMpass database. Patients are divided based on median CREB1 expression as in (**A**). **C** Percentage of Mitosox-positive cells (marker of mitochondrial ROS) in H929 and OPM-2 control cells (pLKO), or cells silenced for CREB1 (shCREB1). Error bars represent the mean ± SEM, *n* = 2 experimental replicates. H929 *p* = 0.0058, **; OPM-2 *p* = 0.0073, **; Student’s *t*-test. **D** Percentage of Mitosox-positive cells in H929 and OPM-2 cells treated with DMSO or 666-15 (CREBi) 0.3 μM for 48 h. Error bars represent the mean ± SEM, *n* = 3 experimental replicates. H929 *p* = 0.005, **; OPM-2 *p* = 0.04, *; Student’s *t*-test. **E** Western blot analysis for CREB1, NRF2, TXNIP, and GAPDH in H929 and OPM-2 control cells (pLKO), or cells silenced for CREB1 (shCREB1). **F** TXNIP mRNA fold changes in H929 and OPM-2 control cells (pLKO), or cells silenced for CREB1 (shCREB1). Error bars represent the mean ± SEM. H929: *n* = 2 experimental replicates *p* = 0.0073, **. OPM-2: *n* = 3 experimental replicates, *p* = 0.0004, ***; Student’s *t*-test. **G** Western blot analysis for NRF2, BACH1, TXNIP, TXN, and GAPDH in H929 and OPM-2 cells treated with DMSO or CREBi 1 μM for 0, 2, 4, and 24 h. **H** Western blot analysis for CREB1, NRF2, BACH1, TXNIP, and GAPDH in U266 control cells (CNT) or U266 cells overexpressing CREB1. Quantitative densitometry analysis is presented as the ratio of the proteins of interest (TXNIP, NRF2, and BACH1) to GAPDH. The values obtained for CREB1 overexpressing cells are normalized to those of the control cells. nOD, normalized optical density. **I** TXNIP log_2_ expression values based on median CREB1 expression in the GSE4452 and MMRF CoMMpass databases. Black dashed lines represent the median value; black dotted lines represent the 25^th^ and 75^th^ percentiles. GSE4452 database: *n* = 65 patients, *p* = 0.0045, **. MMRF CoMMpass database: *n* = 770 patients, *p* = 0.0024; **; Student’s *t*-test. **J** BACH1 and NRF2 log_2_ expression values based on median CREB1 expression in the GSE4452 and MMRF CoMMpass database. Black dashed lines represent the median value; black dotted lines represent the 25^th^ and 75^th^ percentiles. GSE4452 database: *n* = 65 patients, BACH1 *p* < 0.0001, ****; NRF2 *p* < 0.0001, ****. MMRF CoMMpass database: *n* = 770 patients, BACH1 *p* < 0.0001, ****; NRF2 *p* < 0.0001, ****; Student’s *t*-test. **K** ChIP-seq tracks for CREB1 binding to *NFE2L2* (*NRF2*) promoter.

We initially analyzed the most enriched pathways in patients with MM grouped by median CREB1 expression, noting that patients with high CREB1 expression had greater activation of gene sets associated with endurance of oxidative stress, modulation of UPR, and induction of mTOR signaling (Fig. 1A, B and Supplementary Fig. S1A, B). Given the role of CREB1 in regulating oxidative stress in neurons and mesenchymal stem cells [14, 16], we hypothesize that CREB1 could help maintain low levels of ROS and avoid proteotoxic stress in MM. We then investigated whether silencing CREB1 or inhibiting it with 666-15 compound (called in the manuscript CREBi [18]) could induce ROS production. We observed an increase in total and mitochondrial ROS at basal conditions (Fig. 1C, D and Supplementary Fig. S2A) and in the presence of terminal oxidants such as t-Butyl hydroperoxide, TBHP (Supplementary Fig. S2B), or proteasome inhibitors bortezomib and carfilzomib (Supplementary Fig. S2C, D).

NRF2, BACH1, and TXNIP regulate anti-oxidant genes [19] and are essential for B cell development [3, 20–22]. Since CREB1 inhibition led to an increase in ROS, we hypothesize that CREB1 could affect the levels of these genes. Importantly, shRNAs against CREB1 downregulated the protein and mRNA levels of NRF2, BACH1, and TXNIP (Fig. 1E, F and Supplementary Fig. S3A) and increased the levels of FBXO22 (Supplementary Fig. S3B), a protein that promotes BACH1 degradation. Pharmacological inhibition of CREB1 using 666-15 decreased NRF2 and TXNIP levels, had minimal effects on BACH1 levels (Fig. 1G and Supplementary Fig. S3C, D), and increased FBXO22 levels (Supplementary Fig. S3E, F). In contrast, the overexpression of CREB1 had opposite effects, increasing the protein levels of TXNIP, NRF2, and BACH1 (Fig. 1H). This also correlated with greater mRNA levels of TXNIP, BACH1, and NRF2 in patients with high CREB1 expression, as observed in two independent datasets (Fig. 1I, J).

CREB1 can both activate and repress gene transcription due to multiple isoforms generated by alternative splicing [23, 24]. In patients with MM, the predominant isoforms are full-length activators A (ENST00000353267, ENST00000430624) and B (ENST00000432329), though shorter, potentially inhibitory isoforms are also expressed (Supplementary Fig. S3G). CHIP-sequencing analysis in MM cells (Supplementary Table S1) revealed that CREB1 directly binds to the promoters of *NRF2* (Fig. 1K) and *FBXO22* (Supplementary Fig. S3H), but not to those of *BACH1* or *TXNIP* (Supplementary Fig. S3I, J). These findings indicate that CREB1 modulates the transcription of genes involved in the clearance of oxidants, either promoting or repressing their expression.

### CREB1 activates mTOR signaling, promoting protein synthesis, and preventing autophagy in MM cells

**Fig. 2:**
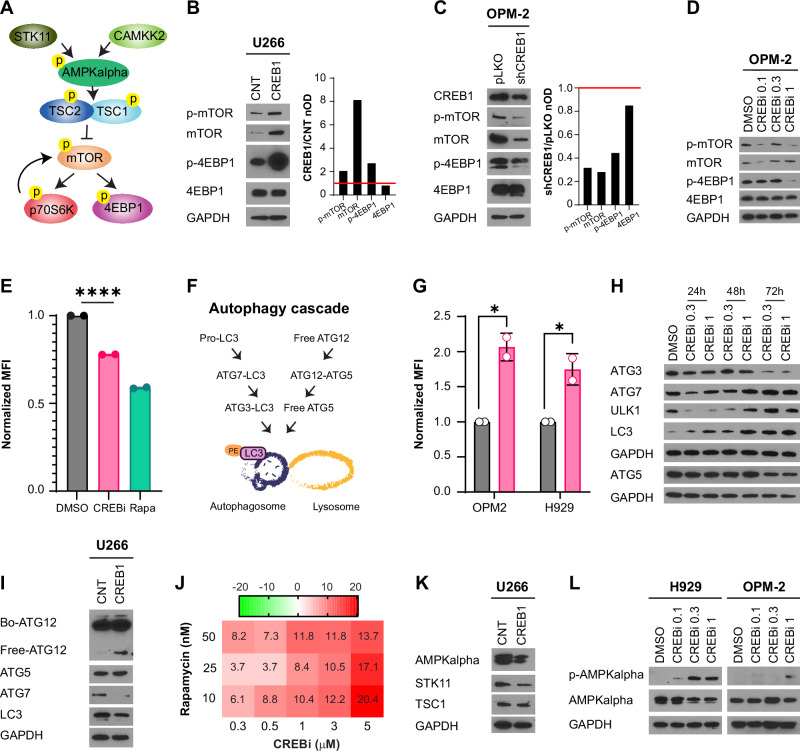
CREB1 modulates mTOR pathway and autophagy. **A** Schema of mTOR activation. The two most prominent and well-characterized translational regulators activated by the mTOR kinase, downstream of mTORC1, are eIF4E binding proteins (4EBP1) and ribosomal protein S6 kinase beta-1 (p70S6K). 4EBP1 inhibits the initiation of translation unless it is phosphorylated by mTORC1, while p70S6K participates in the control of mRNA translation. TSC1/TSC2 (TSC complex subunit 1 and 2) negatively regulates mTOR when phosphorylated by AMPKalpha (protein kinase AMP-activated catalytic subunit alpha 1), which in turn is activated by either CAMKK2 (calcium/calmodulin-dependent protein kinase 2) or STK11 (serine/threonine kinase 11). **B** Western blot analysis for total and phospho-mTOR (Ser2448), total and phospho-4EBP1, and GAPDH in U266 control cells (CNT) or U266 cells overexpressing CREB1. Quantitative densitometry analysis is presented as the ratio of the proteins of interest to GAPDH. The values obtained for CREB1 overexpressing cells are normalized to those of the control cells. nOD, normalized optical density. **C** Western blot analysis for total and phospho-mTOR (Ser2448), total and phospho-4EBP1, and GAPDH in OPM-2 control cells (pLKO) or cells silenced for CREB1 (shCREB1). Quantitative densitometry analysis is presented as the ratio of the proteins of interest to GAPDH. The values obtained for cells silenced for CREB1 are normalized to those of the control cells. **D** Western blot analysis for total and phospho-mTOR (Ser 2448), total and phospho-4EBP1, and GAPDH in OPM-2 cells treated with DMSO or 666-15 (CREBi) 0.1, 0.3, and 1 μM for 48 h. **E** Nascent protein labeling by Click-IT assay in OPM-2 cells treated with DMSO, CREBi 5 μM, or rapamycin 50 nM for 24 h. Mean fluorescence intensity (MFI) normalized to DMSO is shown. Error bars represent the mean ± SEM, *n* = 2 experimental replicates. DMSO versus CREBi *p* < 0.0001, ****; Student’s *t*-test. **F** Schema of autophagy. A cascade of activation of ATG3, ATG5, ATG12, and ATG7 leads to the phosphatidylethanolamine (PE)-conjugation of LC3 and its translocation to the autophagosome membrane, which leads to the fusion of the autophagosome to lysosomes for degradation purposes. **G** MFI fold change of autophagy marker CytoID in H929 and OPM-2 cells treated with DMSO or CREBi 1 μM for 48 h. MFI ratio is normalized to the DMSO value. Error bars represent the mean ± SEM, *n* = 2 experimental replicates. H929 *p* = 0.016, *; OPM-2 *p* = 0.0423, *; Student’s *t*-test. **H** Western blot analysis for ATG3, ATG5, ATG7, LC3, ULK1, and GAPDH in H929 cells treated with DMSO or CREBi 0.3 and 1 μM for 24, 48, or 72 h. **I** Western blot analysis for ATG7, LC3, ATG5, ATG12 (the antibody detects both bound ATG5/ATG12 and unbound ATG12), and GAPDH in U266 CNT cells or U266 cells overexpressing CREB1**. J** Synergy scores in OPM-2 cells treated with DMSO, CREBi 0–5 μM, rapamycin (10–50 nM), and all their combinations. Values represent the synergy scores calculated using the average of two experimental replicates. **K** Western blot analysis for AMPKalpha, STK11, TSC1, and GAPDH in U266 CNT cells or U266 cells overexpressing CREB1. **L** Western blot analysis for phospho-AMPKalpha (Thr172), total AMPKalpha, and GAPDH in H929 and OPM-2 cells treated with DMSO, CREBi 0.1, 0.3, or 1 μM for 48 h.

mTOR gene sets pathways were also among the most upregulated pathways in patients with high CREB1 expression (Fig. 1B). mTOR signaling promotes protein synthesis and prevents autophagy, a process otherwise used by the cells to destroy protein aggregates or induce ROS-mediated cell death [25, 26]. Therefore, we hypothesized that CREB1 could activate mTOR pathways for two purposes: to initiate protein translation and to avoid autophagy-mediated cell death. We first confirmed in U266 cells that the overexpression of CREB1 induced phosphorylation of mTOR itself and its downstream targets, EIF4EBP1 (eukaryotic translation initiation factor 4E-binding protein 1, referred to as 4EBP1 in the manuscript) and RPS6KB1 (ribosomal protein S6 kinase B1, referred to as p70S6K in the manuscript), all reliable markers of mTOR signaling activation (Fig. 2A, B and Supplementary Fig. S4A). In contrast, both CREB1 silencing and treatment with CREBi reduced phosphorylation of mTOR and 4EBP1 (Fig. 2C, D, and Supplementary Fig. S4B), with fewer effects on phospho-P70S6K (Supplementary Fig. S4C). We then utilized an assay to quantify nascent protein synthesis, using rapamycin as a positive control, due to its ability to reduce global protein synthesis by blocking mTOR activity [27]. CREB1 inhibition similarly led to reduced protein synthesis (Fig. 2E). Together, these findings suggest that CREB1 activation promotes mTOR signaling and regulates protein synthesis.

Active mTOR suppresses autophagy, a process crucial for removing protein aggregates or damaged mitochondria via autophagosome-lysosomes fusion (Fig. 2F). Although autophagy supports cell survival during nutrient deprivation, it can also trigger ROS-mediated cell death under stress conditions [28]. As expected, CREB1 inhibition promoted autophagy, as shown by CytoID staining (Fig. 2G), with induction of ULK1 and LC3, and time-dependent changes of ATG3, ATG5, and ATG7 (Fig. 2H and Supplementary Fig. S4D–F). In contrast, CREB1 overexpression reduced ATG7 and LC3 levels while increasing free, unbound ATG5 and ATG12 (Fig. 2I and Supplementary Fig. S4G), thereby limiting the process of degradation. Finally, CREB1 inhibition in combination with rapamycin synergistically induced cell death (Fig. 2J), due to the complete abrogation of mTOR signaling (Supplementary Fig. S4H).

To investigate how mTOR was activated, we evaluated the expression and phosphorylation of upstream inhibitory regulators, including TSC1 (TSC complex subunit 1), AMPKalpha (AMP-activated protein kinase alpha subunit), CAMKK2 (calcium/calmodulin-dependent protein kinase kinase 2), and STK11 (serine/threonine kinase 11) (Fig. 2A). CREB1 overexpression reduced total levels of AMPKalpha, TSC1, and STK11 (Fig. 2K), while phospho-AMPKalpha was undetectable in both control and CREB1-overexpressing cells. In turn, CREB1 inhibition increased the phosphorylation of AMPKalpha and CAMKK2, along with elevated total protein and mRNA levels of AMPKalpha and TSC1 (Fig. 2L and Supplementary Fig. S4I, J), leading to mTOR suppression and autophagy induction. These data suggest that CREB1 activates the mTOR pathway by inhibiting AMPKalpha, thereby promoting cell survival and halting autophagy in MM cells.

### CREB1 modulates the adaptive UPR, allowing tolerance of proteotoxic stress

**Fig. 3:**
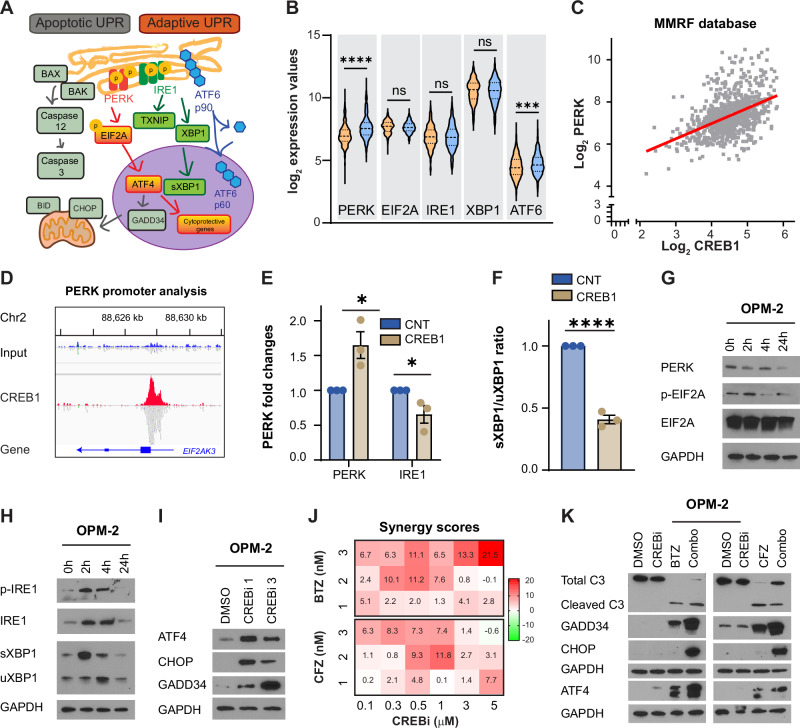
CREB1 controls the unfolded protein response in MM. **A** Schema of the UPR signaling pathway. The UPR activates pro-apoptotic pathways when ER stress is prolonged, leading to the activation of BAX, BAK, and caspases 12 and 3, which promote apoptosis. CHOP, induced by ATF4, also promotes apoptosis by inhibiting BCL2 and triggering mitochondrial dysfunction via BID. Concurrently, to cope with ER stress, the UPR also initiates adaptive responses through the PERK, ATF6, and IRE1 pathways, leading to a general reduction in protein translation and enhanced expression of genes involved in protein folding and ER-associated degradation. **B** PERK, EI2FA, IRE1, XBP1, and ATF6 log_2_ expression values based on median CREB1 expression in the MMRF CoMMpass database. Black dashed lines represent the median value, black dotted lines represent the 25^th^ and 75^th^ percentiles, *n* = 770 patients. PERK *p* < 0.0001, ****; ATF6 *p* = 0.0002, ***. All other comparisons are not significant, ns, Student’s *t*-test. **C** Correlation between CREB1 and EIF2AK3 (PERK) levels in the MMRF CoMMpass database. *p* < 0.0001, *R* = 0.21; simple linear regression. **D** ChIP-seq tracks for CREB1 binding to *PERK* promoter. **E** PERK and IRE1 mRNA fold changes in U266 control cells (CNT) or U266 cells overexpressing CREB1. Error bars represent the mean ± SEM, *n* = 3 experimental replicates. PERK *p* = 0.0275, *; IRE1 *p* = 0.05, *; Student’s *t*-test. **F**. sXBP1/uXBP1 ratio in U266 CNT cells or U266 cells overexpressing CREB1. Error bars represent the mean ± SEM, *n* = 3 experimental replicates. *p* < 0.0001, ****; Student’s *t*-test. **G** Western blot analysis for PERK, phospho-EIF2A, EIF2A, and GAPDH in OPM-2 cells treated with DMSO or 666-15 (CREBi) 1 μM for 2, 4 and 24 h. **H** Western blot analysis for phospho-IRE1, IRE1, XBP1 (higher molecular weight band is sXBP1 while lower molecular weight band is uXBP1), and GAPDH in OPM-2 cells treated with DMSO or CREBi 1 μM for 2, 4, and 24 h. **I** Western blot analysis for ATF4, CHOP, GADD34, and GAPDH in OPM-2 cells treated with DMSO or CREBi 1 and 3 μM for 48 h. **J** OPM-2 cells treated with DMSO, CREBi 0–5 μM, bortezomib (BTZ) 1, 2, 3 nM, carfilzomib (CFZ) 1, 2, 3 nM, and all their combinations. Values represent the synergy scores calculated using the mean of two experimental replicates. **K** Western blot analysis for caspase 3 (the upper band is the full-length form, total C3, while the lower band is the cleaved form, cleaved C3), GADD34, CHOP, ATF4, and GAPDH in OPM-2 cells treated with DMSO, CREBi 0.3 μM, BTZ 5 nM, or CREBi + BTZ (combo) for 24 h (left panel) or with DMSO, CREBi 0.3 μM, CFZ 5 nM, or CREBi + CFZ (combo) for 24 h (right panel).

By limiting autophagy, the mTOR pathway further increases the burden of proteasome load to MM cells [11]. Therefore, we reasoned that MM cells would rely even more on the UPR as an adaptive response to prevent the accumulation of misfolded proteins and avoid terminal oxidative stress (Fig. 3A). Indeed, in two independent datasets, patients with high CREB1 expression had greater levels of EIF2AK3 (eukaryotic translation initiation factor 2 alpha kinase 3, also known as PERK) and ATF6 (activating transcription factor 6) (Fig. 3B and Supplementary Fig S5A), two proteins involved in the survival of MM cells in the presence of misfolded proteins. We also observed a robust correlation between the expression of CREB1 and PERK by linear regression analysis (*R* = 0.21 or *R* = 0.25, *p* < 0.0001) in the same two datasets (Fig. 3C and Supplementary Fig. S5B), but no correlation with other UPR genes including ATF6 (*R* = 0.04; *p* < 0.0001), EIF2A (eukaryotic translation initiation factor 2A; *R* = 0.008; *p* = 0.0092), ERN1 (endoplasmic reticulum to nucleus signaling 1, also known as IRE1; *R* = 0.0007; *p* not significant), and XBP1 (X-box binding protein 1; *R* = 0.00; *p* not significant). CHIP-sequencing analysis demonstrated that CREB1 binds to *PERK* promoter (Fig. 3D), which was validated by overexpression of CREB1 in U266 cells (Fig. 3E and Supplementary Fig. S5C). While PERK was upregulated, IRE1 was less phosphorylated and downregulated at mRNA levels by CREB1 expression (Fig. 3E and Supplementary Fig. S5C), resulting in an increase of total XBP1, but a reduction of the spliced XBP1/unspliced XBP1 (sXBP1/uXBP1) ratio by quantitative PCR (Fig. 3F), as a protective mechanism. Based on these data, we believe that PERK activation by CREB1 counterbalances the drive for protein synthesis induced by mTOR and promotes the expression of genes for stress adaptation, including TXNIP. At the same time, CREB1 inhibits IRE1, preventing the initiation of the cell death pathway.

We then blocked CREB1 expression and function by genomic inhibition or pharmacological inhibition with the 666-15 compound, expecting a shift towards cellular death mechanisms. Using shRNAs, we observed decreased mRNA and protein expression of PERK, increased protein expression of IRE1, and a reduction of total XBP1 mRNA levels with changes in XBP1 splicing (Supplementary Fig. S5D and E). With the CREB1 inhibitor 666-15, we observed PERK downregulation (Fig. 3G), while phospho-EIF2A increased at 2 h and then normalized (Fig. 3G). We also confirmed an increase in both total and phosphorylated IRE1 after 2- and 4-h treatments, which returned to baseline levels by 24 h. Similarly, the spliced form of XBP1 increased initially, followed by a reduction in both spliced and unspliced XBP1 at 24 h (Fig. 3H and Supplementary Fig. S5F). IRE1 mRNA levels also initially increased at 2 h to then normalize (Supplementary Fig. S5G), while the sXBP1/uXBP1 ratio initially increased to then decrease (Supplementary Fig. S5H), following a similar but anteceding pattern compared to the corresponding proteins. All these findings, including the rapid and transient phosphorylation of EIF2A, point toward activation of ER stress-mediated cell death [29]. Indeed, after prolonged treatment with CREBi, ATF4 (activating transcription factor 4) was induced, promoting the expression of apoptotic UPR markers DDIT3 (DNA damage inducible transcript 3, referred to as CHOP in the manuscript) and PPP1R15A (protein phosphatase 1 regulatory subunit 15A, referred to as GADD34 in the manuscript) (Fig. 3I and Supplementary Fig. S5I).

Proteasome inhibitors heighten endoplasmic reticulum (ER) stress by preventing the degradation of substrates and reducing stress signaling via PERK and XBP1 inhibition [30], ultimately inducing apoptotic UPR. We previously reported that CREB1 inhibition is cytotoxic towards MM cells [13, 31]. Building on this, we hypothesized that dual inhibition of CREB1 and the proteasome could produce a synergistic effect given the shared influence on UPR pathways. Indeed, we demonstrated that CREBi inhibition, when combined with low doses of bortezomib or carfilzomib, results in a synergistic anti-MM effect (Fig. 3J and Supplementary Fig. S6A and S6B). Apoptotic UPR markers, such as cleaved CASP3 (caspase 3), GADD34, CHOP, and ATF4, were also increased by CREBi as a single agent or in combination with proteasome inhibitors (Fig. 3K and Supplementary Fig. S6C–F). Interestingly, the CREBi inhibitor at 0.3 and 1 μM also enhanced the activity of proteasomes, an additional mechanism associated with the additive effects (Supplementary Fig. S6G). Overall, our data indicate that CREB1 modulates the balance between adaptive versus apoptotic UPR and its inhibition potentiates the activity of proteasome inhibitors.

### TXNIP is overexpressed in multiple myeloma and regulates cellular growth and response to stressors

**Fig. 4:**
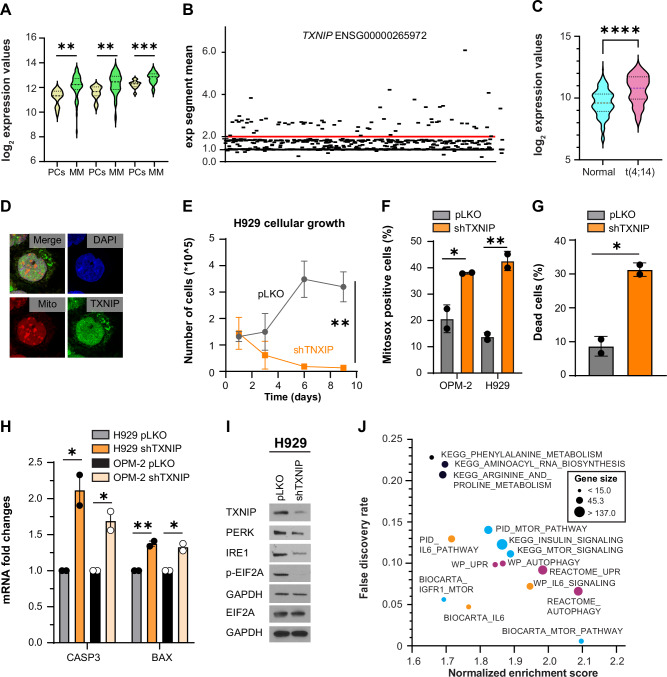
TXNIP is overexpressed in multiple myeloma where it regulates cellular growth and response to stressors. **A** TXNIP expression in *n* = 12 normal plasma cells from healthy donors (PCs) or 65 patients with newly diagnosed MM. From left to right probes: 201008_s_at (*p* = 0.0013, **), 201009_s_at (*p* = 0.0054, **), and 201010_s_at (*p* = 0.0005, ***). Data from GSE4452 database, Student’s *t*-test. **B** Exponential coefficients of copy number values for *TXNIP* (ENSG00000265972) on chromosome 1 in 858 newly diagnosed patients in the MMRF CoMMpass database. **C** TXNIP RNA-sequencing expression values based on presence or absence of t(4;14) from the MMRF CoMMpass database. *p* < 0.0001, ****; Student’s *t*-test. **D** Confocal imaging of OPM-2 cells stained for TXNIP, Mitosox (Mito), DAPI, and merged image (merge). ×63 images. **E** Cellular growth in H929 control cells (pLKO) or cells silenced for TXNIP (shTXNIP). Error bars represent the mean ± SD, *n* = 3 experimental replicates. *p* = 0.0016, **; Student’s *t*-test. **F** Percentage of Mitosox-positive cells in OPM-2 and H929 control cells (pLKO) or cells silenced for TXNIP (shTXNIP). Error bars represent the mean ± SEM. OPM-2: *n* = 2 experimental replicates; *p* = 0.043, *. H929: *n* = 2 experimental replicates, *p* = 0.009, **; Student’s *t*-test. **G** Percentage of dead cells (Annexin V^+^, PI^+^) in H929 control cells (pLKO) or cells silenced for TXNIP (shTXNIP). Error bars represent the mean ± SEM, *n* = 2 experimental replicates. *p* = 0.012, *; Student’s *t*-test. **H** Caspase 3 (CASP3) and BAX mRNA fold changes in H929 and OPM-2 control cells (pLKO) or cells silenced for TXNIP (shTXNIP). Error bars represent the mean ± SEM, *n* = 2 experimental replicates. CASP3: H929 *p* = 0.03, *; OPM-2 *p* = 0.033, *; BAX: H929 *p* = 0.009, **; OPM-2 *p* = 0.026, *; Student’s *t*-test. **I** Western blot analysis for TXNIP, PERK, IRE1, phospho-EIF2A, total EIF2A, and GAPDH in H929 control cells (pLKO) or cells silenced for TXNIP (shTXNIP). **J** Pathway analysis of the upregulated gene sets in the MMRF CoMMpass database. *n* = 770 patients; patients are divided based on median TXNIP expression. Gene counts include the number of significant genes in the pathway.

TXNIP, a protein involved in both oxidative stress regulation and UPR, is induced by PERK [32] and, indirectly, by CREB1. We therefore propose that TXNIP may represent a novel and clinically relevant factor in myeloma. We first evaluated the mRNA and protein expression of TXNIP in different subsets of B cells or patients with MM. TXNIP expression was higher in naïve B cells but lower in normal plasmablasts (Supplementary Fig. S7A, B, and [33]). However, its expression was then markedly upregulated in MM cells from patients compared with normal PCs (Fig. 4A) and was present in most of the MM cell lines (Supplementary Fig. S7C). The *TXNIP* gene is located within the 1q cytoband. We confirmed in the CoMMpass MMRF dataset that the *TXNIP* gene is amplified in 30% of patients (Fig. 4B). Furthermore, TXNIP is also elevated in patients with translocation t(4;14) (Fig. 4C). Therefore, TXNIP expression is higher in patients classified as having high-risk cytogenetic features according to the criteria established by the International Myeloma Working Group 2025 guidelines [34].

Under normal physiological conditions, TXNIP is localized in the nucleus. However, in the presence of ROS, TXNIP translocates to the cytoplasm, where it interacts with thioredoxin (TXN) to regulate stress in the ER and mitochondria. Our findings demonstrate that TXNIP is constitutively present in both the nucleus and cytoplasm (Fig. 4D), and it binds to TXN (Supplementary Fig. S7D), as previously described [35]. Additionally, TXNIP levels increase in response to the ER stress inducer tunicamycin (Supplementary Fig. S7E), indicating that TXNIP plays a role in managing ongoing stress responses in MM cells.

All this evidence suggests that MM cells reactivate TXNIP expression as a survival mechanism. To confirm this hypothesis, we silenced TXNIP (Supplementary Fig. S8A) by lentiviral infection and observed a reduction in cellular growth (Fig. 4E and Supplementary Fig. S8B) and viability (Supplementary Fig. S8C). Similarly, TXNIP silencing increased the levels of ROS (Fig. 4F) and induced apoptosis (Fig. 4G and Supplementary Fig. S8D) by activation of CASP3 and BAX (Fig. 4H). To investigate the pathways modulated by TXNIP, we performed RNA-sequencing experiments and analyzed data from the CoMMpass MMRF database. H929 cells silenced for TXNIP showed activation of oxidation, TNF-alpha and INF-gamma signaling, and apoptotic pathways (Supplementary Fig. S8E), upregulation of UPR and ER stress apoptotic markers ATF3 (activating transcription factor 3), CASP1 (caspase 1), HSPA5 (heat shock protein family A (Hsp70) member 5), CHOP, and ERO1B (endoplasmic reticulum oxidoreductase 1 beta), and downregulation of IRE1 (Supplementary Fig. S8F), a phenotype similar to CREB1 inhibition. Changes in UPR proteins were confirmed by western blot and quantitative PCR. Specifically, cells silenced for TXNIP had a reduction of markers of adaptive UPR, such as PERK, IRE1, and phospho-EIF2A (Fig. 4I and Supplementary Fig. S8G), but an increase of CHOP (Supplementary Fig. S8H). We then focused on RNA-sequencing data from patients divided based on TXNIP median expression, to identify driving survival pathways associated with TXNIP. Notably, patients with high TXNIP expression had activation of pathways involved in proteostasis (mTOR and UPR), MM survival (IL6, insulin, and NOTCH1 signaling), and energy control (amino acid metabolism and lipid metabolism), as shown in Fig. 4J.

In summary, our data demonstrate that silencing TXNIP in MM cells results in reduced growth and viability, alongside increased apoptosis and ROS levels, while TXNIP expression promotes the activation of various pathways related to MM survival, as confirmed by mechanistic studies, RNA-sequencing, and analysis of patient data.

### TXNIP inhibitors reduce MM viability

**Fig. 5:**
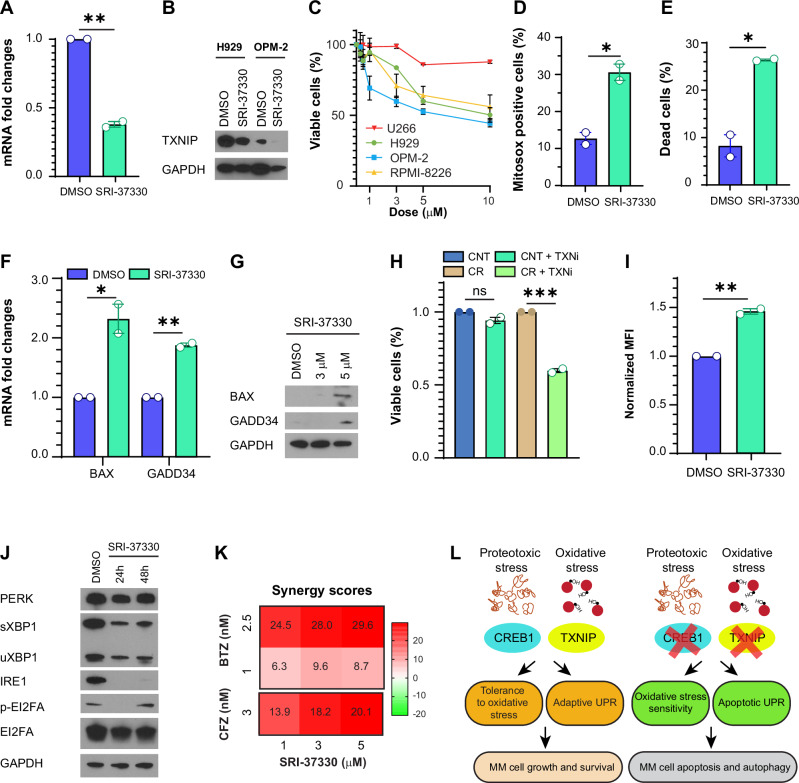
TXNIP inhibitors reduce MM viability. **A** TXNIP mRNA fold changes in OPM-2 cells treated with DMSO or SRI-37330 5 μM for 72 h. Error bars represent the mean ± SEM, *n* = 2 experimental replicates. *p* = 0.0011, **; Student’s *t*-test. **B** Western blot analysis for TXNIP and GAPDH in H929 and OPM-2 cells treated with DMSO or SRI-37330 5 μM for 48 h. **C** Percentage of viable cells in U266, H929, OPM-2, and RPMI-8226 cells treated with DMSO or increasing doses of SRI-37330 for 72 h. Viability is assessed by MTT assay, and the percentage is normalized to the DMSO-treated cells. Error bars represent the mean ± SEM, *n* = 2 experimental replicates. **D** Percentage of Mitosox-positive cells in OPM-2 cells treated with DMSO or SRI-37330 3 μM for 48 h. Error bars represent the mean ± SEM, *n* = 2 experimental replicates. *p* = 0.02, *; Student’s *t*-test. **E** Percentage of dead cells (Annexin V^+^, PI^+^) in OPM-2 cells treated with DMSO or SRI-37330 3 μM for 48 h. Error bars represent the mean ± SEM, *n* = 2 experimental replicates. *p* = 0.016, *; Student’s *t*-test. **F** BAX and GADD34 mRNA fold changes in OPM-2 cells treated with DMSO or SRI-37330 5 μM for 48 h. Error bars represent the mean ± SEM, *n* = 2 experimental replicates. BAX *p* = 0.03, *; GADD34 *p* = 0.0017, **; Student’s *t*-test. **G** Western blot analysis for BAX, GADD34, and GAPDH in OPM-2 cells treated with DMSO or SRI-37330 3 and 5 μM for 48 h. **H** Percentage of viable cells in U266 control cells (CNT), U266 CNT cells treated with 10 μM SRI-37330 (CNT + TXNi), U266 cells overexpressing CREB1 (CR), or U266 cells overexpressing CREB1 treated with 10 μM SRI-37330 (CR + TXNi). The percentage of cells is normalized to each baseline condition (CNT with CNT + TXNi and CR with CR + TXNi). Error bars represent the mean ± SEM, *n* = 2 experimental replicates. CNT versus CNT + TXNi *p* = 0.10, ns; CR versus CR + TXNi *p* = 0.0001, ***. **I** Mean fluorescence intensity (MFI) fold changes of autophagy marker CytoID in OPM-2 cells treated with DMSO or SRI-37330 3 μM for 48 h. Error bars represent the mean ± SEM, *n* = 2 experimental replicates. *p* = 0.0037, **; Student’s *t*-test. **J**. Western blot analysis for PERK, XBP1 (sXBP1 and uXBP1), IRE1, phospho-EI2FA, EI2FA, and GAPDH in OPM-2 cells treated with DMSO or SRI-37330 5 μM for 24 and 48 h. **K** OPM-2 cells treated with DMSO, SRI-37330 1, 3, or 5 μM, bortezomib (BTZ) 1 nM, 2.5 nM, and carfilzomib (CFZ) 3 nM, and all their combinations. Values represent the synergy scores calculated using the mean of two experimental replicates. **L** CREB1 and TXNIP play pivotal roles in mediating tolerance to proteotoxic and oxidative stress in MM cells. In the left panel, CREB1 facilitates the adaptive UPR to manage increased protein load, while TXNIP is crucial for addressing oxidative stress induced by ROS. This results in MM cell growth and survival. In the right panel, silencing or inhibiting CREB1 and TXNIP sensitizes MM cells to oxidative stress, leading to increased ROS levels, autophagy, and apoptosis.

Finally, we evaluated two different TXNIP inhibitors, named TXNIP-IN and SRI-37330. SRI-37330 decreased TXNIP mRNA and protein levels (Fig. 5A, B), showing a dose-effect downregulation (Supplementary Fig. S9A). Treatment with SRI-37330 for 72 h reduced cellular viability (Fig. 5C), increased mitochondrial ROS (Fig. 5D), and induced apoptosis (Fig. 5E) and apoptotic markers such as CASP3, BAX, GADD34, and CHOP (Fig. 5F, G). Conversely, TNXIP-IN did not have any effects on TXNIP expression (Supplementary Fig. S9B, C) or MM cell viability (Supplementary Fig. S9D). To better evaluate the relationship between CREB1 and TXNIP, we performed two sets of experiments. We treated OPM-2 cells with the combination of SRI-37330 (TXNIP inhibitor) and 666-15 (CREB1 inhibitor), and we observed synergy at higher concentrations, when both compounds were able to reduce TXNIP expression (Supplementary Fig. S10A). We then treated U266 control cells or U266 cells overexpressing CREB1 with SRI-37330 at a concentration of 10 μM. While SRI-37330 had no effects in the control cells, it decreased viability in U266 cells overexpressing CREB1 (Fig. 5H). This suggests that while TXNIP is not the only target of CREB1, it contributes to CREB1 pro-tumoral function and its inhibition is particularly effective in the context of CREB1 overexpression. Next, we analyzed other pathways modulated by TXNIP inhibition. SRI-37330 induced autophagy (Fig. 5I), increased mRNA levels of ATG3 and ATG5 (Supplementary Fig. S10B) and protein levels of ULK1, ATG5, ATG7, and LC3 (Supplementary Fig. S10C, D), and reduced phosphorylation and activation of 4EBP1 (Supplementary Fig. S10E). SRI-37330 also decreased the protein levels of both EIF2A-mediated and IRE1-mediated adaptive UPR markers (Fig. 5J and Supplementary Fig. S10F), and increased PERK and total XBP1 mRNA levels as compensatory mechanisms (Supplementary Fig. S10G). Ultimately, we evaluated the combination of SRI-37330 with bortezomib. While the effect of SRI-37330 alone at 48 h was minimal, the combination induced synergic cell death (Fig. 5K).

Taken together, our observations suggest that CREB1 plays a dual role in the control of cellular stressors in MM. On one hand, it promotes mTOR signaling, which leads to increased protein synthesis, as well as the inhibition of autophagy. On the other hand, CREB1 induces the expression of NRF2 and PERK to counterbalance oxidative stress and proteotoxic stress, thereby allowing MM cell survival. In contrast, the inhibition of CREB1 or TXNIP, especially in combination with proteasome inhibitors, is extremely toxic to MM cells due to increased ROS-mediated autophagy and terminal apoptotic UPR (Fig. 5L).

## Discussion

Our data systematically elucidate the roles of CREB1 and TXNIP as essential sensors to maintain the balance between protein production and terminal oxidative responses in MM cells. This regulatory mechanism is especially critical for the survival of fast-proliferating cancer cells, which often operate at their limits due to high energy demands, rapid protein synthesis rates, and the need for genomic variability [1, 2].

We previously demonstrated that CD56 activates CREB1 signaling in MM, without fully elucidating the underlying mechanisms [13]. In this study, we started with the hypothesis that CREB1 may promote MM cell tolerance to oxidative stress, based on its known function in neurons [14]. Notably, RNA-sequencing data from newly diagnosed patients with MM confirmed the enrichment of gene sets associated with UPR and oxidative tolerance in patients with high CREB1 levels. To validate these findings, we overexpressed and silenced CREB1, evaluated specific CREB1 inhibitors, and performed CHIP-sequencing analysis in MM cells. We found that CREB1 binds to the promoters of *NRF2* and *PERK* genes, inducing antioxidant mechanisms and adaptive UPR. In contrast, silencing or pharmacologically inhibiting CREB1 resulted in increased ROS production and apoptosis.

We also identified that CREB1 regulates TXNIP through PERK in MM cells. The *TXNIP* gene is overexpressed and amplified in the 1q cytoband, a region associated with MM progression, hinting at its role in maintaining the redox balance in these cells. While TXNIP typically inhibits thioredoxin, a key antioxidant protein, it paradoxically contributes to ROS clearance and UPR regulation in MM cells. Silencing or pharmacologically inhibiting TXNIP was toxic to MM cells, disrupting the UPR and impairing their ability to manage oxidative stress. Finally, TXNIP inhibition, particularly in the setting of CREB1 overexpression, enhanced the efficacy of targeted combination strategies, supporting its contributory function in CREB1-mediated cellular responses. The role of TXNIP in cancer is complex and varies depending on the cancer type and stage [36]. Notably, TXNIP activates the NLRP3 inflammasome in the presence of ROS [37], leading to secretion of IL-1beta, a driver in MM [38]. Finally, TXNIP helps circumvent intracellular acidification induced by lactate production by reducing glucose intake [39, 40].

Apart from regulating oxidation, CREB1 and TXNIP also activate the mTOR pathway, leading to increased phosphorylation of 4EBP1 and enhanced amino acid metabolism. Taken together, we propose that CREB1 and TXNIP signaling meticulously regulate the balance between protein synthesis and protein homeostasis, preventing adverse effects on MM cells. This regulation is especially critical for cells with rapid kinetics, such as those with high-risk chromosomal abnormalities.

The therapeutic potential of targeting CREB1 in MM cells under oxidative stress remains unexplored. Patients who develop resistance to proteasome inhibitors often have poor outcomes, with NRF2 activation being one contributing mechanism [6]. Inhibiting CREB1 increases ROS levels, reduces NRF2 expression, and, when combined with proteasome inhibitors, induces synergistic apoptosis. This suggests that CREB1 inhibition could be particularly effective in proteasome inhibitor-resistant MMs. Additionally, TXNIP inhibition, especially in the context of CREB1 overexpression, emerges as an alternative strategy, disrupting redox homeostasis and further sensitizing MM cells to targeted therapies.

In conclusion, the study highlights the essential roles of CREB1 and TXNIP in MM cell survival under oxidative and proteotoxic stress, providing new insights into MM pathophysiology. The potential to target these molecules represents a promising avenue for developing novel therapeutic strategies against MM, consistent with the unmet needs of the field.

## Supplementary information

Supplementary methods and figures

Western blot original films

Supplementary Table S1. ChIP-sequencing data of CREB1-promoter binding in H929 cells.

## Data Availability

RNA-sequencing data are available upon request. CHIP-sequencing data are provided as a supplementary file.
