# Supplementary material for: Regulation of stress tolerance by CREB1 sustains multiple myeloma cell survival
Source: Cell Death Dis. 2026 Jan 16;17(1):46. doi: 10.1038/s41419-025-08246-z (PMC12811356; doi:10.1038/s41419-025-08246-z)
Supplement: Supplementary file 1 — Supplementary methods and figures [file 41419_2025_8246_MOESM1_ESM.pdf]

Title:

Regulation of Stress Tolerance by CREB1 Sustains Multiple Myeloma Cell Survival

Authors:

Ruchi Kudalkar, Johnathan Altom, Joshua Galloway, Vincent Manning, Sara Taranto, and Francesca Cottini

[1] Department of Internal Medicine, Division of Hematology, The Ohio State University, Columbus, OH, USA

Correspondence:

Francesca Cottini, MD (lead contact)

Assistant Professor of Medicine

Division of Hematology

The Ohio State University, College of Medicine

385G Wiseman Hall | 400 W 12th Ave |

Columbus OH 43210-1240 USA

Email: Francesca.cottini@osumc.edu

Supplementary Contents

Supplementary Methods

Reagents, Real-Time quantitative PCR primers, antibodies for western blot, Chromatin Immunoprecipitation (ChIP) protocol, plasmid vectors, cellular growth, apoptosis, and viability assays, autophagy and oxidative stress detection assays, immunofluorescence, and proteasome activity assays.

Supplementary Figures and Figure Legends

Supplementary Fig. S1. Gene sets upregulated in patients with high CREB1 expression.

Supplementary Fig. S2. CREB1 inhibition increases production of reactive oxygen species (ROS).

Supplementary Fig. S3. CREB1 modulates genes involved in the tolerance to oxidative stress.

Supplementary Fig. S4. CREB1 affects the mTOR pathway and autophagy.

Supplementary Fig. S5. CREB1 regulates CREB1 regulates proteins involved in the UPR.

Supplementary Fig. S6. CREB1 inhibition potentiates the effects of proteasome inhibitors.

Supplementary Fig. S7. TXNIP expression and regulation in B cells and MM cells.

Supplementary Fig. S8. TXNIP silencing induces cellular death and modulates genes involved in the UPR.

Supplementary Fig. S9. TXNIP-X-IN has minimal activity in MM cells.

Supplementary Fig. S10. SRI-37330, a TXNIP inhibitor, is toxic against MM cells.

Supplementary tables

Supplementary Table. S1. ChIP-sequencing data of CREB1-promoter binding in H929 cells.

Supplementary Methods

Reagents: The following compounds and reagents were used: 666-15 (Tocris, Bio-Techne Corporation, Cat. No. 5661), SRI-37330 (Med Chem Express, Cat. No. HY-141623), TXNIP-IN-1 (Med Chem Express, Cat. No. HY-115688), bortezomib (Selleck Chemicals, Cat. No. S1013), carfilzomib (Selleck Chemicals, Cat. No. S2853), rapamycin (Selleck Chemicals, Cat. No. S1039), and tunicamycin (Selleck Chemicals, Cat. No S7894) in DMSO. Tert-Butyl hydroperoxide solution (Millipore Sigma, Cat. No. 458139) 70 wt. % in water.

RNA primers for quantitative PCR: The following primers were used:

GAPDH_F	5'-GAAGGTGAAGGTCGGAGTCA-3'
GAPDH_R	5'-GGGGTCATTGATGGCAACAATA-3'
CREB1_F	5'-ATTCACAGGAGTCAGTGGATAGT-3'
CREB1_R	5'-CACCGTTACAGTGGTGATGG-3'
TXNIP_F	5'-ATATGGGTGTGTAGACTACTGGG-3'
TXNIP_R	5'-GACATCCACCAGATCCACTAC-3'
BACH1_F	5'-AGTGTAAACTCCGCAGGTATCA-3'
BACH1_R	5'-TTTGGGGCATAAAGAAGGCAA-3'
NRF2_F	5'-TTCCCGGTCACATCGAGAG-3'
NRF2_R	5'-TCCTGTTGCATACCGTCTAAATC-3'
FBXO22_F	5'-GGACCCATCGGAGCGTAAC-3'
FBXO22_R	5'-TACCACGCGAACCAAGCAAT-3'
ATG3_F	5'-GACCCCGGTCCTCAAGGAA-3'
ATG3_R	5'-TGTAGCCCATTGCCATGTTGG-3'
ATG5_F	5'-AAAGATGTGCTTCGAGATGTGT-3'
ATG5_R	5'-CACTTTGTCAGTTACCAACGTCA-3'
ATG7_F	5'-ATGATCCCTGTAACTTAGCCCA-3'
ATG7_R	5'-CACGGAAGCAAACAACTTCAAC-3'
AMPKalpha_F	5'-TTGAAACCTGAAAATGTCCTGCT-3'
AMPKalpha_R	5'-GGTGAGCCACAACTTGTTCTT-3'
TSC1_F	5'-CAACAAGCAAATGTCGGGGAG-3'
TSC1_R	5'-CATAGGGCCACGGTCAGAA-3'
PERK_F	5'-CCAGCCTTAGCAAACCAGA-3'
PERK_R	5'-TCTTGGTCCCACTGGAAGAG-3'
sXBP1_F	5'-CTGAGTCCGAATCAGGTGCAG-3'
sXBP1_R	5'-ATCCATGGGGAGATGTTCTGG-3'
uXBP1_F	5'-CAGCACTCAGACTACGTGCA-3'
uXBP1_R	5'-ATCCATGGGGAGATGTTCTGG-3'

IRE1_F	5'-ATCTGCAAAGGCCGATGA-3'
IRE1_R	5'-GCCGAAGTTCAGATGGAATC-3'
XBP1_TOTAL_F	5'-TGGCCGGGTCTGCTGAGTCCG-3'
XBP1_TOTAL_R	5'-ATCCATGGGGAGATGTTCTGG-3'
CHOP_F	5'-GGCAACAGAGTGGTCATTCCC-3'
CHOP_R	5'-CTGCTTGAGCCGTTCATTCTC-3'
GADD34_F	5'-ATGATGGCATGTATGGTGAGC-3'
GADD34_R	5'-AACCTTGCAGTGTCCTTATCAG-3'
ATF4_F	5'-TCTCCAGCGACAAGGCTAA-3'
ATF4_R	5'-TACCCAACAGGGCATCCA- 3'
CASP3_F	5'-GAAATTGTGGAATTGATGCGTGACA-3'
CASP3_R	5'-CTACAACGATCCCCTCTGAAAAA-3'
BAX_F	5'-CCGAGAGGTCTTTTTCCGAG-3'
BAX_R	5'-GTCCAATGTCCAGCCCATGA-3'

Antibodies for western blot: The following primary antibodies were used: CREB1 (Cell Signaling Technology, Cat. No. 9197, RRID:AB_331277), NRF2 (Cell Signaling Technology, Cat. No. 12721, RRID:AB_2715528), BACH1 (Cell Signaling Technology, Cat. No. 4578, RRID:AB_2061832), TXNIP (Cell Signaling Technology, Cat. No. 14715, RRID:AB_2714178), GAPDH (Cell Signaling Technology, Cat. No. 3683, RRID:AB_1642205), Thioredoxin-TXN (Cell Signaling Technology, Cat. No. 2429, RRID:AB_2272594), FBXO22 (Proteintech, Cat. No. 13606-1-AP, RRID:AB_2104403), TBK1 (Cell Signaling Technology, Cat. No. 3504, RRID:AB_2255663), PRDX1 (Cell Signaling Technology, Cat. No. 8499, RRID:AB_10950824), phospho-mTOR (Ser2448) (Cell Signaling Technology, Cat. No. 5536, RRID:AB_10691552), mTOR (Cell Signaling Technology, Cat. No. 2983, RRID:AB_2105622), phospho-4EBP1 (Thr37/46) (Cell Signaling Technology, Cat. No. 2855, RRID:AB_560835), 4EBP1 (Cell Signaling Technology, Cat. No. 9644, RRID:AB_2097841), phospho-p70S6K (Thr389) (Cell Signaling Technology, Cat. No. 9234, RRID:AB_2269803), p70S6K (Cell Signaling Technology, Cat. No. 2708, RRID:AB_390722), ATG3 (Cell Signaling Technology, Cat. No. 3415, RRID:AB_2059244), ATG5 (Cell Signaling Technology, Cat. No. 12994, RRID:AB_2630393), ATG7 (Cell Signaling Technology, Cat. No. 8558, RRID:AB_10831194), ATG12 (Cell Signaling Technology, Cat. No. 4180, RRID:AB_1903898), LC3B (Cell Signaling Technology, Cat. No. 3868, RRID:AB_2137707), ULK1 (Cell Signaling Technology,

Cat. No. 6439, RRID:AB_11178933), phospho-AMPKalpha (Cell Signaling Technology, Cat. No. 2535, RRID:AB_331250), AMPKalpha (Cell Signaling Technology, Cat. No. 2532, RRID:AB_330331), STK11 (Cell Signaling Technology, Cat. No. 3050, RRID:AB_823559), TSC1 (Cell Signaling Technology, Cat. No. 6935, RRID:AB_10860420), phospho-CaMKK2 (Ser511) (Cell Signaling Technology, Cat. No. 12818, RRID:AB_2798034), PERK (Cell Signaling Technology, Cat. No. 5683, RRID:AB_10841299), phospho-EIF2A (Cell Signaling Technology, Cat. No. 3398, RRID:AB_2096481), EIF2A (Cell Signaling Technology, Cat. No. 5324, RRID:AB_10692650), phospho-IRE1 (Ser724) (Thermo Fisher Scientific, Cat. No. PA1-16927), IRE1 (Cell Signaling Technology, Cat. No. 3294, RRID:AB_823545), XBP1 (Abcam, Cat. No. AB220783, RRID:AB_2920809), Caspase 3 (Cell Signaling Technology, Cat. No. 9662, RRID:AB_331439), ATF4 (Cell Signaling Technology, Cat. No. 11815, RRID:AB_2616025), CHOP (Cell Signaling Technology, Cat. No. 2895, RRID:AB_2089254), and GADD34 (Cell Signaling Technology, Cat. No. 41222). The following secondary antibodies were used: anti-rabbit IgG HRP-linked (Cell Signaling Technology, Cat. No. 7074, RRID:AB_2099233) or anti-mouse IgG HRP-linked (Cell Signaling Technology, Cat. No. 7076, RRID:AB_330924).

Plasmid vectors: For overexpression studies, the following plasmid was used: CREB1 wild-type plasmid (PT3457-5, Cat. No. 631925, Clontech Laboratories, Inc, a Takara Bio Company, Kusatsu, Shiga, Japan) and control vector (pCMV3 control, Cat. No. CV011, Sino Biological Inc). For silencing studies, pLKO.1-TRC control was used as control vector, called pLKO in the manuscript (Addgene plasmid # 10879, RRID: Addgene_10879), while shRNAs against TXNIP (RHS3979-201776846, TRCN0000059059) and CREB1 (RHS3979-201739582, TRCN0000007309) were obtained from Dharmacon.

RNA-sequencing analysis in cell lines: RNA from H929 control cells or H929 cells silenced for TXNIP (3 independent experiments) was extracted using ReliaPrep™ RNA Miniprep Systems (Promega, Cat. No. Z6011). All RNA samples were evaluated for concentration by Qubit® and integrity using Agilent 2100 Bioanalyzer, or the Perkin Elmer LabGX. Standard RNA-Seq (60-80M read pairs) was performed using Illumina NovaSeq 6000 SP PE 150bp format. Service and post-processing of the raw data were

performed by the IGM Genomic Services Lab of The Research Institute at Nationwide Children's Hospital, Columbus, Ohio. Gene sets of Canonical Pathways (C2_CP_BIOCARTA, C2_CP_KEGG, C2_CP_PID, C2_CP_REACTOME, C2_CP_WIKIPATHWAYS) or gene ontology (C5_GOBP) were used to perform enrichment analysis at http://www.broad.mit.edu/gsea. Two-sided p-values <0.05 were considered statistically significant.

Cellular growth assays, apoptosis, and viability assays: Cellular growth was assessed by counting cells over a period of 10 days. 10 µL cells and 10 µL of Trypan blue dye were mixed in a 96-well plate. Cells were counted using a hemocytometer and microscope. The viability of MM cells was evaluated by Thiazolyl Blue tetrazolium bromide, MTT (Sigma-Aldrich, St. Louis, MO, United States) colorimetric survival assay, CellTiter-Glo 2.0 Cell Viability Assay-MTS (Promega, Madison, WI, United States), or LIVE/DEAD™ Fixable Aqua Dead Cell Stain Kit. Regarding the MTT-MTS assays, MM cells (10,000-15,000) were plated in RPMI-1640 supplemented medium, and absorbance or luminescence were read, using a Biotek Cytation 5 Cell Imaging Multi-Mode Reader equipped with Gen5 v3.10 software (Agilent Technologies). Additive and synergistic effects were determined using SynergyFinder+ software (Zheng et al, PMID: 35085776). The percentage of viable cells was also evaluated by LIVE/DEAD™ Fixable Aqua Dead Cell Stain Kit, for 405 nm excitation (Thermo Fisher Scientific, Cat. No. L34966). MM cells were washed in Phosphate Buffered Saline (PBS), stained with specific antibodies for 15 minutes at room temperature, protected from light, washed again, and acquired. The percentage of apoptotic cells was evaluated by Annexin V-propidium iodide (PI) staining (BD Pharmingen, Cat. No. 556547, RRID: AB_2869082). MM cells were washed in PBS, then resuspended in 100 µL of 1X Binding Buffer. 5 µL of FITC Annexin V and 5 uL of PI were added to each sample. Cells were incubated for 15 minutes at room temperature, protected from light. 400 µL of 1X Binding Buffer was added to each tube and samples were acquired on the Attune NxT Flow cytometry machine.

Autophagy assays: CYTO-ID Autophagy kit (Enzo Life Sciences, Cat. No. ENZ-51031) was used to quantify changes in autophagy. MM cell lines treated with DMSO or specific compounds and control cells

or cells silenced for CREB1 and TXNIP were resuspended in 1X Assay Buffer, centrifuged at 1,500 rpm for 5 minutes, and incubated with CYTO-ID Green stain solution for 30 minutes in the dark at room temperature. After a second centrifugation in 1x Assay Buffer, cells were then resuspended in PBS and 10,000 events were acquired per conditions on the Attune NxT Flow cytometry machine. Mean fluorescence intensity was calculated for each condition and normalized to the respective controls (DMSO or pLKO).

Oxidative stress detection assays: CellROX™ Deep Red Reagent (Thermo Fisher Scientific, Cat. No. C10422) was used for measuring the total levels of reactive oxygen species (ROS), while MitoSOX reagent (Thermo Fisher Scientific, Cat. No. M36009) was used to detect mitochondrial superoxide produces generated by oxidation. MM cells in different conditions were harvested and resuspended in RPMI media with fetal bovine serum (FBS). The CellROX® Deep Red reagent was incubated for 45 minutes at 37°C, protected from light, while MitoSOX solution was incubated for 15 min at 37°C, protected from light. Cells were washed once in medium, then in PBS, and acquired on the Attune NxT Flow cytometry machine.

Immunofluorescence: MM cells at baseline conditions were washed in PBS, cytospun at 500 rpm for 5 minutes, fixed with 4% paraformaldehyde (PFA) for 15 min, permeabilized, and incubated with primary antibody against TXNIP (Cell Signaling Technology, Cat. No. 14715, RRID:AB_2714178). After one-hour incubation, cells were washed with PBS, incubated with MitoSOX solution and a secondary fluorescent antibody (Anti-rabbit IgG Alexa Fluor® 488 Conjugate, Cat. No. 4412, RRID:AB_1904025) for 45 minutes and washed again in PBS. ProLong™ Diamond Antifade Mountant with DAPI (Thermo Fisher Scientific, Cat. No. P36966) was added and slides were covered with a coverslip and let dry. Images were acquired using a Nikon A1R Live Cell Confocal microscope and images were analyzed with NIS-Elements (Nikon Instruments Inc) and ImageJ software.

Proteasome activity assay: Proteasome activity assay kit (Abcam, Cat. No. ab107921) was used to measure proteasome activity. Reaction wells were set up according to the protocol. MM cells were

washed with cold PBS and resuspended in 0.5% NP-40. Samples were centrifuged for 15 minutes at 4°C at 13,000 rpm, and the supernatant was transferred to a new tube. Samples were incubated either with or without 1 µL of proteasome inhibitor. 1 µL of proteasome substrate was added to all samples. The plate was incubated at 37 °C, protected from light, for 30 minutes, and then read twice at 30-minute intervals using a fluorometric microplate reader (Ex/Em = 350/440 nm, Biotek Cytation 5 Cell Imaging Multi-Mode Reader, Agilent Technologies).

Protein synthesis assay: Click-iT protein synthesis reagents were used to quantify nascent protein synthesis. MM cell lines treated with DMSO, or specific compounds were cultured for 1 hour in methionine-free RPMI-1640 medium (Thermo Fisher Scientific, Cat. No. A1451701) at 37 °C with 5% CO2. After 1 hour, Click-iT AHA (L-Azidohomoalanine) (Thermo Fisher Scientific, Cat. No. C10102) was added to cells at 25µM concentration and cells were incubated for 1 hour at 37 °C with 5% CO2. Cells were then permeabilized and fixed using Cyto-Fast Fix/Perm Buffer Set (Biolegend, Cat. No. 426803). After fixation and permeabilization, the cells were resuspended in 500µL of Click-iT protein reaction buffer kit (Thermo Fisher Scientific, Cat. No. C10276) and stained for 30 minutes with 1µM Alexa Fluor 488 Alkyne (Thermo Fisher Scientific, Cat. No. A10267) at room temperature protected from light. Negative samples were resuspended in 500µL of Click-iT protein reaction buffer kit with no stain added. After staining, cells were washed centrifuged at 1,500 rpm for 5 minutes in 1x Cyto-Fast Perm Wash solution and then in PBS. Cells were resuspended in PBS and 50,000 events were acquired per condition on the Attune NxT Flow cytometry machine. Mean fluorescence intensity (MFI) was calculated for each condition and normalized to the DMSO control.

Supplementary Fig. S1

A

Pathway	NES	FDR q-value	Gene count
GOBP_CELLULAR_RESPONSE_TO_OXIDATIVE_STRESS	2.08	0.00071	125
BIOCARTA_P38MAPK_PATHWAY	1.99	0.00400	21
WP_OXIDATIVE_STRESS_RESPONSE	1.91	0.00300	19
GOBP_RESPONSE_TO_ENDOPLASMIC_RETICULUM_STRESS	1.84	0.00542	120
BIOCARTA_ARENRF2_PATHWAY	1.68	0.02200	13
GOBP_PERK_MEDIATED_UNFOLDED_PROTEIN_RESPONSE	1.62	0.02569	16
REACTOME_OXIDATIVE_STRESS_INDUCED_SENESCENCE	1.61	0.05200	44

B

Pathway	NES	FDR q-value	Gene count
BIOCARTA_MTOR_PATHWAY	1.98	0.00400	15
GOBP_CELLULAR_RESPONSE_TO_STARVATION	2.22	0.00016	103
KEGG_MTOR_SIGNALING_PATHWAY	2.12	0.00022	31
PID_MTOR_4PATHWAY	2.1	0.00024	36
REACTOME_MTOR_SIGNALLING	1.91	0.00800	15
WP_PI3KAKTMTOR_SIGNALING_PATHWAY_AND_ THERAPEUTIC_OPPORTUNITIES	2.11	0.00016	23

Supplementary Fig. S1. Gene sets upregulated in patients with high CREB1 expression.

(A) Table summarizing all the enriched gene sets shown in **Fig. 1A**. NES, normalized enrichment score. FDR, false discovery rate. Raw data were analyzed from the MMRF CoMMpass database.
(B) Table summarizing all the enriched gene sets shown in **Fig. 1B**. NES, normalized enrichment score. FDR, false discovery rate. Raw data were analyzed from the MMRF CoMMpass database.

Supplementary Fig. S2

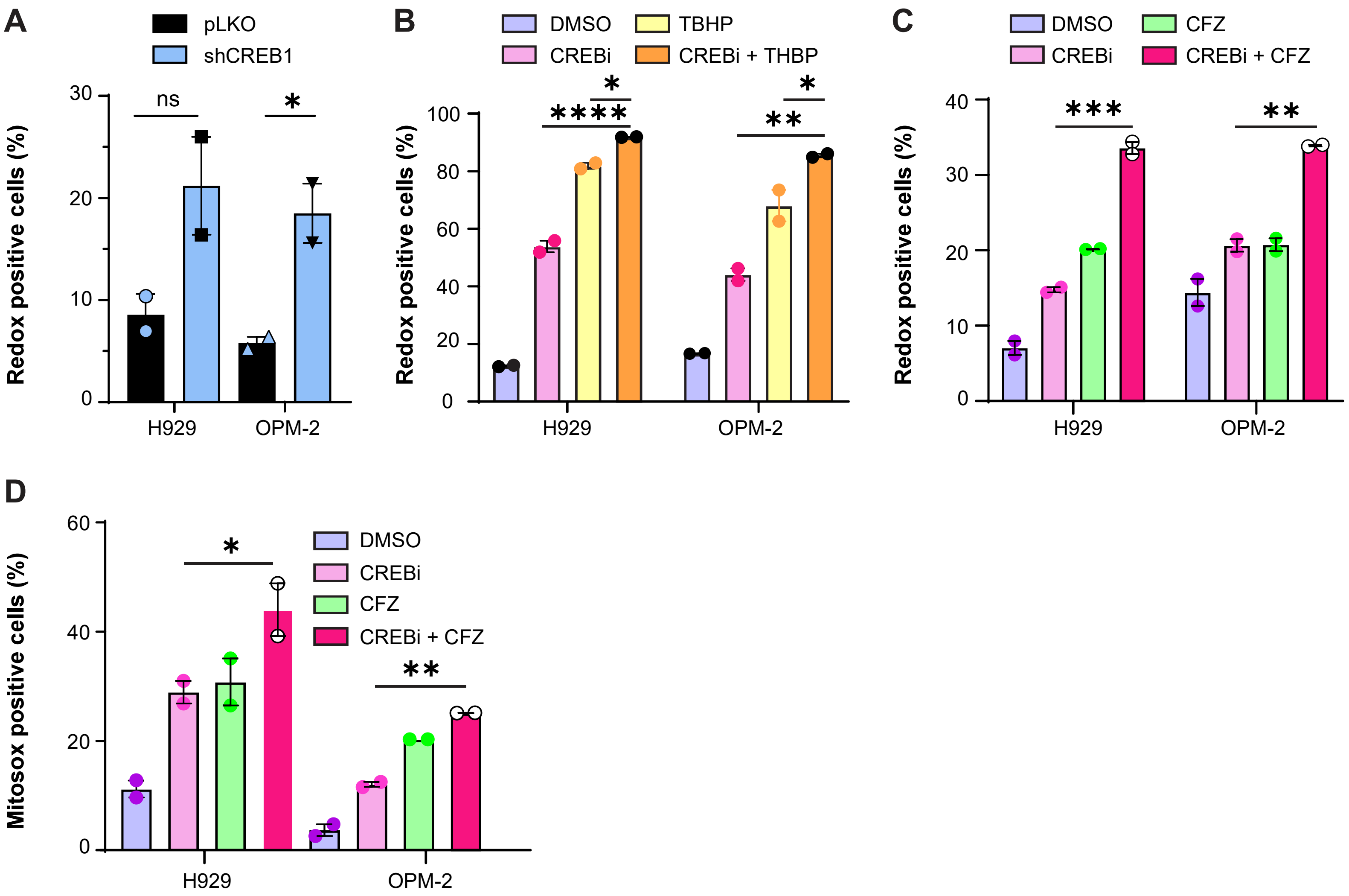

Supplementary Fig. S2. CREB1 inhibition increases the production of reactive oxygen species (ROS).

(A) Percentage of Redox positive cells (marker of total ROS) in H929 and OPM-2 control cells (pLKO) or cells silenced for CREB1 (shCREB1). Error bars represent the mean ± SEM, n = 2 experimental replicates. H929 $p = 0.06$, ns; OPM-2 $p = 0.04$,*; Student's t test. **(B)** Percentage of Redox positive cells in H929 and OPM-2 cells treated for 24 hours with DMSO, 666-15 (CREBi) 0.3 μM, t-Butyl hydroperoxide (TBHP), or CREBi + TBHP. Error bars represent the mean ± SEM, n = 2 experimental replicates. H929: CREBi versus CREBi +TBHP $p < 0.0001$, ****; TBHP versus TBHP + CREBi $p = 0.011$,*; OPM-2: CREBi versus CREBi +TBHP $p = 0.002$,**; TBHP versus TBHP + CREBi $p = 0.04$,*; Tukey's multiple comparison test. **(C)** Percentage of Redox positive cells in H929 and OPM-2 cells treated for 24 hours with DMSO, CREBi 0.3 μM, carfilzomib 5 nM (CFZ), or CREBi + CFZ. Error bars represent the mean ± SEM, n = 2 experimental replicates. H929: CREBi versus CFZ + CREBi $p = 0.0001$, ***; OPM-2: CREBi versus CFZ + CREBi $p = 0.0034$,**; Tukey's multiple comparison test. **(D)** Percentage of Mitosox positive cells (marker of mitochondrial ROS) in H929 and OPM-2 cells treated for 24 hours with DMSO, CREBi 0.3 μM, CFZ 5 nM, or CREBi + CFZ. Error bars represent the mean ± SEM, n = 2 experimental replicates. H929: CREBi versus CFZ + CREBi $p = 0.03$, *; OPM-2: CREBi versus CFZ + CREBi $p = 0.0075$,**; Tukey's multiple comparison test.

Supplementary Fig. S3

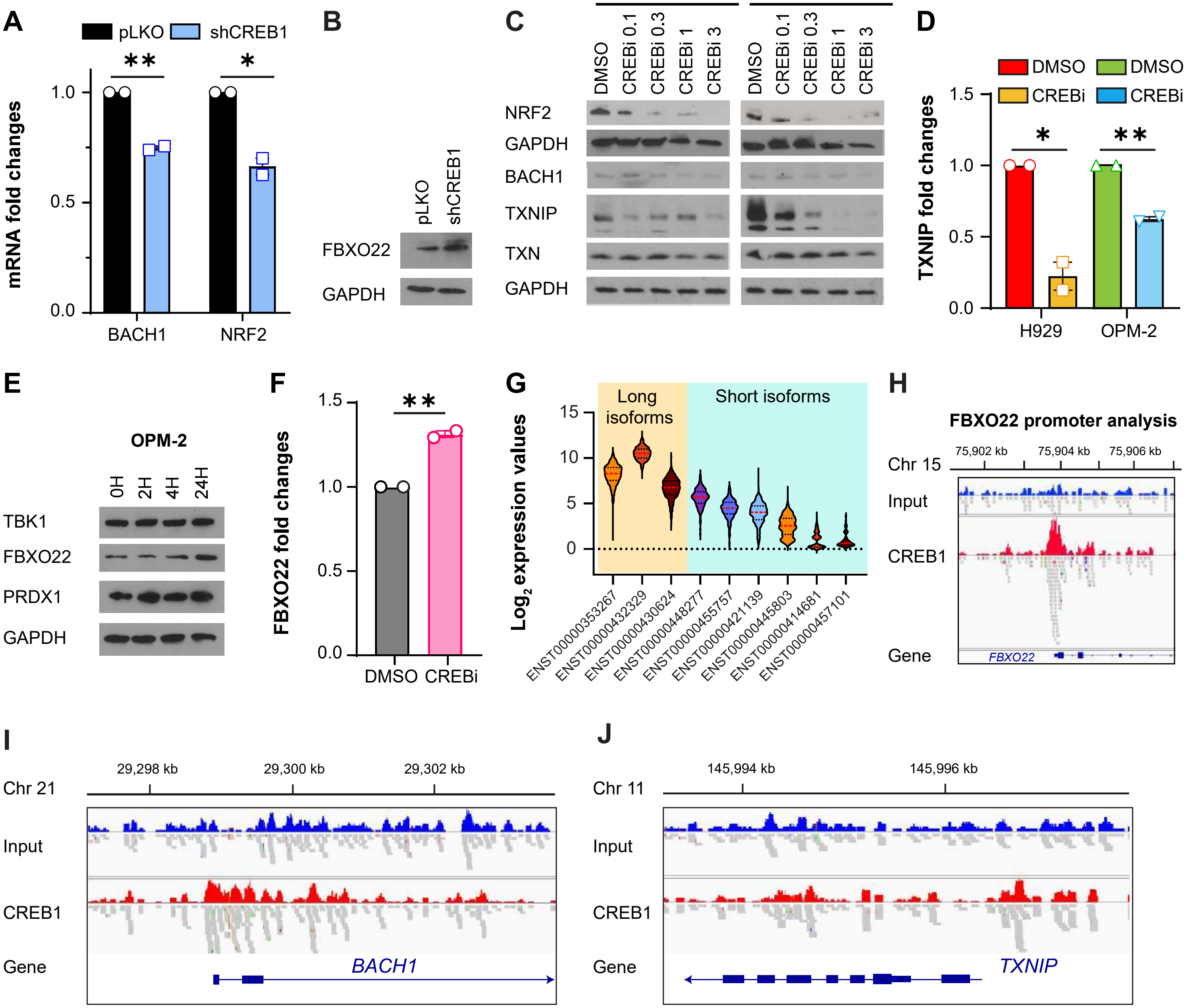

Supplementary Fig. S3. CREB1 modulates genes involved in the tolerance to oxidative stress.

(A) BACH1 and NRF2 mRNA fold changes in OPM-2 control cells (pLKO) or cells silenced for CREB1 (shCREB1). Error bars represent the mean ± SEM, n = 2 experimental replicates. BACH1 $p = 0.0011$,**; NRF2 $p = 0.01$,*; Student's t test. **(B)** Western blot analysis for FBXO22 and GAPDH in OPM-2 control cells (pLKO) or cells silenced for CREB1 (shCREB1). **(C)** Western blot analysis for NRF2, BACH1, TXNIP, TXN, and GAPDH in H929 and OPM-2 cells treated with DMSO or 666-15 (CREBi) 0.1, 0.3, 1, and 3 μM for 48 hours. **(D)** TXNIP mRNA fold changes in H929 and OPM-2 cells treated with DMSO or CREBi 1 μM for 24 hours. Error bars represent the mean ± SEM, n = 2 experimental replicates. H929 $p = 0.046$,*; OPM-2 $p = 0.0018$, **; Student's t test. **(E)** Western blot analysis for TBK1, FBXO22, PRDX1, and GAPDH in OPM-2 cells treated with DMSO or CREBi 1 μM for 2, 4, or 24 hours. **(F)** FBXO22 mRNA fold changes in OPM-2 cells treated with DMSO or CREBi 1 μM for 24 hours. Error bars represent the mean ± SEM, n = 2 experimental replicates. $p = 0.0039$, **; Student's t test. **(G)** CREB1 transcript log_2 expression values in n = 807 patients from the CoMMpass dataset. Dashed red lines represent the median value, dotted black lines represent the 25th and 75th percentile. **(H)** ChIP-seq tracks for CREB1 binding to *FBXO22* promoter. **(J)** ChIP-seq tracks for CREB1 binding to *BACH1* promoter. **(I)** ChIP-seq tracks for CREB1 binding to *TXNIP* promoter.

Supplementary Fig. S4

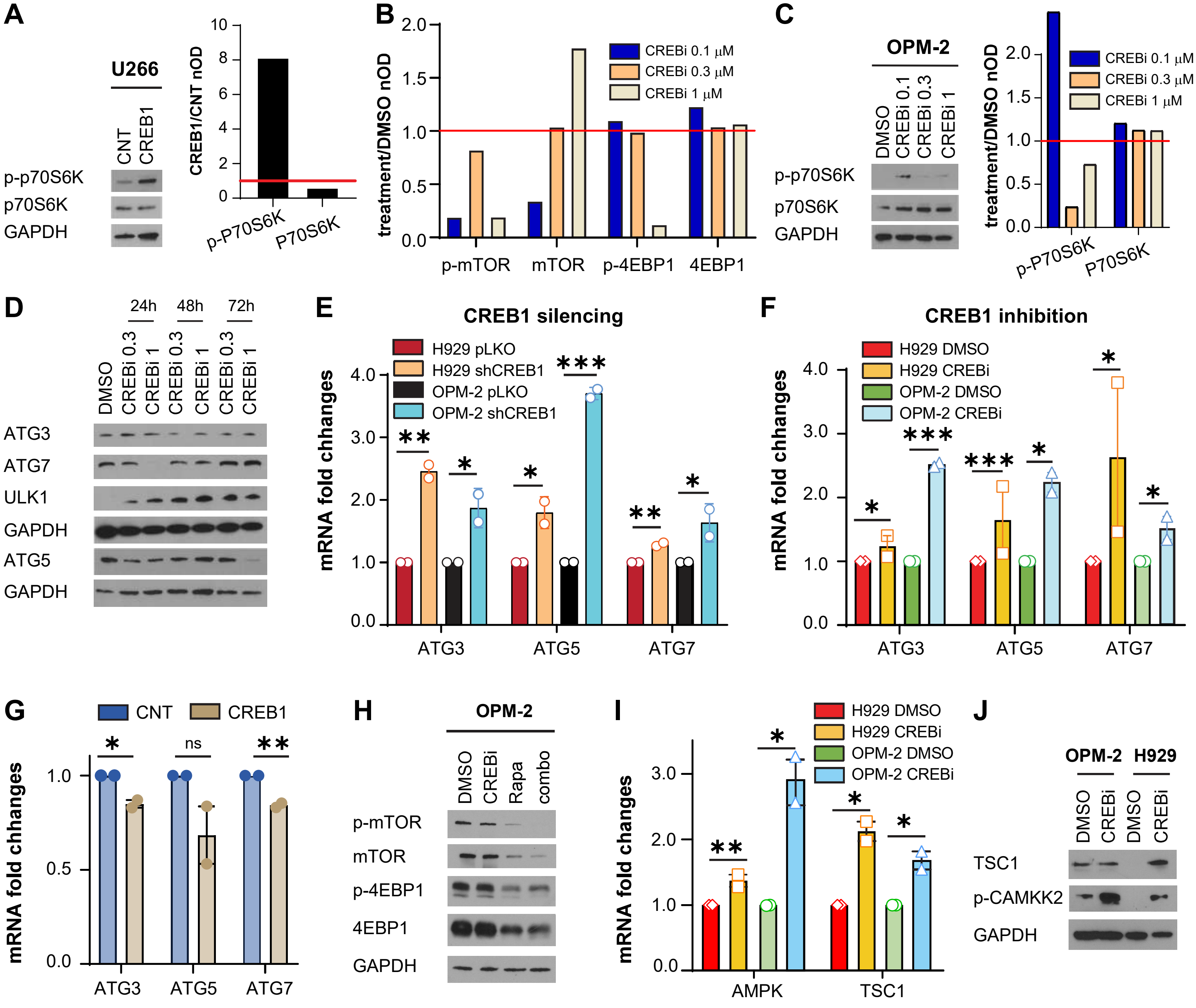

Supplementary Fig. S4. CREB1 affects the mTOR pathway and autophagy.

(A) Western blot analysis for total p70S6K, phospho-p70S6K, and GAPDH in U266 control (CNT) cells or U266 cells overexpressing CREB1. Quantitative densitometry analysis is presented as the ratio of the proteins of interest to GAPDH. The values obtained for CREB1 overexpressing cells are normalized to those of the control cells. nOD, normalized optical density. **(B)** Quantitative densitometry analysis related to Figure 2D. **(C)** Western blot analysis for total p70S6K, phospho-p70S6K, and GAPDH in H929 and OPM-2 cells treated with DMSO or 666-15 (CREBi) 0.1, 0.3, and 1 µM for 48 hours. Quantitative densitometry analysis is presented as the ratio of the proteins of interest to GAPDH. The values obtained for cells treated with various concentrations of the CREBi are normalized to those of the DMSO-treated cells. **(D)** Western blot analysis for ATG3, ATG5, ATG7, ULK1, and GAPDH in OPM-2 cells treated with DMSO or CREBi 0.3 and 1 µM for 24, 48, or 72 hours. **(E)** ATG3, ATG5, and ATG7 mRNA fold changes in H929 and OPM-2 control cells (pLKO), or cells silenced for CREB1 (shCREB1). Error bars represent the mean ± SEM, n = 2 experimental replicates. ATG3: H929 $p = 0.0053$, **; OPM-2 $p = 0.05$, *; ATG5: H929 $p = 0.04$, *; OPM-2 $p = 0.0006$, ***; ATG7: H929 $p = 0.0081$, **; OPM-2 $p = 0.05$, *; Student's t test. **(F)** ATG3, ATG5, and ATG7 mRNA fold changes in H929 and OPM-2 cells treated with DMSO or CREBi 1 µM for 48 hours. Error bars represent the mean ± SEM, n = 2 experimental replicates. ATG3: H929 $p = 0.04$, *; OPM-2 $p = 0.0003$, ***; ATG5: H929 $p = 0.0009$, ***; OPM-2 $p = 0.0152$, *; ATG7: H929 $p = 0.05$, *; OPM-2 $p = 0.05$, *; Student's t test. **(G)** ATG3, ATG5, and ATG7 mRNA fold changes in U266 CNT cells or U266 cells overexpressing CREB1. Error bars represent the mean ± SEM, n = 2 experimental replicates. ATG3 $p = 0.018$, *; ATG5 $p = 0.10$, ns; ATG7 $p = 0.0053$, **; Student's t test. **(H)** Western blot analysis for total mTOR, phospho-mTOR, total 4EBP1, phospho-4EBP1, and GAPDH in OPM-2 cells treated with DMSO, CREBi 1 µM, rapamycin (rapa) 25 nM, or CREBI + rapamycin (combo) for 48 hours. **(I)** AMPKalpha and TSC1 mRNA fold changes in H929 and OPM-2 cells treatedwith DMSO or CREBi 1 µM for 48 hours. Error bars represent the mean ± SEM, n = 2 experimental replicates. H929: AMPKalpha $p = 0.0044$, **; TSC1 $p = 0.017$, *. OPM-2: AMPKalpha $p = 0.031$, *; TSC1 $p = 0.038$, *; Student's t test. **(J)** Western blot analysis for TSC1, phospho-CAMKK2, and GAPDH in H929 and OPM-2 cells treated with DMSO or CREBi 1 µM for 48 hours.

Supplementary Fig. S5

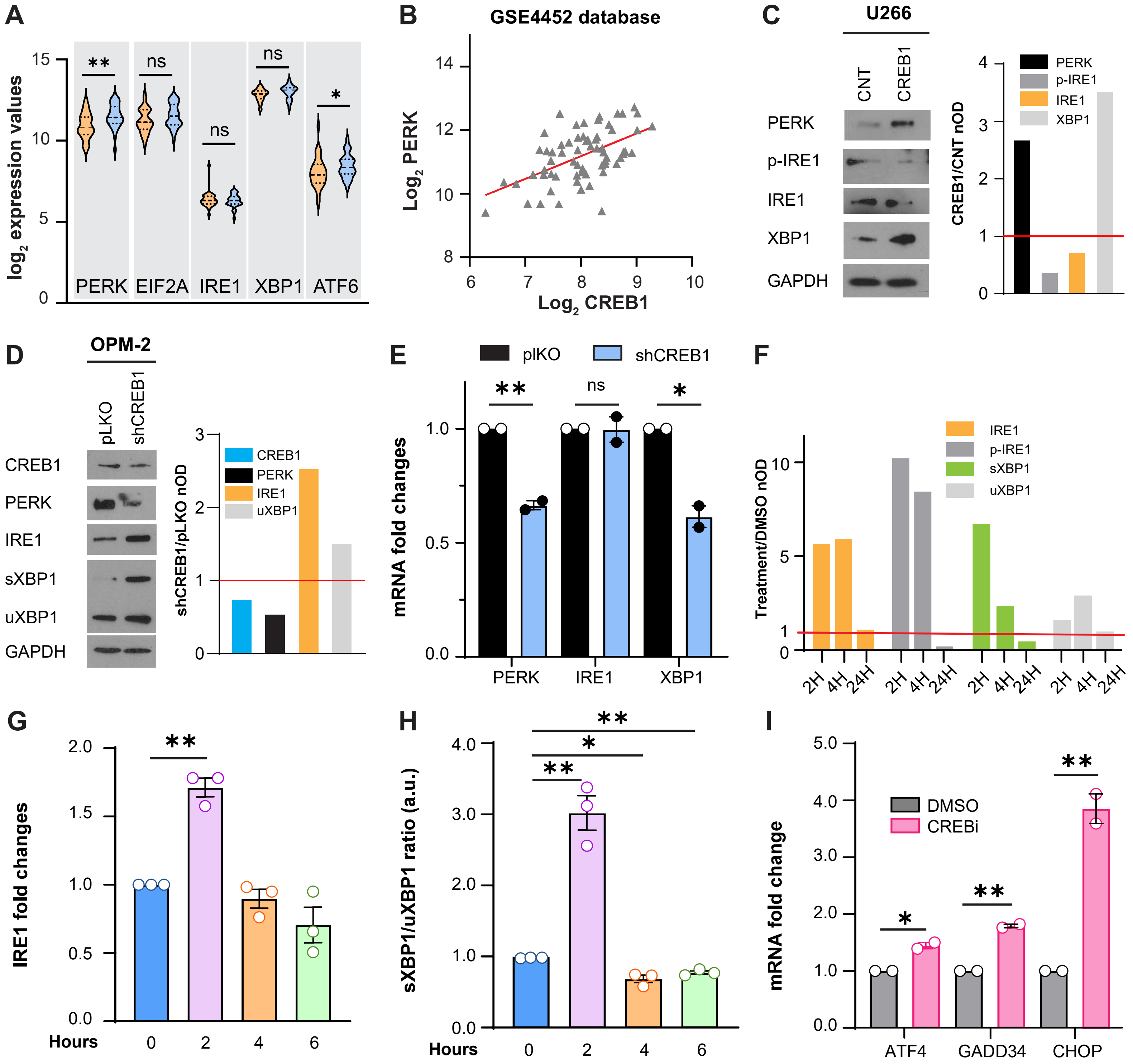

Supplementary Fig. S5. CREB1 regulates proteins involved in the UPR.

(A) PERK, EI2FA, IRE1, XBP1, and ATF6 log_2 expression values based on median CREB1 expression in the GSE4452 database. Black dashed lines represent the median value, black dotted lines represent the 25th and 75th percentile, n = 65 patients. PERK $p = 0.0026$, **; ATF6 $p = 0.05$, *. All other comparisons are not significant, ns, Student's t test. **(B)** Correlation between CREB1 and EIF2AK3 (PERK) levels in the database GSE4452. $p < 0.0001$, R = 0.29; simple linear regression. **(C)** Western blot analysis for PERK, phospho-IRE1, IRE1, XBP1, and GAPDH in U266 control (CNT) cells or U266 cells overexpressing CREB1. Quantitative densitometry analysis is presented as the ratio of the proteins of interest to GAPDH. The values obtained for cells overexpressing CREB1 are normalized to those of the control cells. nOD, normalized optical density. **(D)** Western blot analysis for CREB1, PERK, IRE1, ' XBP1 (sXBP1 and uXBP1), and GAPDH in OPM-2 control cells (pLKO) or cells silenced for CREB1 (shCREB1). Quantitative densitometry analysis is presented as the ratio of the proteins of interest to GAPDH. The values obtained for cells silenced for CREB1 arenormalized to those of the control cells. **(E)** PERK, IRE1, and total XBP1 mRNA fold changes in OPM-2 control cells (pLKO) and cells silenced for CREB1 (shCREB1). Error bars represent the mean ± SEM, n = 2 experimental replicates. PERK $p = 0.0034$, **; IRE1 $p = 0.95$, ns; total XBP1 $p = 0.01$,*; Student's t test. **(F)** Quantitative densitometry analysis related to Figure 3H. **(G)** IRE1 mRNA fold changes in OPM-2 cells treated with DMSO or CREBi 1 μM for 2, 4, and 6 hours. Error bars represent the mean ± SEM, n = 3 experimental replicates. DMSO versus 2h $p = 0.0026$, **; DMSO versus 4h $p = 0.09$, ns; DMSO versus 6h $p = 0.08$, ns; Tukey's multiple comparison test. **(H)** sXBP1/uXBP1 ratio in OPM-2 cells treated with DMSO or CREBi 1 μM for 2, 4, and 6 hours. Error bars represent the mean ± SEM, n = 3 experimental replicates. DMSO versus 2h $p = 0.0074$, **; DMSO versus 4h $p = 0.0211$, *; DMSO versus 6h $p = 0.0053$, **; Tukey's multiple comparison test. **(I)** ATF4, CHOP, and GADD34 mRNA fold changes in OPM-2 cells treated with DMSO or CREBi 1 μM for 24 hours. Error bars represent the mean ± SEM, n = 2 experimental replicates. ATF4 $p = 0.014$,*; GADD34 $p = 0.0014$, **; CHOP $p = 0.0082$,**; Student's t test.

Supplementary Fig. S6

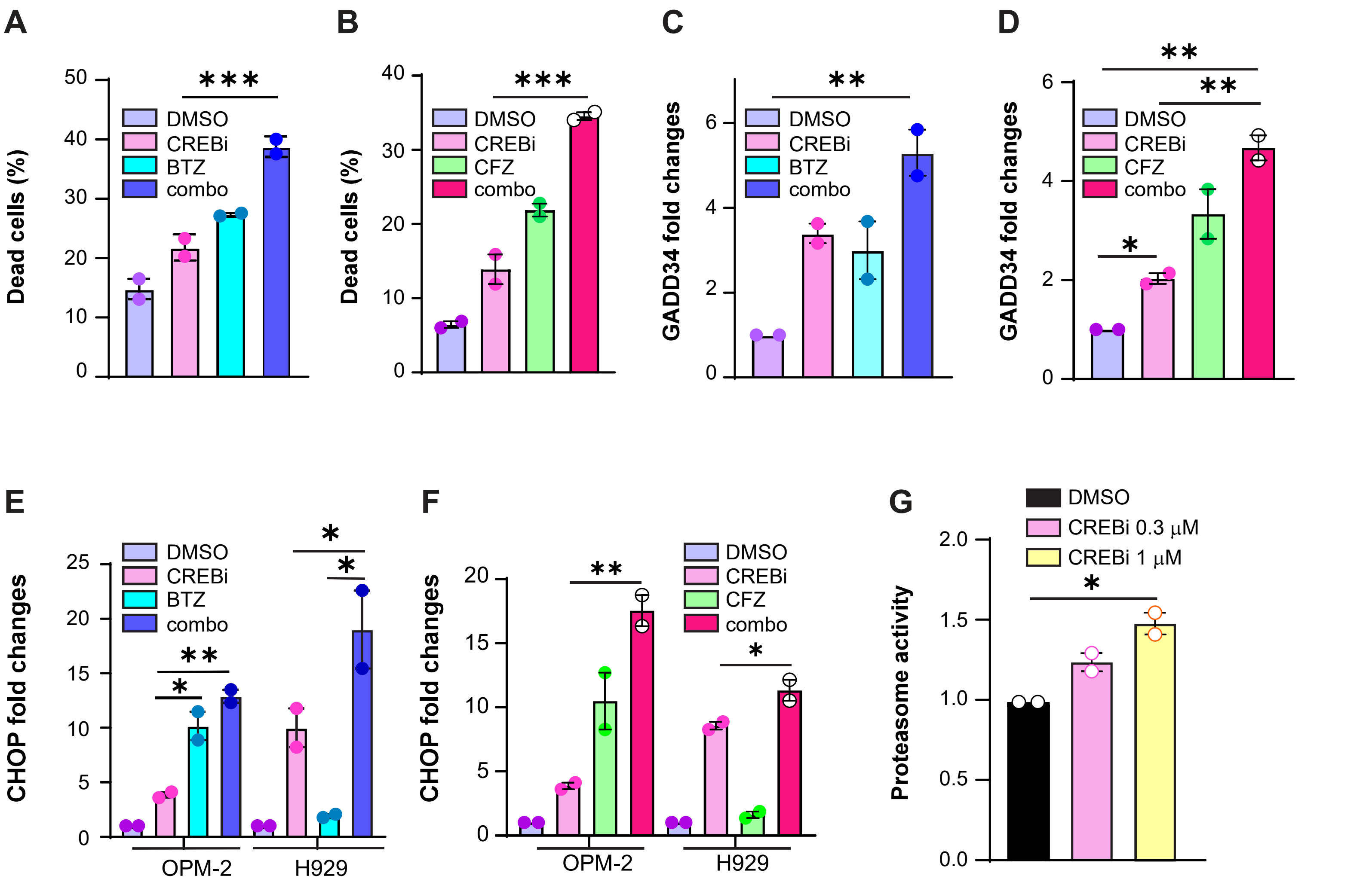

Supplementary Fig. S6. CREB1 inhibition potentiates the effects of proteasome inhibitors.

(A) Percentage of dead cells (Zombie Aqua positive) in OPM-2 cells treated with DMSO, 666-15 (CREBi) 0.3 μM, bortezomib (BTZ) 5 nM, or CREBi + BTZ (combo) for 24 hours. Error bars represent the mean ± SEM, n = 2 experimental replicates. CREBi versus CREBi + BTZ p = 0.0003, ***; Tukey's multiple comparison test. **(B)** Percentage of dead cells in OPM-2 cells treated with DMSO, CREBi 0.3 μM, carfilzomib (CFZ) 12.5 nM, or CREBi + CFZ (combo) for 24 hours. Error bars represent the mean ± SEM, n = 2 experimental replicates. CREBi versus combo p = 0.0008,***; Tukey's multiple comparison test. **(C)** GADD34 mRNA fold changes in OPM-2 cells treated with DMSO, CREBi 0.3 μM, BTZ 5 nM, or CREBi + BTZ (combo) for 24 hours. Error bars represent the mean ± SEM, n = 2 experimental replicates. DMSO versus combo p = 0.0087, **; Tukey's multiple comparison test. **(D)** GADD34 mRNA fold changes in OPM-2 cells treated with DMSO, CREBi 0.3 μM, CFZ 5 nM, or CREBi + CFZ (combo) for 24 hours. Error bars represent the mean ± SEM, n = 2 experimental replicates. DMSO versus CFZ p = 0.015, *; DMSO versus combo p = 0.0028, **; CREBi versus combo p = 0.0096, **; Tukey's multiple comparison test. **(E)** CHOP mRNA fold changes in OPM-2 and H929 treated with DMSO, CREBi 0.3 μM, BTZ 5 nM, or CREBi + BTZ (combo) for 24 hours. Error bars represent the mean ± SEM, n = 2 experimental replicates. OPM-2: CREBi versus BTZ p = 0.01, *; CREBi versus combo p = 0.0031, **. H929: BTZ versus combo p = 0.0128, *; CREBi versus combo p = 0.01, *; Tukey's multiple comparison test. **(F)** CHOP mRNA fold changes in OPM-2 and H929 cells treated with DMSO, CREBi 0.3 μM, CFZ 5 nM, or CREBi + CFZ (combo) for 24 hours. Error bars represent the mean ± SEM, n = 2 experimental replicates. OPM-2: CREBi versus combo p = 0.0056, **; H929: CREBi versus combo p = 0.0413,*; Tukey's multiple comparison test. **(G)** Normalized proteasome activity in OPM-2 cells treated for 24 hours with DMSO or CREBi 0.3 μM and 1 μM. Error bars represent the mean ± SEM, n = 2 experimental replicates. DMSO versus CREBi 0.3 μM p = 0.06, ns; DMSO versus CREBi 1 μM p = 0.01,*; Tukey's multiple comparison test.

Supplementary Fig. S7

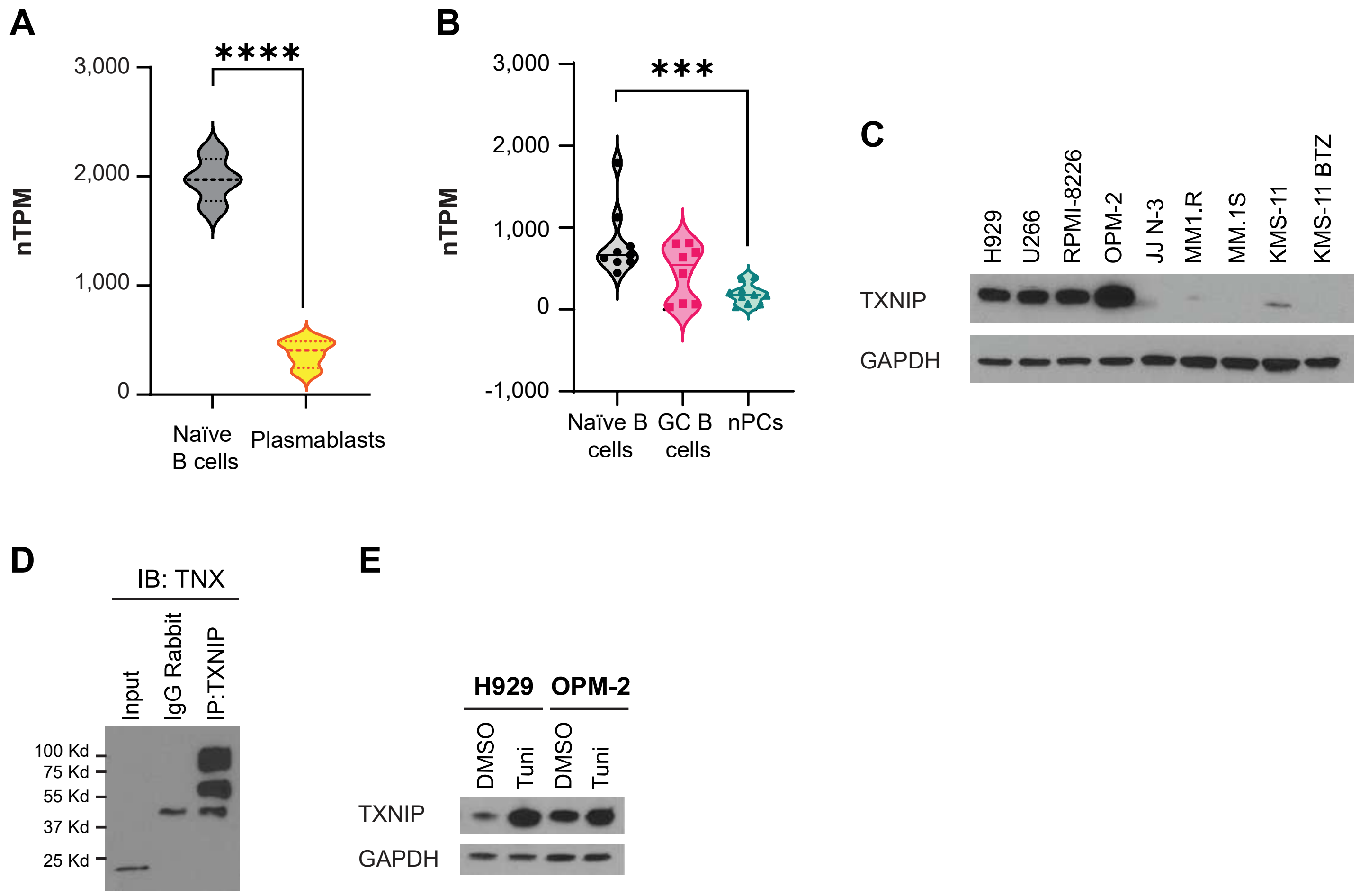

Supplementary Fig. S7. TXNIP expression and regulation in B cells and MM cells.

A. TXNIP RNA-sequencing expression values in naïve B cells (n = 4) and plasmablasts (n = 4) from healthy donors. Dashed lines represent the median value, dotted lines represent the 25th and 75th percentile. Data are from Monaco et al. $p < 0.0001$, ****; Student's t test.

B. TXNIP RNA-sequencing expression values in naïve B cells (n = 9), germinal center (GC) B cells (n = 8), and normal plasma cells (nPCs) (n = 10) from healthy donors in our dataset. Solid lines represent the median value. Naïve B cells versus nPCs $p = 0.0006$, ***; Student's t test.

C. Western blot analysis for TXNIP and GAPDH in a panel of MM cell lines.

D. Immunoprecipitation (IP) for TXNIP in OPM-2 cells and blot (IB) for TNX. Line 1 is total lysates (input), line 2 is IgG rabbit alone, and line 3 is TXNIP-IP (immunoprecipitate).

E. Western blot analysis for TXNIP and GAPDH in H929 and OPM-2 cells treated with DMSO or tunicamycin (Tuni) 1 μM for 24 hours.

Supplementary Fig. S8

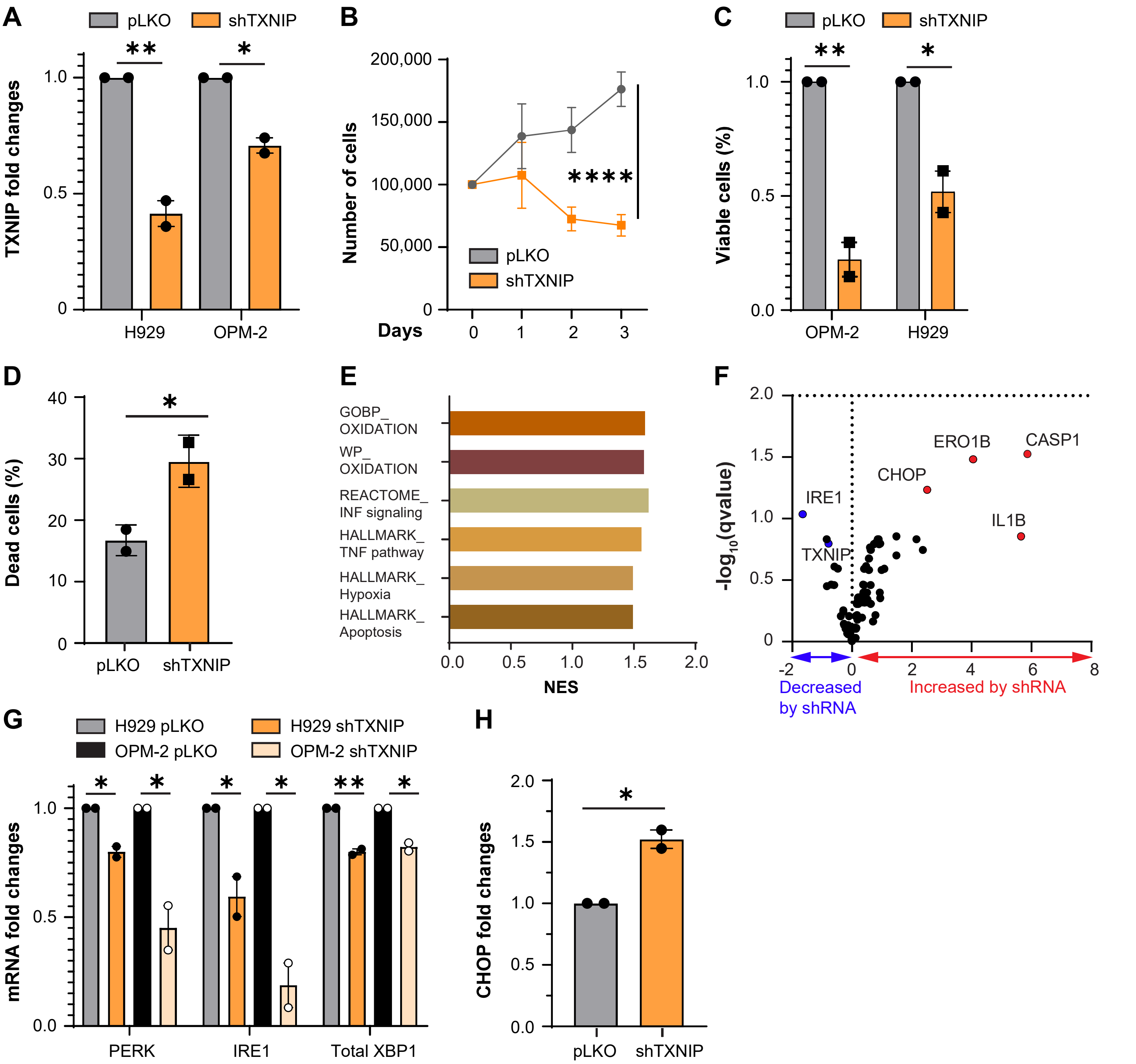

Supplementary Fig. S8. TXNIP silencing induces cellular death and modulates genes involved in the UPR.

(A) TXNIP mRNA fold changes in H929 and OPM-2 control cells (pLKO) or cells silenced for TXNIP (shTXNIP). Error bars represent the mean ± SEM, n = 2 experimental replicates. H929 p = 0.008,**; OPM-2 p = 0.01,*; Student's t test. **(B)** Cellular growth in OPM-2 control cells (pLKO) or cells silenced for TXNIP (shTXNIP). Error bars represent the mean ± SD, n = 2 experimental replicates. 48 hours p = 0.0004,***; 72 hours p < 0.0001, ****; Student's t test. **(C)** Percentage of viable cells in OPM-2 and H929 control cells (pLKO) or cells silenced for TXNIP (shTXNIP) at 72 hours. Viability is assessed by MTT assay and percentage is normalized to the control cells. Error bars represent the mean ± SEM, n = 2 experimental replicates. OPM-2 p = 0.0092, **; H929 p = 0.0338, *; Student's t test. **(D)** Percentage of dead cells (Annexin V$^+$, PI$^+$) in OPM-2 control cells (pLKO) or cells silenced for TXNIP (shTXNIP). Error bars represent the mean ± SEM, n = 2 experimental replicates. p = 0.05, *; Student's t test. **(E)** Gene signature enrichment analysis in H929 cells silenced for TXNIP versus H929 control cells (pLKO). Normalized Enrichment Score (NES) values are plotted. False discovery rate <25% was used as threshold for enrichment. n = 3 experimental replicates. **(F)** Volcano plot of UPR-related genes. In red are highlighted the top significantly upregulated genes, in blue the top significantly downregulated genes. n = 3 experimental replicates. **(G)** PERK, IRE1, and total XBP1 mRNA fold changes in H929 and OPM-2 pLKO cells or cells silenced for TXNIP (shTXNIP). Error bars represent the mean ± SEM, n = 2 experimental replicates. PERK: H929 p = 0.014, *; OPM-2 p = 0.032, *; IRE1: H929 p = 0.04, *; OPM-2 p = 0.01, *; Total XBP1: H929 p = 0.004, **; OPM-2 p = 0.01, *; Student's t test. **(H)** CHOP mRNA fold changes in H929 control cells (pLKO) or cells silenced for TXNIP (shTXNIP). Error bars represent the mean ± SEM, n = 2 experimental replicates. p = 0.02, *; Student's t test.

Supplementary Fig. S9

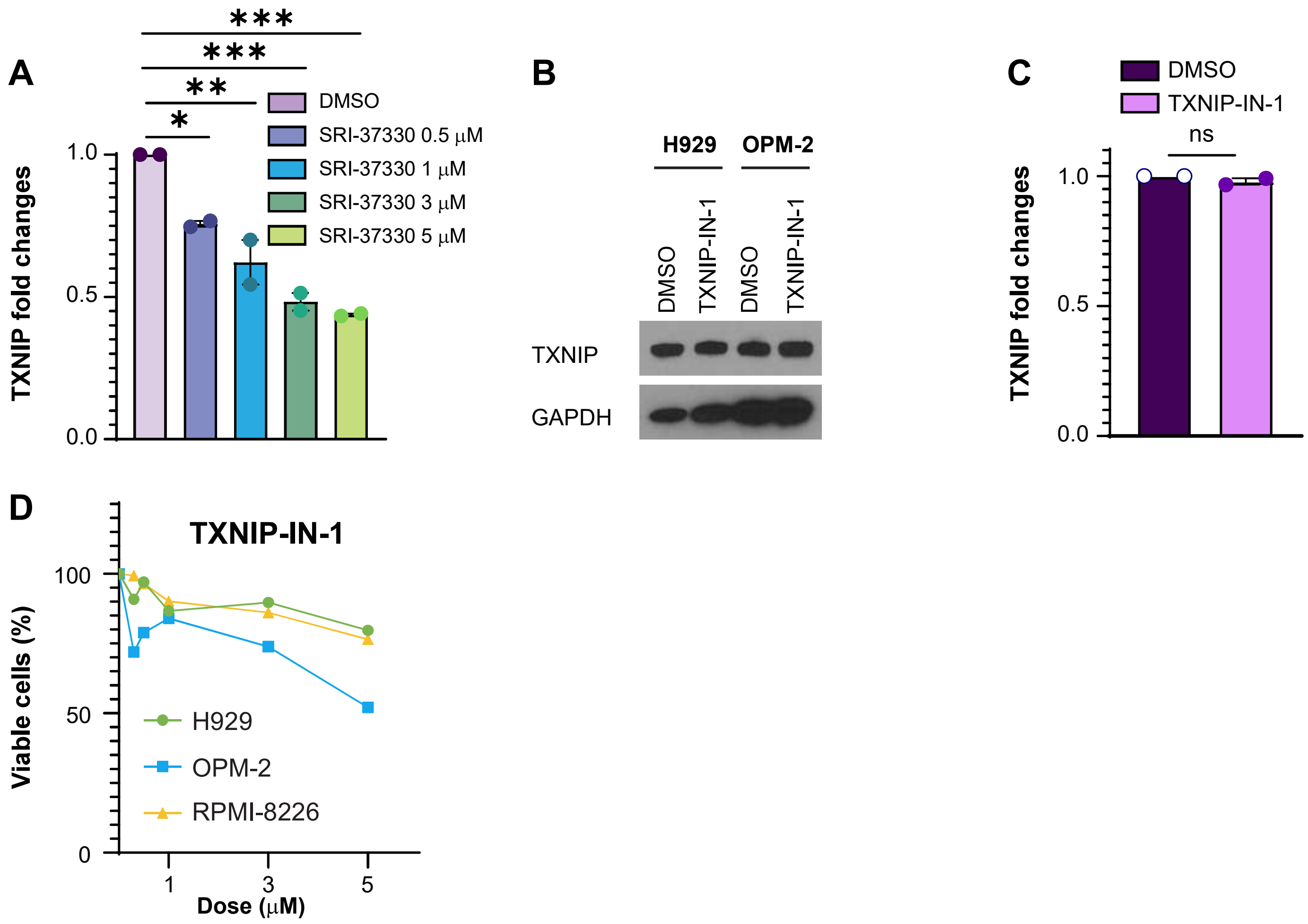

Supplementary Fig. S9. TTXNIP-X-IN has minimal activity in MM cells.

(A) TXNIP mRNA fold changes in OPM-2 cells treated with DMSO or SRI-37330 0.5, 1, 3, and 5 μM for 48 hours. Error bars represent the mean ± SEM, n = 2 experimental replicates. DMSO versus SRI-37330 0.5 μM p = 0.018, *; DMSO versus SRI-37330 1 μM p = 0.0027, **; DMSO versus SRI-37330 3 μM p = 0.0006, ***; DMSO versus SRI-37330 5 μM p = 0.0004, ***; Tukey's multiple comparison test. **(B)** Western blot analysis for TXNIP and GAPDH in H929 and OPM-2 cells treated with DMSO or TXNIP-IN-1 5 μM for 48 hours. **(C)** TXNIP mRNA fold changes in OPM-2 cells treated with DMSO or TXNIP-IN-1 5 μM for 72 hours. Error bars represent the mean ± SEM, n = 2 experimental replicates. p = 0.22, ns, not significant; Student's t test. **(D)** Percentage of viable cells in H929, OPM-2, and RPMI-8226 treated with DMSO or increasing doses of TXNIP-IN for 72 hours. Viability is assessed by MTT assay and percentage is normalized to the control cells. n = 2 experimental replicates.

Supplementary Fig. S10

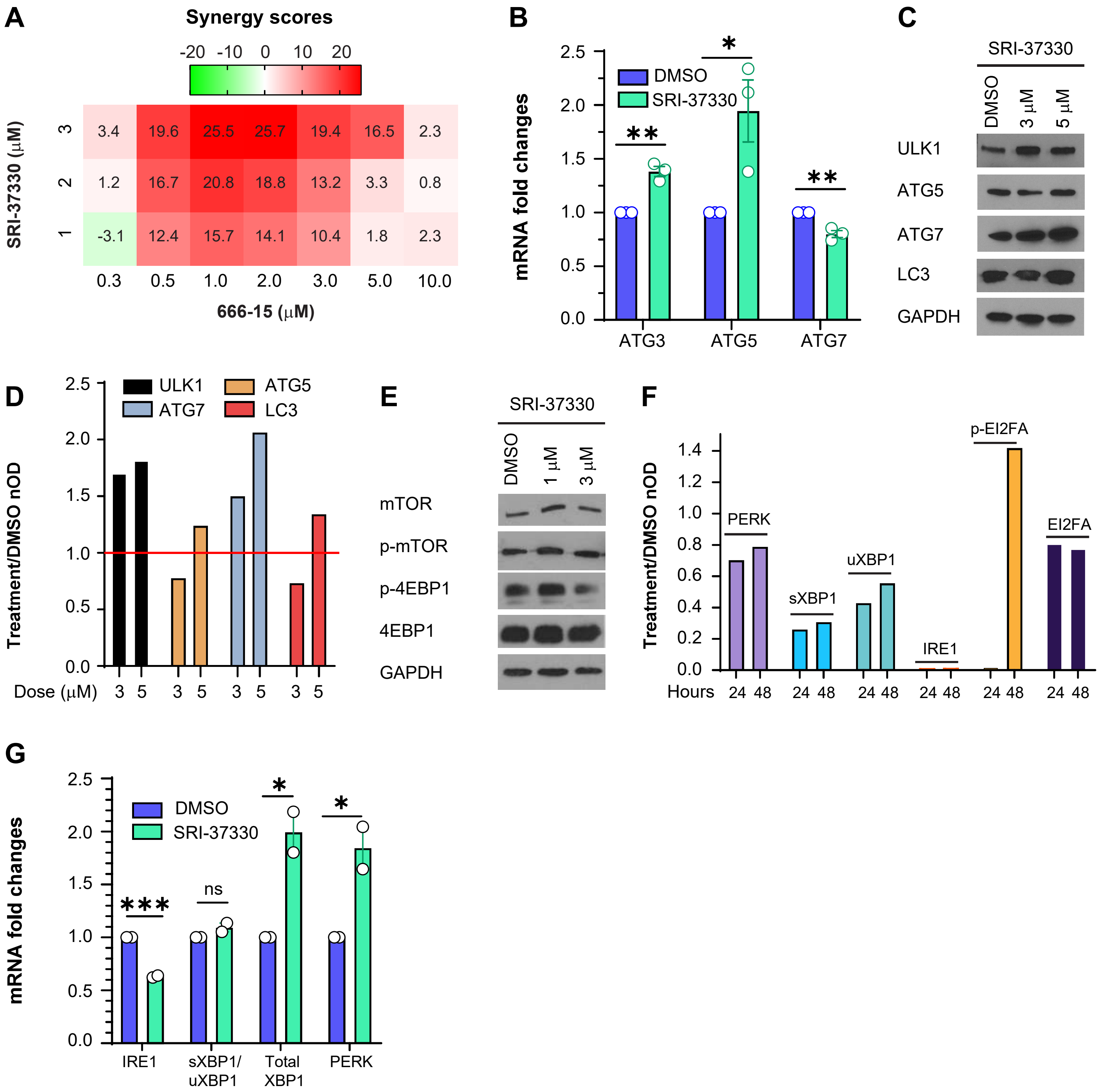

Supplementary Fig. S10. SRI-37330, a TXNIP inhibitor, is toxic against MM cells.

(A) OPM-2 cells treated with DMSO, SRI-37330 0, 1, 2, or 3 μM, 666-15 0-10 μM, and all their combinations. Values represent the synergy scores calculated using the mean of n =2 experimental replicates. **(B)** ATG3, ATG5, and ATG7 mRNA fold changes in OPM-2 cells treated with DMSO or SRI-37330 3 μM for 48 hours. Error bars represent the mean ± SEM, n = 3 experimental replicates. ATG3 *p* = 0.0013, **; ATG5 *p* = 0.03, *; ATG7 *p* = 0.003, **; Student's t test. **(C)** Western blot analysis for ULK1, ATG5, ATG7, LC3, and GAPDH in OPM-2 cells treated with DMSO or SRI-37330 3 and 5 μM for 48 hours. **(D)** Quantitative densitometry analysis for panel **C** is presented as the ratio of the proteins of interest to GAPDH. The values obtained for cells treated with SRI-37330 are normalized to those of the DMSO-treated cells. nOD, normalized optical density. **(E)** Western blot analysis for total MTOR, phospho-mTOR, total 4EBP1, phospho-4EBP1, and GAPDH in OPM-2 cells treated with DMSO or SRI-37330 1 and 3 μM for 48 hours. **(F)** Quantitative densitometry analysis for **figure 5J** is presented as the ratio of the proteins of interest to GAPDH. The values obtained for cells treated with SRI-37330 are normalized to those of the DMSO-treated cells. **(G)** IRE1, sXBP1/uXBP1 ratio, total XBP1, and PERK mRNA fold changes in OPM-2 cells treated with DMSO or SRI-37330 3 μM for 48 hours. Error bars represent the mean ± SEM, n = 2 experimental replicates. IRE1 *p* = 0.0007, ***; sXBP1/uXBP1 ratio *p* = 0.16, ns; total XBP1 *p* = 0.03, *; PERK *p* = 0.05,*; Student's t test.

Supplementary figures legends:

Supplementary Fig. S1. Gene sets upregulated in patients with high CREB1 expression.

A. Table summarizing all the enriched gene sets shown in **Fig. 1A**. NES, normalized enrichment score. FDR, false discovery rate. Raw data were analyzed from the MMRF CoMMpass database.

B. Table summarizing all the enriched gene sets shown in **Fig. 1B**. NES, normalized enrichment score. FDR, false discovery rate. Raw data were analyzed from the MMRF CoMMpass database.

Supplementary Fig. S2. CREB1 inhibition increases the production of reactive oxygen species (ROS).

A. Percentage of Redox positive cells (marker of total ROS) in H929 and OPM-2 control cells (pLKO), or cells silenced for CREB1 (shCREB1). Error bars represent the mean ± SEM, n = 2 experimental replicates. H929 *p* = 0.06, ns; OPM-2 *p* = 0.04, *; Student's t test.

B. Percentage of Redox positive cells in H929 and OPM-2 cells treated for 24 hours with DMSO, 666-15 (CREBi) 0.3 μM, t-Butyl hydroperoxide (TBHP), or CREBi + TBHP. Error bars represent the mean ± SEM, n = 2 experimental replicates. H929: CREBi versus CREBi +TBHP *p* < 0.0001, ****; TBHP versus TBHP + CREBi *p* = 0.011, *; OPM-2: CREBi versus CREBi +TBHP *p* = 0.002, **; TBHP versus TBHP + CREBi *p* = 0.04, *; Tukey's multiple comparison test.

C. Percentage of Redox positive cells in H929 and OPM-2 cells treated for 24 hours with DMSO, CREBi 0.3 μM, carfilzomib 5 nM (CFZ), or CREBi + CFZ. Error bars represent the mean ± SEM, n = 2 experimental replicates. H929: CREB1 versus CFZ + CREBi *p* = 0.0001, ***; OPM-2: CREB1 versus CFZ + CREBi *p* = 0.0034, **; Tukey's multiple comparison test.

D. Percentage of Mitosox positive cells (marker of mitochondrial ROS) in H929 and OPM2 cells treated for 24 hours with DMSO, CREBi 0.3 μM, CFZ 5 nM, or CREBi + CFZ. Error bars represent the mean ± SEM, n = 2 experimental replicates. H929: CREBi versus CFZ + CREBi *p* = 0.03, *; OPM-2: CREBi versus CFZ + CREBi *p* = 0.0075, **; Tukey's multiple comparison test.

Supplementary Fig. S3. CREB1 modulates genes involved in the tolerance to oxidative stress.

A. BACH1 and NRF2 mRNA fold changes in OPM-2 control cells (pLKO), or cells silenced for CREB1 (shCREB1). Error bars represent the mean ± SEM, n = 2 experimental replicates. BACH1 *p* = 0.0011, **; NRF2 *p* = 0.01, *; Student's t test.

B. Western blot analysis for FBXO22 and GAPDH in OPM-2 control cells (pLKO), or cells silenced for CREB1 (shCREB1).

C. Western blot analysis for NRF2, BACH1, TXNIP, TXN, and GAPDH in H929 and OPM-2 cells treated with DMSO or 666-15 (CREBi) 0.1, 0.3, 1, and 3 μM for 48 hours.

D. TXNIP mRNA fold changes in H929 and OPM-2 cells treated with DMSO or CREBi 1 μM for 24 hours. Error bars represent the mean ± SEM, n = 2 experimental replicates. H929 *p* = 0.046, *; OPM-2 *p* = 0.0018, **; Student's t test.

E. Western blot analysis for TBK1, FBXO22, PRDX1, and GAPDH in OPM-2 cells treated with DMSO or CREBi 1 μM for 2, 4, or 24 hours.

F. FBXO22 mRNA fold changes in OPM-2 cells treated with DMSO or CREBi 1 μM for 24 hours. Error bars represent the mean ± SEM, n = 2 experimental replicates. *p* = 0.0039, **; Student's t test.

G. CREB1 transcript log_2 expression values in n = 807 patients from the CoMMpass dataset. Dashed red lines represent the median value, dotted black lines represent the 25th and 75th percentile.

H. ChIP-seq tracks for CREB1 binding to *FBXO22* promoter.

I. ChIP-seq tracks for CREB1 binding to *BACH1* promoter.

J. ChIP-seq tracks for CREB1 binding to *TXNIP* promoter.

Supplementary Fig. S4. CREB1 affects the mTOR pathway and autophagy.

A. Western blot analysis for total p70S6K, phospho-p70S6K, and GAPDH in U266 control (CNT) cells or U266 cells overexpressing CREB1. Quantitative densitometry analysis is presented as the ratio of the proteins of interest to GAPDH. The values obtained for CREB1 overexpressing cells are normalized to those of the control cells. nOD, normalized optical density.

B. Quantitative densitometry analysis related to **Figure 2D.**

C. Western blot analysis for total p70S6K, phospho-p70S6K, and GAPDH in H929 and OPM-2 cells treated with DMSO or 666-15 (CREBi) 0.1, 0.3, and 1 μM for 48 hours. Quantitative densitometry analysis is presented as the ratio of the proteins of interest to GAPDH. The values obtained for cells treated with various concentrations of the CREBi are normalized to those of the DMSO-treated cells.

D. Western blot analysis for ATG3, ATG5, ATG7, ULK1, and GAPDH in OPM-2 cells treated with DMSO or CREBi 0.3 and 1 μM for 24, 48, or 72 hours.

E. ATG3, ATG5, and ATG7 mRNA fold changes in H929 and OPM-2 control cells (pLKO), or cells silenced for CREB1 (shCREB1). Error bars represent the mean ± SEM, n = 2 experimental replicates. ATG3: H929 p = 0.0053, **; OPM-2 p = 0.05, *; ATG5: H929 p =

0.04, *; OPM-2 p = 0.0006, ***; ATG7: H929 p = 0.0081, **; OPM-2 p = 0.05, *; Student's t test.

F. ATG3, ATG5, and ATG7 mRNA fold changes in H929 and OPM-2 cells treated with DMSO or CREBi 1 μM for 48 hours. Error bars represent the mean ± SEM, n = 2 experimental replicates. ATG3: H929 p = 0.04, *; OPM-2 p = 0.0003, ***; ATG5: H929 p = 0.0009, ***; OPM-2 p = 0.0152, *; ATG7: H929 p = 0.05, *; OPM-2 p = 0.05, *; Student's t test.

G. ATG3, ATG5, and ATG7 mRNA fold changes in U266 CNT cells or U266 cells overexpressing CREB1. Error bars represent the mean ± SEM, n = 2 experimental replicates. ATG3 p = 0.018, *; ATG5 p = 0.10, ns; ATG7 p = 0.0053, **; Student's t test.

H. Western blot analysis for total mTOR, phospho-mTOR, total 4EBP1, phospho-4EBP1, and GAPDH in OPM-2 cells treated with DMSO, CREBi 1 μM, rapamycin (rapa) 25 nM, or CREBi + rapamycin (combo) for 48 hours.

I. AMPKalpha and TSC1 mRNA fold changes in H929 and OPM-2 cells treated with DMSO or CREBi 1 μM for 48 hours. Error bars represent the mean ± SEM, n = 2 experimental replicates. H929: AMPKalpha p = 0.0044, **; TSC1 p = 0.017, *. OPM-2: AMPKalpha p = 0.031, *; TSC1 p = 0.038, *; Student's t test.

J. Western blot analysis for TSC1, phospho-CAMKK2, and GAPDH in H929 and OPM-2 cells treated with DMSO or CREBi 1 μM for 48 hours.

Supplementary Fig. S5. CREB1 regulates proteins involved in the UPR.

A. PERK, EI2FA, IRE1, XBP1, and ATF6 $\log_2$ expression values based on median CREB1 expression in the GSE4452 database. Black dashed lines represent the median value, black dotted lines represent the 25th and 75th percentile, n = 65 patients. PERK p = 0.0026, **; ATF6 p = 0.05, *. All other comparisons are not significant, ns, Student's t test.

B. Correlation between CREB1 and EIF2AK3 (PERK) levels in the database GSE4452. $p < 0.0001$, $R = 0.29$; simple linear regression.

C. Western blot analysis for PERK, phospho-IRE1, IRE1, XBP1, and GAPDH in U266 control (CNT) cells or U266 cells overexpressing CREB1. Quantitative densitometry analysis is presented as the ratio of the proteins of interest to GAPDH. The values obtained for cells overexpressing CREB1 are normalized to those of the control cells. nOD, normalized optical density.

D. Western blot analysis for CREB1, PERK, IRE1, XBP1 (sXBP1 and uXBP1), and GAPDH in OPM-2 control cells (pLKO), or cells silenced for CREB1 (shCREB1). Quantitative densitometry analysis is presented as the ratio of the proteins of interest to GAPDH. The values obtained for cells silenced for CREB1 are normalized to those of the control cells.

E. PERK, IRE1, and total XBP1 mRNA fold changes in OPM-2 control cells (pLKO) or cells silenced for CREB1 (shCREB1). Error bars represent the mean ± SEM, n = 2 experimental replicates. PERK $p = 0.0034$, **; IRE1 $p = 0.95$, ns; total XBP1 $p = 0.01$, *; Student's t test.

F. Quantitative densitometry analysis related to **Figure 3H.**

G. IRE1 mRNA fold changes in OPM-2 cells treated with DMSO or CREBi 1 μM for 2, 4, and 6 hours. Error bars represent the mean ± SEM, n = 3 experimental replicates. DMSO versus 2h $p = 0.0026$, **; DMSO versus 4h $p = 0.09$, ns; DMSO versus 6h $p = 0.08$, ns; Tukey's multiple comparison test.

H. sXBP1/uXBP1 ratio in OPM-2 cells treated with DMSO or CREBi 1 μM for 2, 4, and 6 hours. Error bars represent the mean ± SEM, n = 3 experimental replicates. DMSO versus 2h $p = 0.0074$, **; DMSO versus 4h $p = 0.0211$, *; DMSO versus 6h $p = 0.0053$, **; Tukey's multiple comparison test.

I. ATF4, GADD34, and CHOP mRNA fold changes in OPM-2 cells treated with DMSO or CREBi 1 μM for 24 hours. Error bars represent the mean ± SEM, n = 2 experimental replicates. ATF4 p = 0.014, *; GADD34 p = 0.0014, **; CHOP p = 0.0082, **; Student's t test.

Supplementary Fig. S6. CREB1 inhibition potentiates the effects of proteasome inhibitors.

A. Percentage of dead cells (Zombie Aqua positive) in OPM-2 cells treated with DMSO, 666-15 (CREBi) 0.3 μM, bortezomib (BTZ) 5 nM, or CREBi + BTZ (combo) for 24 hours. Error bars represent the mean ± SEM, n = 2 experimental replicates. CREBi versus CREBi + BTZ p = 0.0003, ***; Tukey's multiple comparison test.

B. Percentage of dead cells (Zombie Aqua positive) in OPM-2 cells treated with DMSO, CREBi 0.3 μM, carfilzomib (CFZ) 12.5 nM, or CREBi + CFZ (combo) for 24 hours. Error bars represent the mean ± SEM, n = 2 experimental replicates. CREBi versus combo p = 0.0008, ***; Tukey's multiple comparison test.

C. GADD34 mRNA fold changes in OPM-2 cells treated with DMSO, CREBi 0.3 μM, BTZ 5 nM, or CREBi + BTZ (combo) for 24 hours. Error bars represent the mean ± SEM, n = 2 experimental replicates. DMSO versus combo p = 0.0087, **; Tukey's multiple comparison test.

D. GADD34 mRNA fold changes in OPM-2 cells treated with DMSO, CREBi 0.3 μM, CFZ 5 nM, or CREBi + CFZ (combo) for 24 hours. Error bars represent the mean ± SEM, n = 2 experimental replicates. DMSO versus CFZ p = 0.015, *; DMSO versus combo p = 0.0028, **; CREBi versus combo p = 0.0096, **; Tukey's multiple comparison test.

E. CHOP mRNA fold changes in OPM-2 and H929 treated with DMSO, CREBi 0.3 μM, BTZ 5 nM, or CREBi + BTZ (combo) for 24 hours. Error bars represent the mean ± SEM, n = 2 experimental replicates. OPM-2: CREBi versus BTZ p = 0.01, *; CREBi versus combo p =

0.0031, **. H929: BTZ versus combo $p = 0.0128$, *; CREBi versus combo $p = 0.01$, *; Tukey's multiple comparison test.

F. CHOP mRNA fold changes in OPM-2 and H929 cells treated with DMSO, CREBi 0.3 μM, CFZ 5 nM, or CREBi + CFZ (combo) for 24 hours. Error bars represent the mean ± SEM, n = 2 experimental replicates. OPM-2: CREBi versus combo $p = 0.0056$, **; H929: CREBi versus combo $p = 0.0413$, *; Tukey's multiple comparison test.

G. Normalized proteasome activity in OPM-2 cells treated for 24 hours with DMSO or CREBi 0.3 μM and 1 μM. Error bars represent the mean ± SEM, n = 2 experimental replicates. DMSO versus CREBi 0.3 μM $p = 0.06$, ns; DMSO versus CREBi 1 μM $p = 0.01$, *; Student's t test.

Supplementary Fig. S7. TXNIP expression and regulation in B cells and MM cells.

A. TXNIP RNA-sequencing expression values in naïve B cells (n = 4) and plasmablasts (n = 4) from healthy donors. Dashed lines represent the median value; dotted lines represent the 25th and 75th percentile. Data from Monaco et al. $p < 0.0001$, ****; Student's t test.

B. TXNIP RNA-sequencing expression values in naïve B cells (n = 9), germinal center (GC) B cells (n = 8), and normal plasma cells (nPCs) (n = 10) from healthy donors in our dataset. Solid lines represent the median value. Naïve B cells versus nPCs $p = 0.0006$, ***; Student's t test.

C. Western blot analysis for TXNIP and GAPDH in a panel of MM cell lines

D. Immunoprecipitation (IP) for TXNIP in OPM-2 cells and blot (IB) for TRX. Line 1 is total lysates (input), line 2 is IgG rabbit alone, and line 3 is TXNIP-IP (immunoprecipitate).

E. Western blot analysis for TXNIP and GAPDH in H929 and OPM-2 cells treated with DMSO or tunicamycin (Tuni) 1 μM for 24 hours.

Supplementary Fig. S8. TXNIP silencing induces cellular death and modulates genes involved in the UPR.

A. TXNIP mRNA fold changes in H929 and OPM-2 control cells (pLKO), or cells silenced for TXNIP (shTXNIP). Error bars represent the mean ± SEM, n = 2 experimental replicates. H929 $p = 0.008$, **; OPM-2 $p = 0.01$, *; Student's t test.

B. Cellular growth in OPM-2 control cells (pLKO), or cells silenced for TXNIP (shTXNIP). Error bars represent the mean ± SD, n = 2 experimental replicates. 48 hours $p = 0.0004$, ***; 72 hours $p < 0.0001$, ****; Student's t test.

C. Percentage of viable cells in OPM-2 and H929 control cells (pLKO), or cells silenced for TXNIP (shTXNIP) at 72 hours. Viability is assessed by MTT assay and percentage is normalized to the control cells. Error bars represent the mean ± SEM, n = 2 experimental replicates. OPM-2 $p = 0.0092$, **; H929 $p = 0.0338$, *; Student's t test.

D. Percentage of dead cells (Annexin V^+, PI^+) in OPM-2 control cells (pLKO), or cells silenced for TXNIP (shTXNIP). Error bars represent the mean ± SEM, n = 2 experimental replicates. $p = 0.05$, *; Student's t test.

E. Gene signature enrichment analysis in H929 cells silenced for TXNIP versus H929 control cells (pLKO). Normalized Enrichment Score (NES) values are plotted. False Discovery Rate <25% was used as threshold for enrichment. n = 3 experimental replicates.

F. Volcano plot of UPR-related genes. In red are highlighted the top significantly upregulated genes, in blue the top significantly downregulated genes. n = 3 experimental replicates.

G. PERK, IRE1, and total XBP1 mRNA fold changes in H929 and OPM-2 control cells (pLKO), or cells silenced for TXNIP (shTXNIP). Error bars represent the mean ± SEM, n = 2 experimental replicates. PERK: H929 $p = 0.014$, *; OPM-2 $p = 0.032$, *; IRE1: H929 $p = 0.04$, *; OPM-2 $p = 0.01$, *; Total XBP1: H929 $p = 0.004$, **; OPM-2 $p = 0.01$, *; Student's t test.

H. CHOP mRNA fold changes in H929 control cells (pLKO) or cells silenced for TXNIP (shTXNIP). Error bars represent the mean ± SEM, n = 2 experimental replicates. *p* = 0.02, *; Student's t test.

Supplementary Fig. S9. TXNIP-X-IN has minimal activity in MM cells.

A. TXNIP mRNA fold changes in OPM-2 cells treated with DMSO or SRI-37330 0.5, 1, 3, and 5 μM for 48 hours. Error bars represent the mean ± SEM, n = 2 experimental replicates. DMSO versus SRI-37330 0.5 μM *p* = 0.018, *; DMSO versus SRI-37330 1 μM *p* = 0.0027, **; DMSO versus SRI-37330 3 μM *p* = 0.0006, ***; DMSO versus SRI-37330 5 μM *p* = 0.0004, ***; Tukey's multiple comparison test.

B. Western blot analysis for TXNIP and GAPDH in H929 and OPM-2 cells treated with DMSO or TXNIP-IN-1 5 μM for 48 hours.

C. TXNIP mRNA fold changes in OPM-2 cells treated with DMSO or TXNIP-IN-1 5 μM for 72 hours. Error bars represent the mean ± SEM, n = 2 experimental replicates. *p* = 0.22, ns, not significant; Student's t test.

D. Percentage of viable cells in H929, OPM-2, and RPMI-8226 cells treated with DMSO or increasing doses of TXNIP-IN for 72 hours. Viability is assessed by MTT assay and percentage is normalized to the control cells. n = 2 experimental replicates.

Supplementary Fig. S10. SRI-37330, a TXNIP inhibitor, is toxic against MM cells.

A. OPM-2 cells treated with DMSO, SRI-37330 0, 1, 2, or 3 μM, 666–15 010 μM, and all their combinations. Values represent the synergy scores calculated using the mean of n =2 experimental replicates.

B. ATG3, ATG5, and ATG7 mRNA fold changes in OPM-2 cells treated with DMSO or SRI-37330 3 μM for 48 hours. Error bars represent the mean ± SEM, n = 3 experimental replicates. ATG3 $p = 0.0013$, **; ATG5 $p = 0.03$, *; ATG7 $p = 0.003$, **; Student's t test.

C. Western blot analysis for ULK1, ATG5, ATG7, LC3, and GAPDH in OPM-2 cells treated with DMSO or SRI-37330 3 and 5 μM for 48 hours.

D. Quantitative densitometry analysis for panel **C** is presented as the ratio of the proteins of interest to GAPDH. The values obtained for cells treated with SRI-37330 are normalized to those of the DMSO-treated cells. nOD, normalized optical density.

E. Western blot analysis for total mTOR, phospho-MTOR, total 4EBP1, phospho-4EBP1, and GAPDH in OPM-2 cells treated with DMSO or SRI-37330 1 and 3 μM for 48 hours.

F. Quantitative densitometry analysis for **figure 5J** is presented as the ratio of the proteins of interest to GAPDH. The values obtained for cells treated with SRI-37330 are normalized to those of the DMSO-treated cells.

G. IRE1, sXBP1/uXBP1 ratio, total XBP1, and PERK mRNA fold changes in OPM-2 cells treated with DMSO or SRI-37330 3 μM for 48 hours. Error bars represent the mean ± SEM, n = 2 experimental replicates. IRE1 $p = 0.0007$, ***; sXBP1/uXBP1 ratio $p = 0.16$, ns; total XBP1 $p = 0.03$, *; PERK $p = 0.05$, *; Student's t test.
