# Supplementary material for: Regulation of stress tolerance by CREB1 sustains multiple myeloma cell survival
Source: Cell Death Dis. 2026 Jan 16;17(1):46. doi: 10.1038/s41419-025-08246-z (PMC12811356; doi:10.1038/s41419-025-08246-z)

Figure 1E

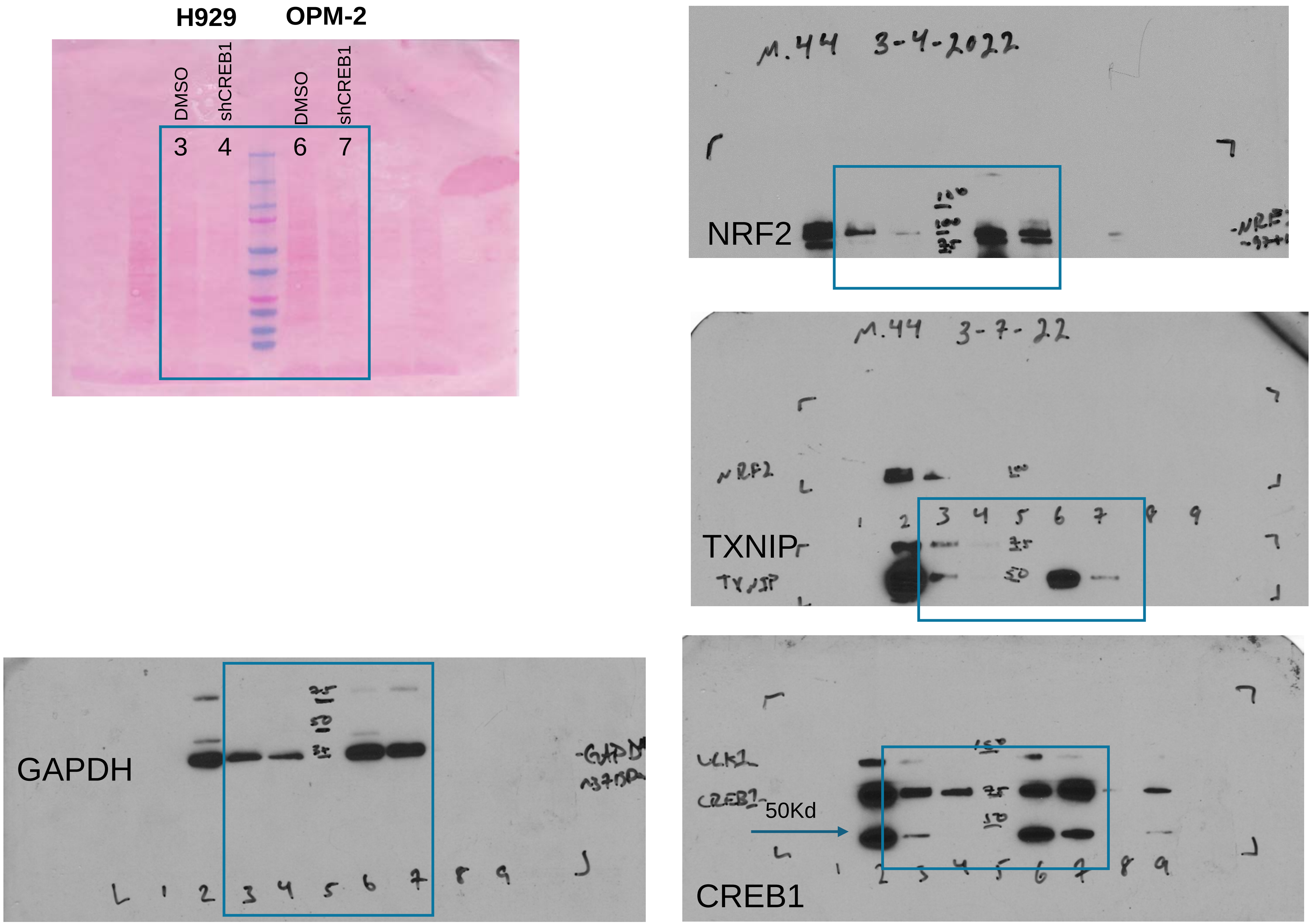

H929
OPM-2
DMSO
shCREB1
DMSO
shCREB1
3
4
6
7
M.44 3-4-2022
NRF2
M.44 3-7-22
TXNIP
GAPDH
CREB1
50Kd

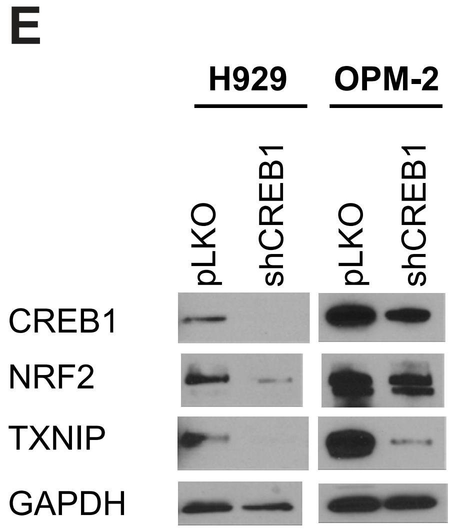

E
H929
OPM-2
pLKO
shCREB1
pLKO
shCREB1
CREB1
NRF2
TXNIP
GAPDH

OPM2

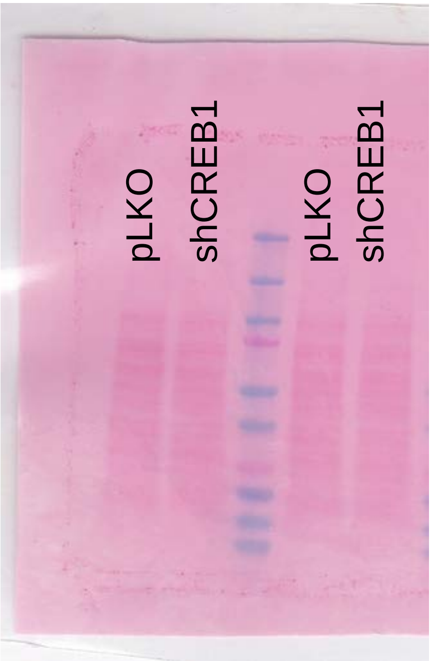

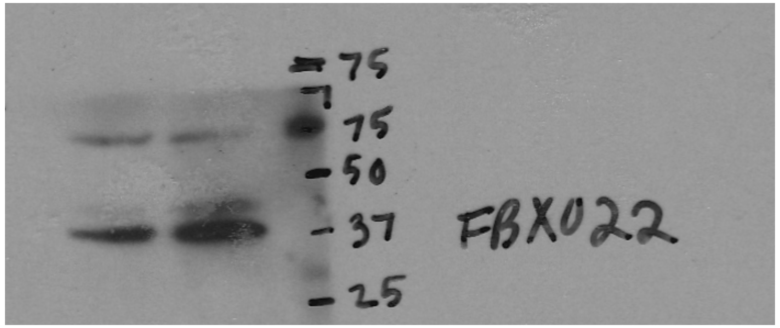

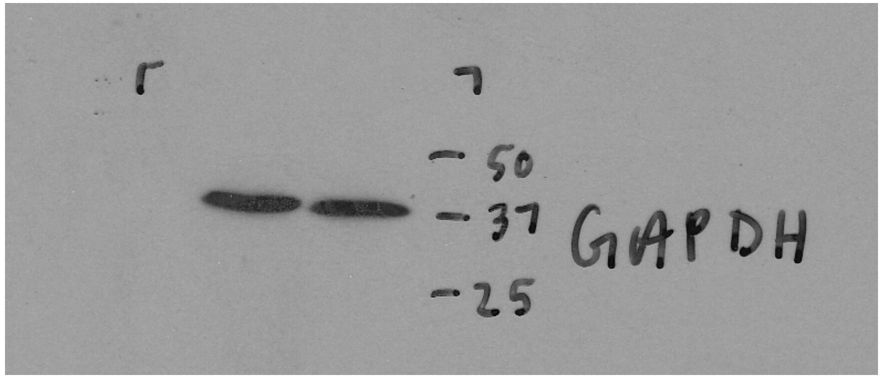

Figure S3B

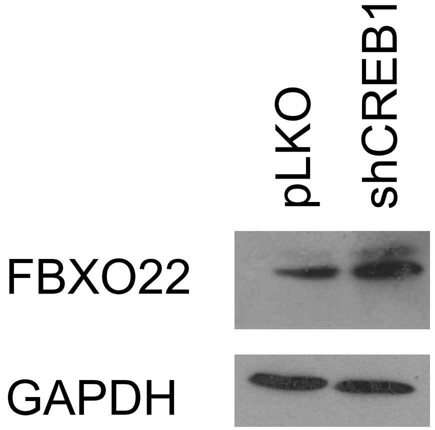

Figure 1G- H929

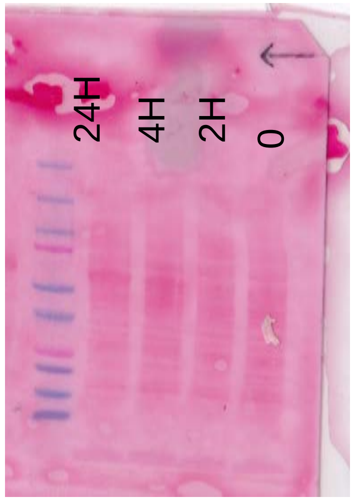

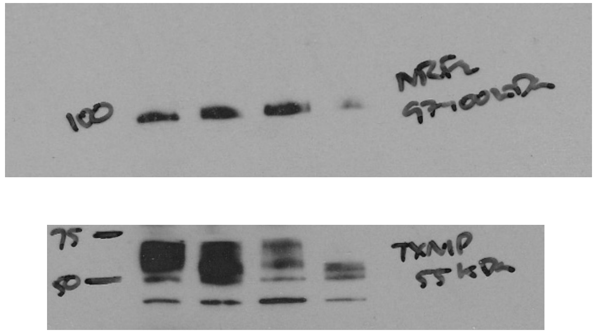

BACH1

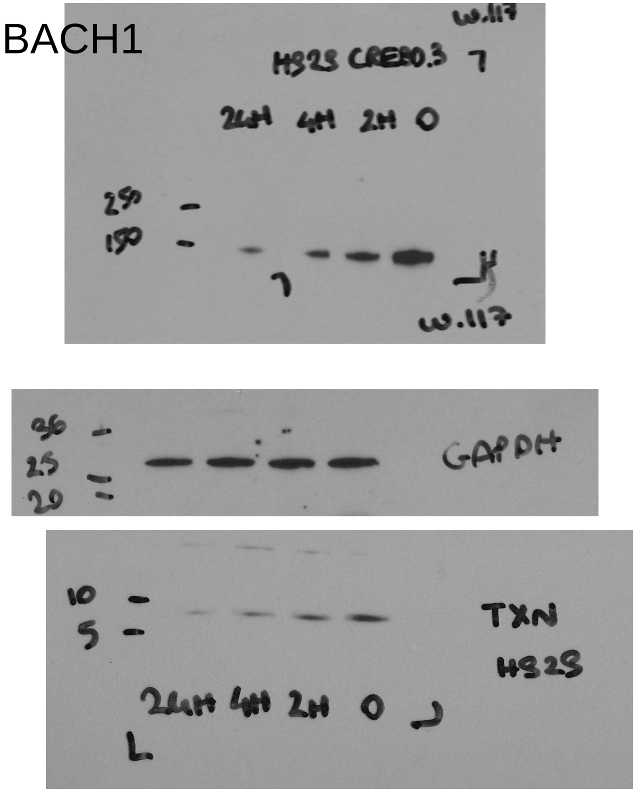

Figure 1G- OPM2

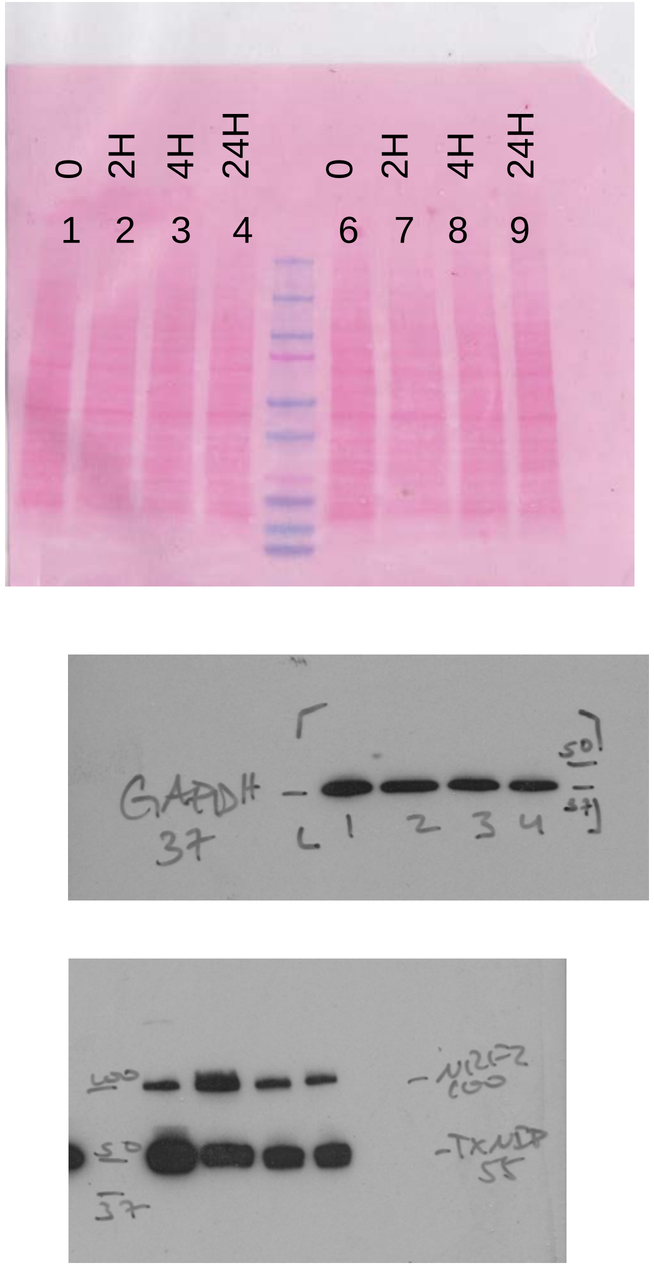

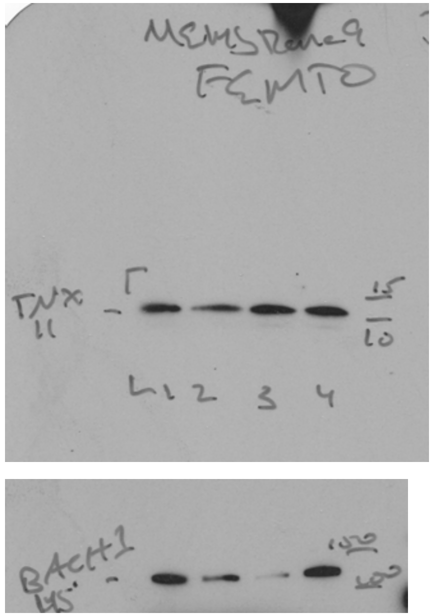

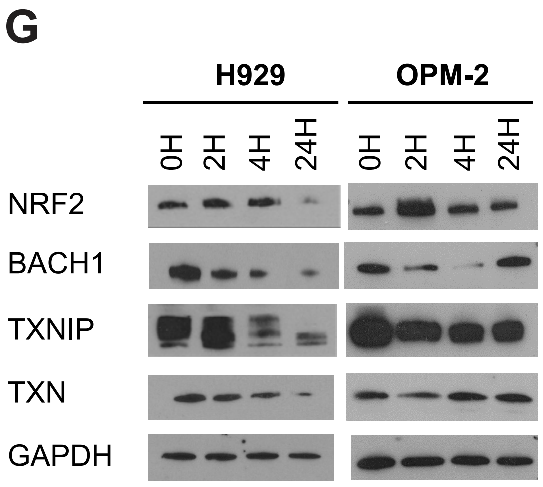

Figure S3C

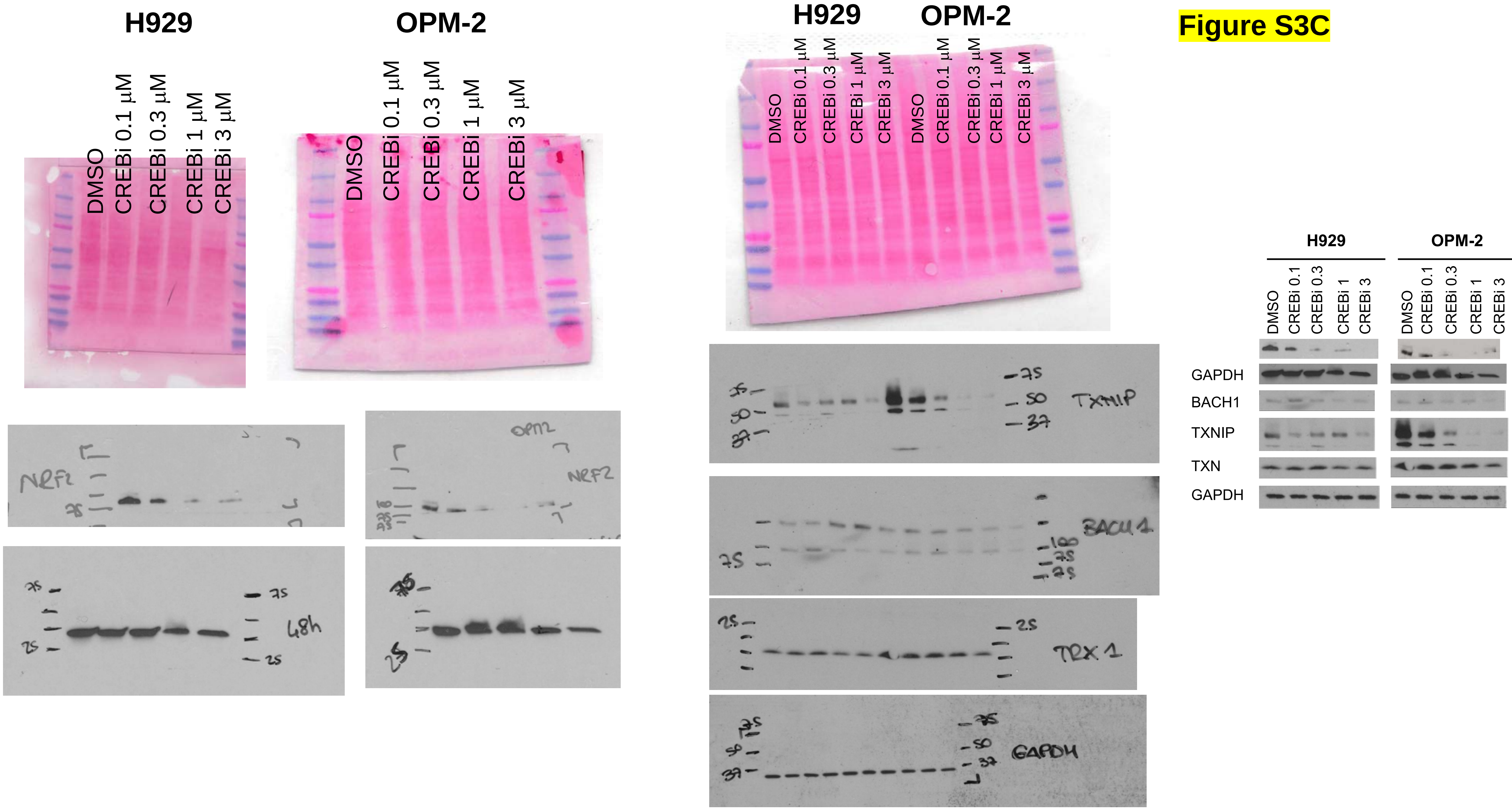
H929
OPM-2
DMSO
CREBi 0.1 µM
CREBi 0.3 µM
CREBi 1 µM
CREBi 3 µM
DMSO
CREBi 0.1
CREBi 0.3
CREBi 1
CREBi 3
GAPDH
BACH1
TXNIP
TXN
GAPDH

Figure 1H

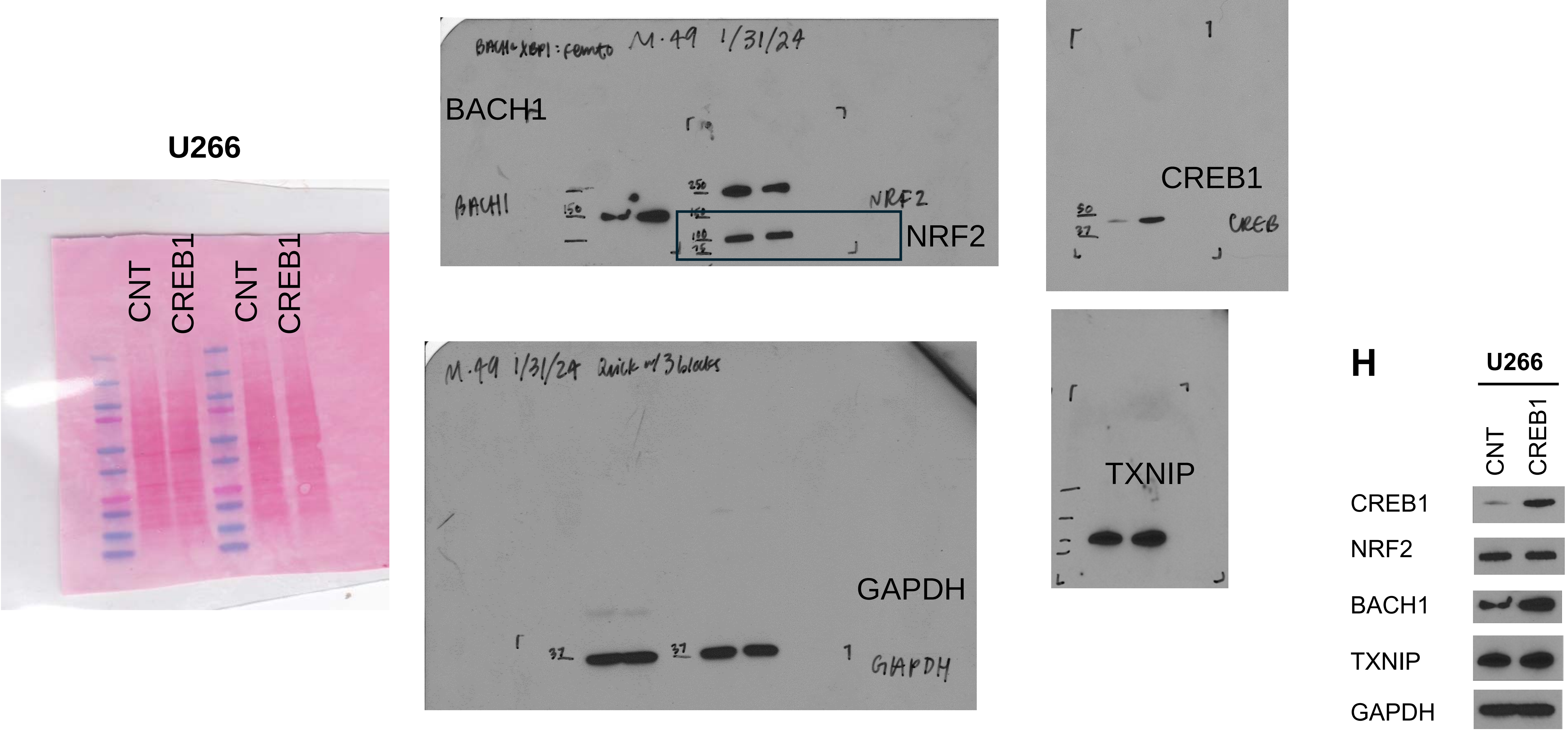
U266
CNT
CREB1
CNT
CREB1
BACH1
NRF2
CREB1
TXNIP
GAPDH
H
U266
CNT
CREB1
CREB1
NRF2
BACH1
TXNIP
GAPDH

Figure S3E

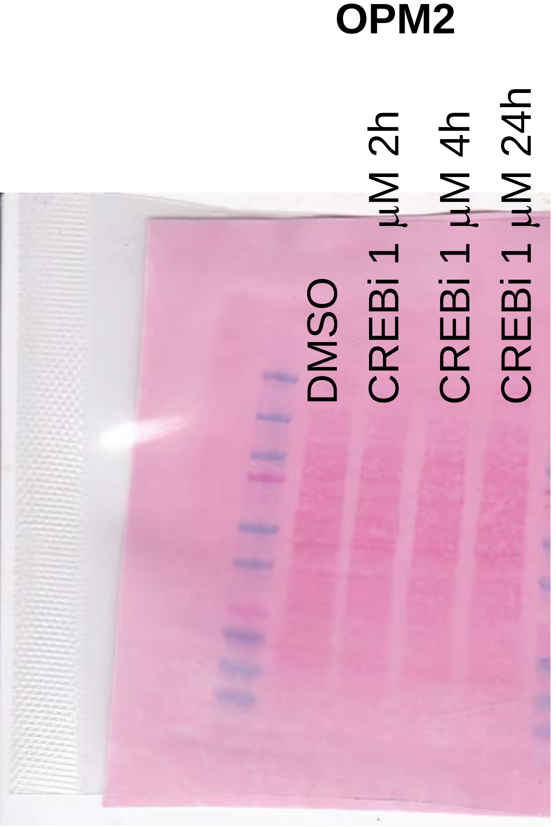
OPM2
DMSO
CREBi 1 μM 2h
CREBi 1 μM 4h
CREBi 1 μM 24h

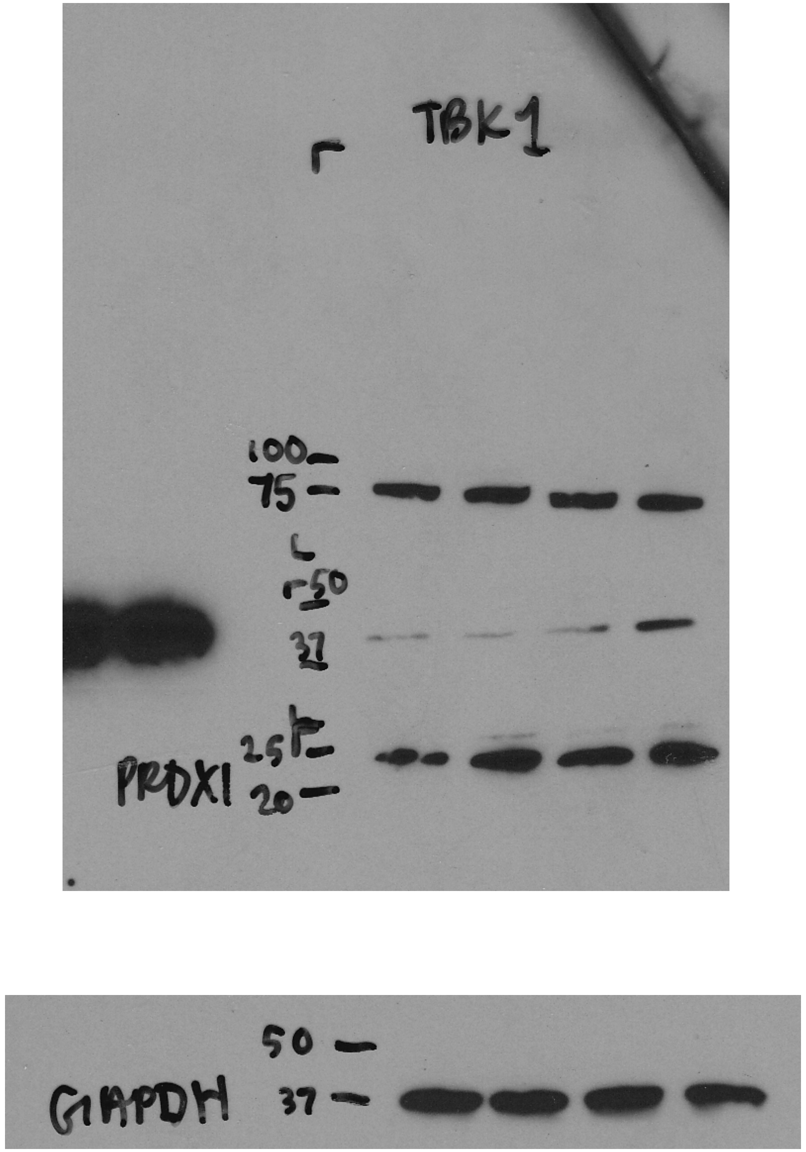
TBK1
100
75
50
37
25
PRDX1
20
GAPDH
50
37

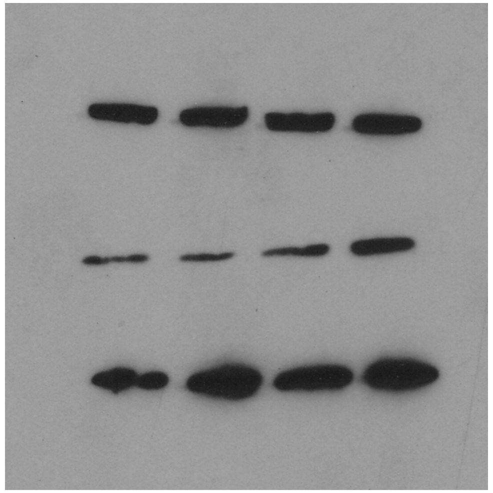
FBXO22

E

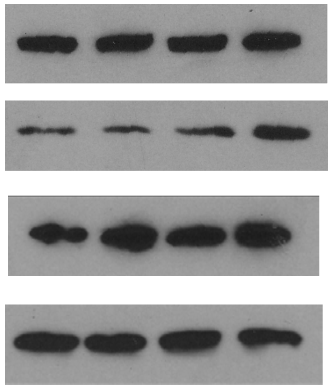
OPM-2
0H
2H
4H
24H
TBK1
FBXO22
PRDX1
GAPDH

Figure 2B

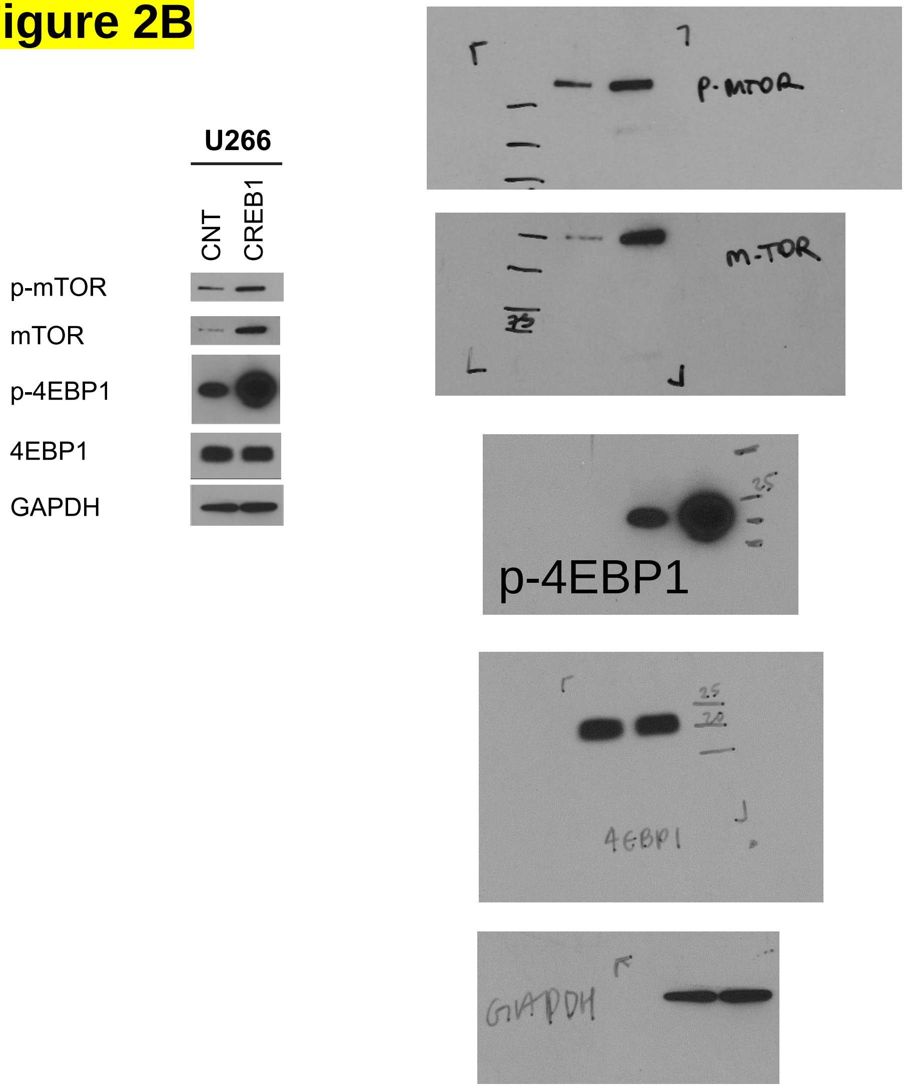

Figure S4A

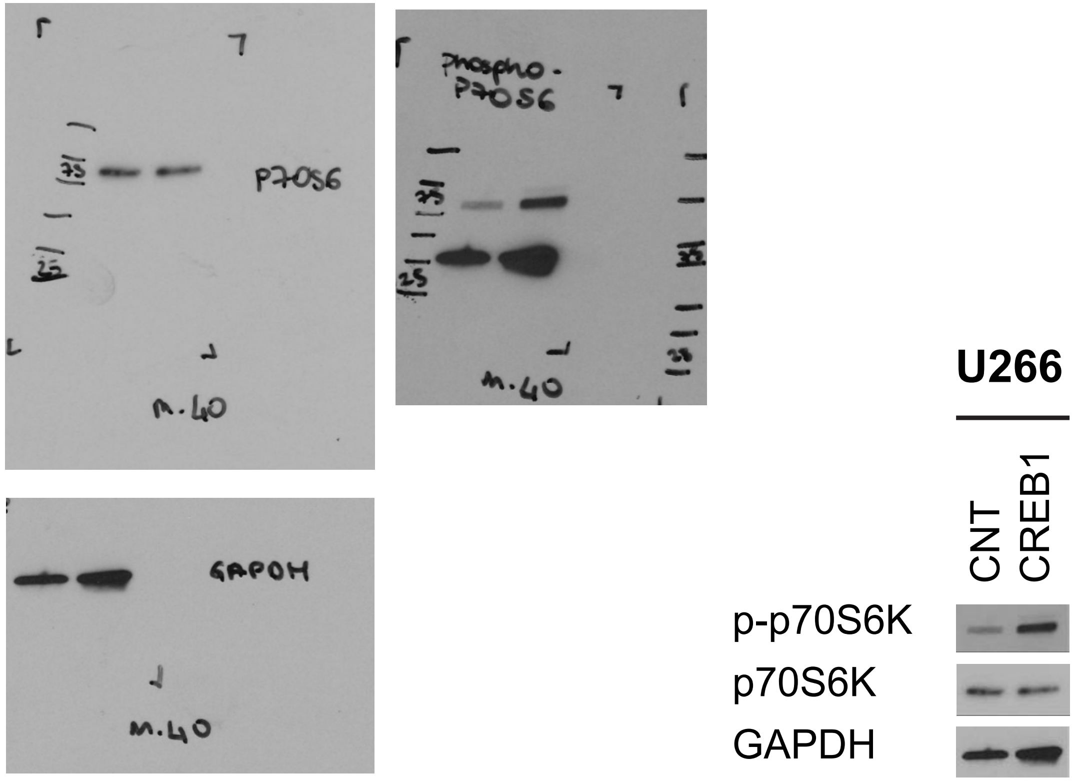

Figure 2C

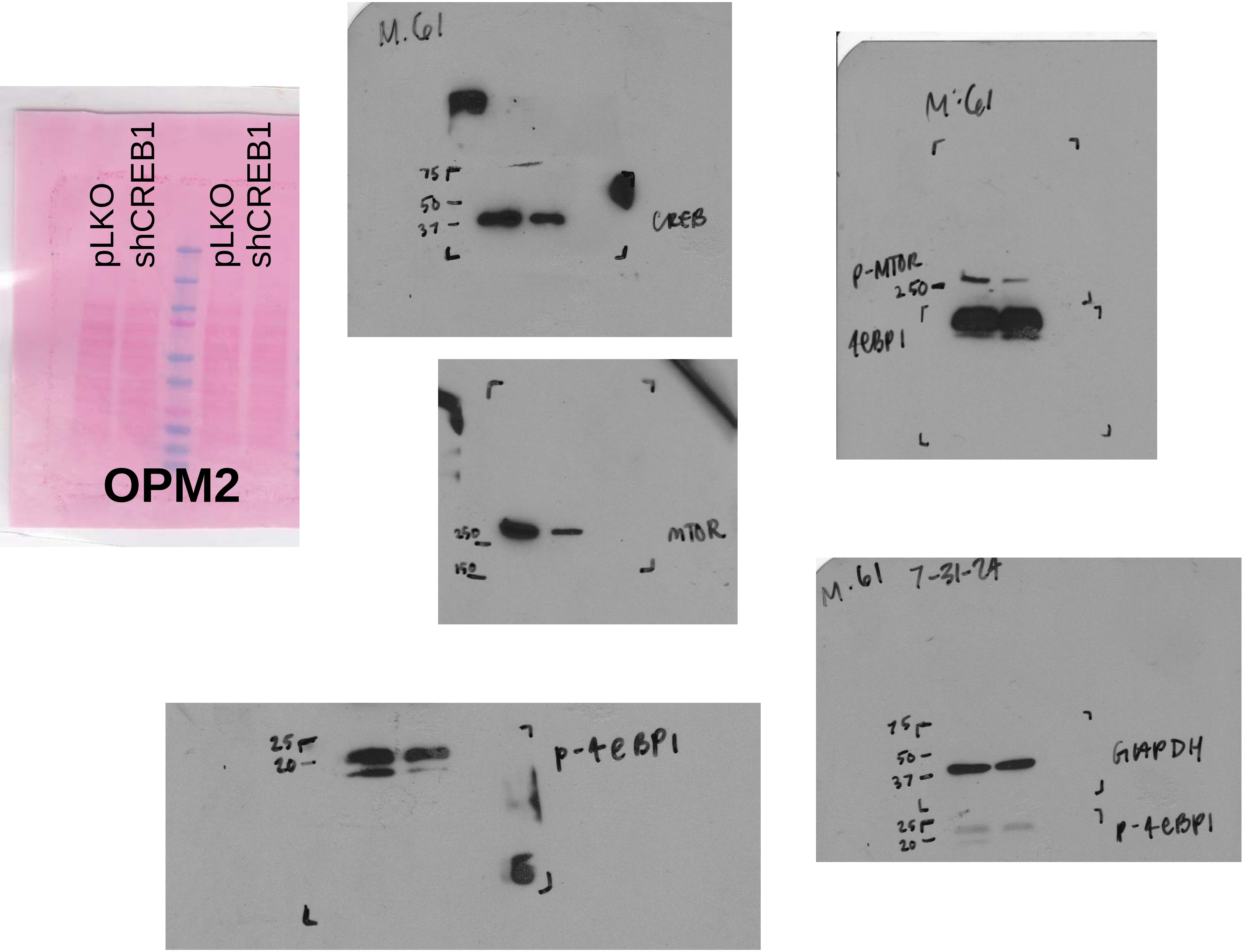
pLKO
shCREB1
pLKO
shCREB1
OPM2
M.61
75
50
37
CREB
M.61
p-MTOR
250
4EBP1
250
150
mTOR
25
20
p-4EBP1
M.61 7-31-24
75
50
37
GAPDH
25
20
p-4EBP1

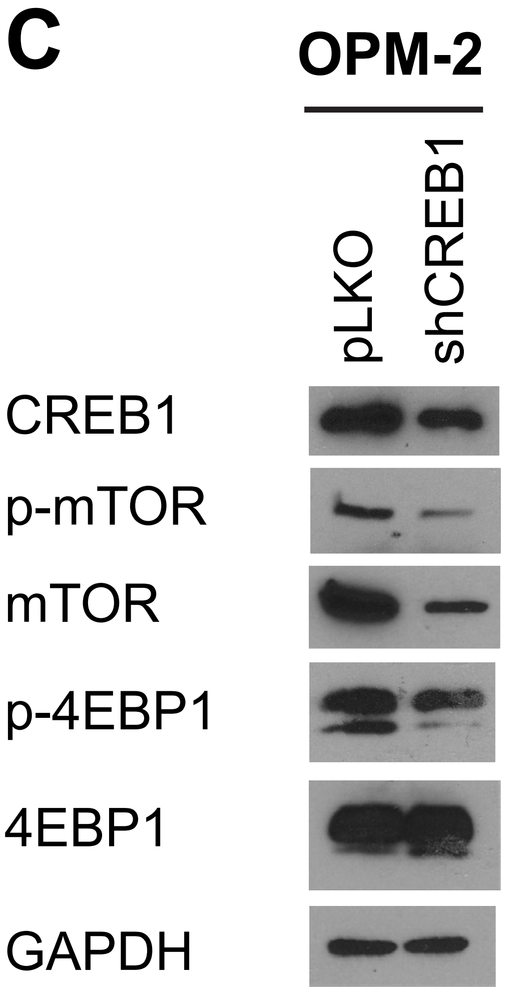
C
OPM-2
pLKO
shCREB1
CREB1
p-mTOR
mTOR
p-4EBP1
4EBP1
GAPDH

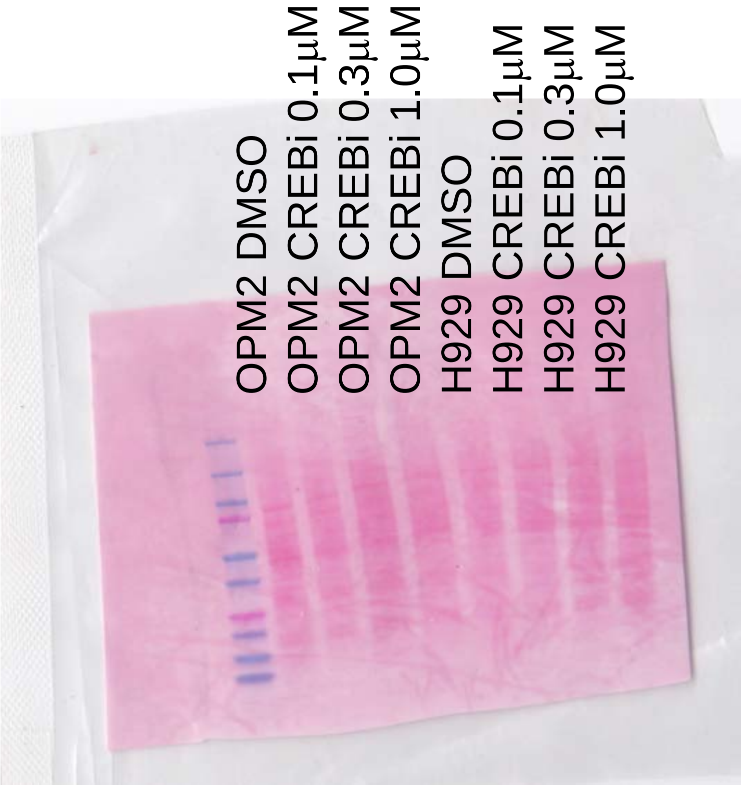

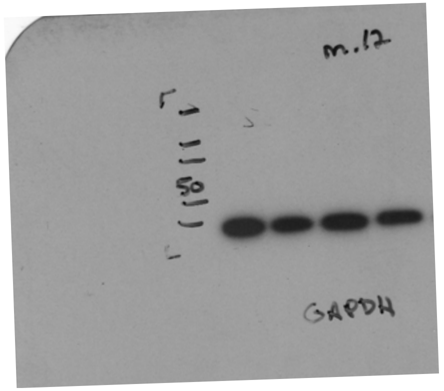

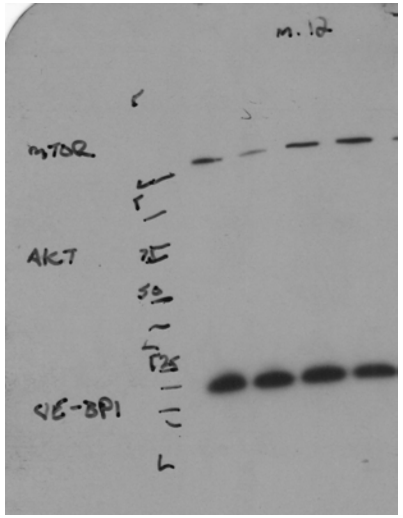

Figure 2D

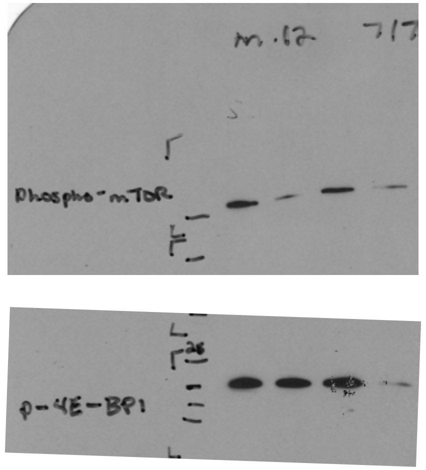

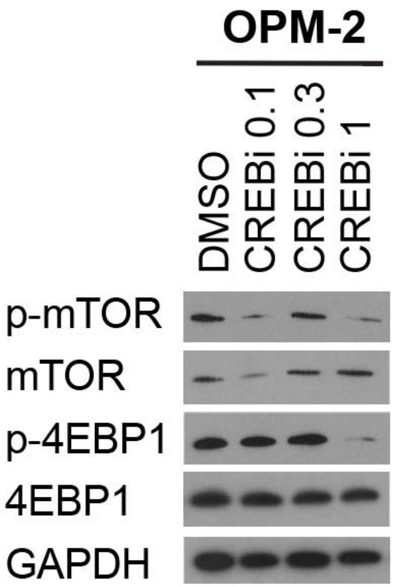

Image shows only OPM-2

Figure S4C

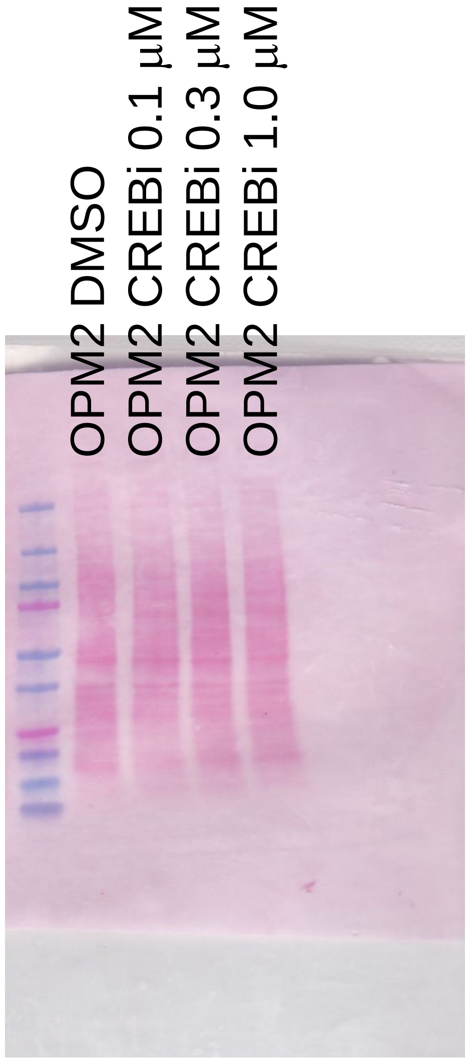
OPM2 DMSO
OPM2 CREBi 0.1 µM
OPM2 CREBi 0.3 µM
OPM2 CREBi 1.0 µM

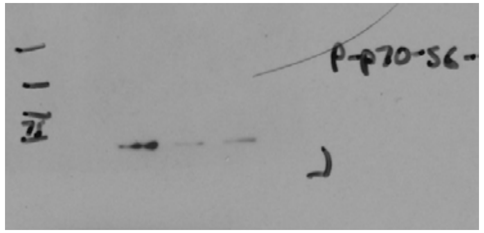
P-p70-S6-

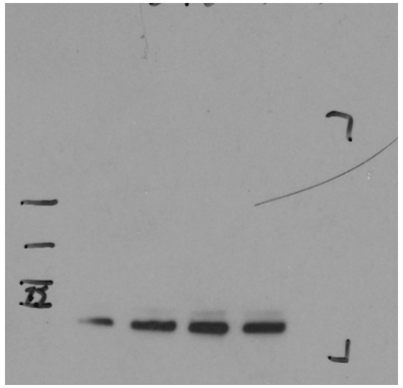

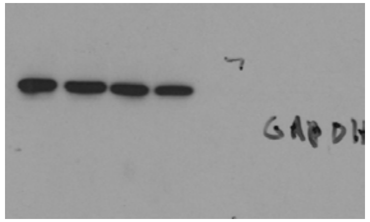
GAPDH

C

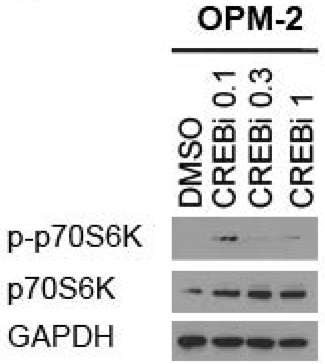
OPM-2
DMSO
CREBi 0.1
CREBi 0.3
CREBi 1
p-p70S6K
p70S6K
GAPDH

Figure 2H

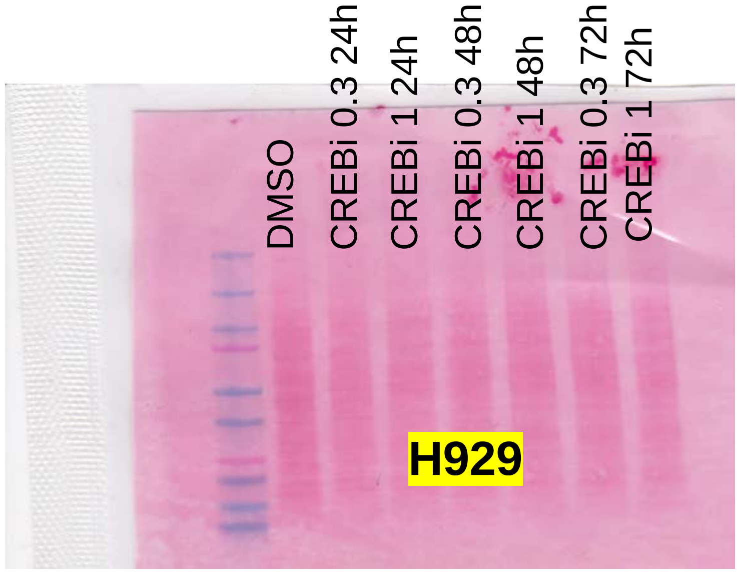
DMSO
CREBi 0.3 24h
CREBi 1 24h
CREBi 0.3 48h
CREBi 1 48h
CREBi 0.3 72h
CREBi 1 72h
H929

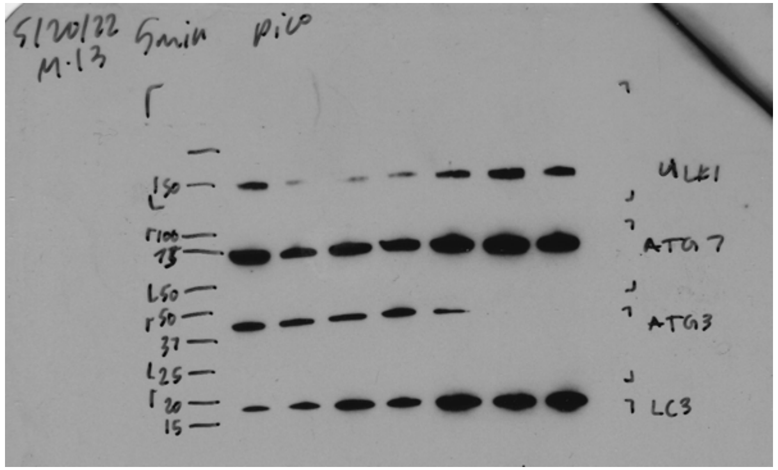
5/20/22 5min pico
M.13
150
100
75
150
50
37
25
20
15
ULK1
ATG7
ATG3
LC3

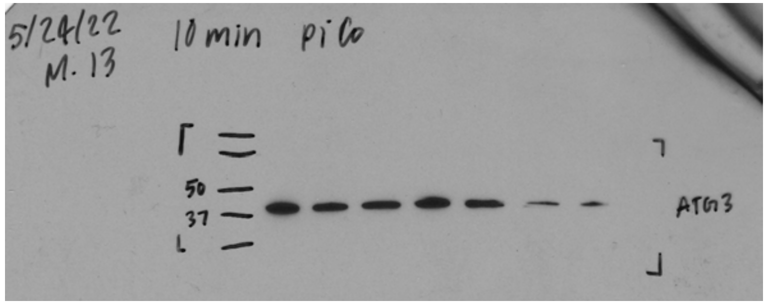
5/24/22 10 min pico
M. 13
50
37
ATG3

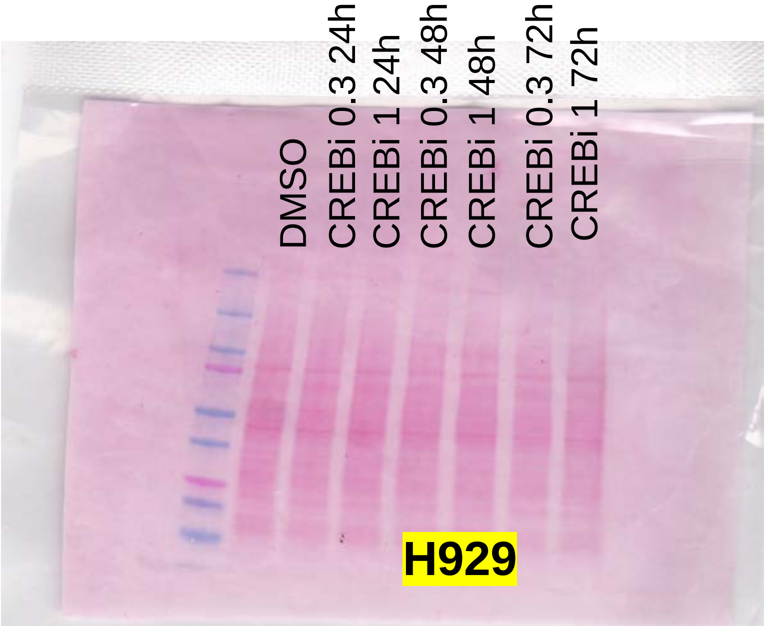
DMSO
CREBi 0.3 24h
CREBi 1 24h
CREBi 0.3 48h
CREBi 1 48h
CREBi 0.3 72h
CREBi 1 72h
H929

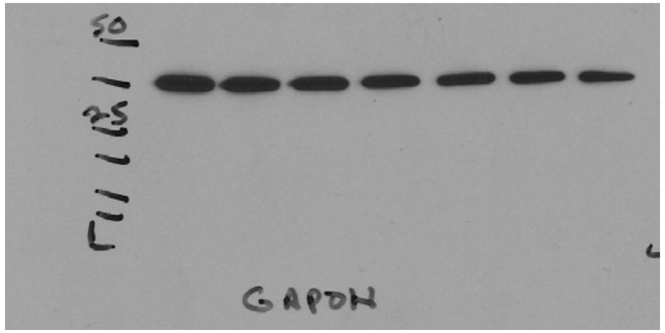
50
25
GAPDH

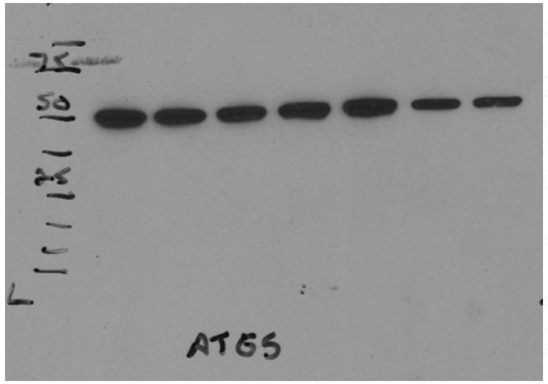
75
50
25
ATG5

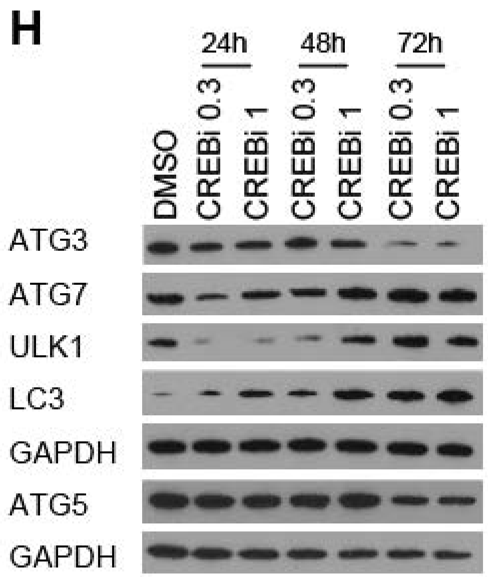
H
24h
48h
72h
DMSO
CREBi 0.3
CREBi 1
CREBi 0.3
CREBi 1
CREBi 0.3
CREBi 1
ATG3
ATG7
ULK1
LC3
GAPDH
ATG5
GAPDH

Figure S4D

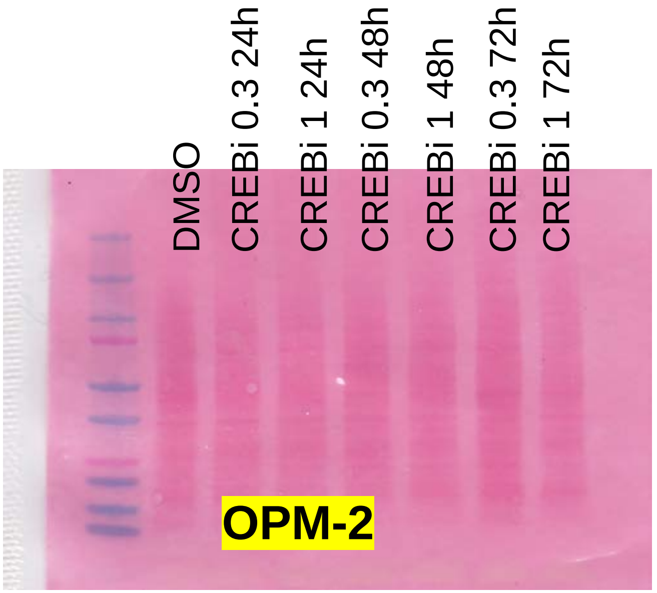

DMSO
CREBi 0.3 24h
CREBi 1 24h
CREBi 0.3 48h
CREBi 1 48h
CREBi 0.3 72h
CREBi 1 72h
OPM-2

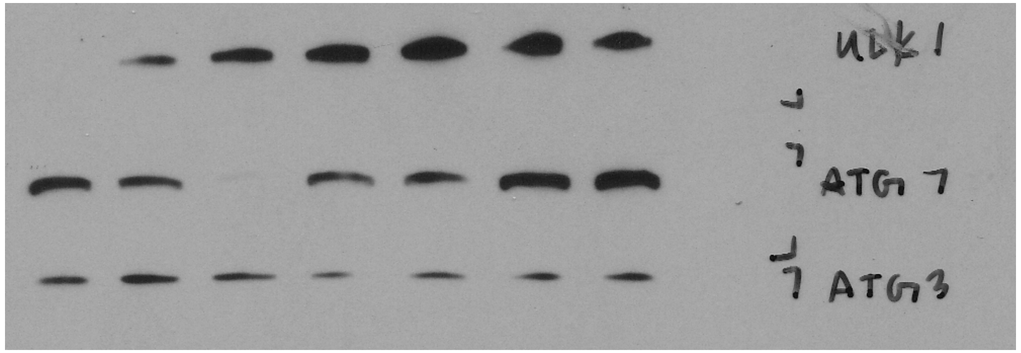

ULK1
ATG7
ATG3

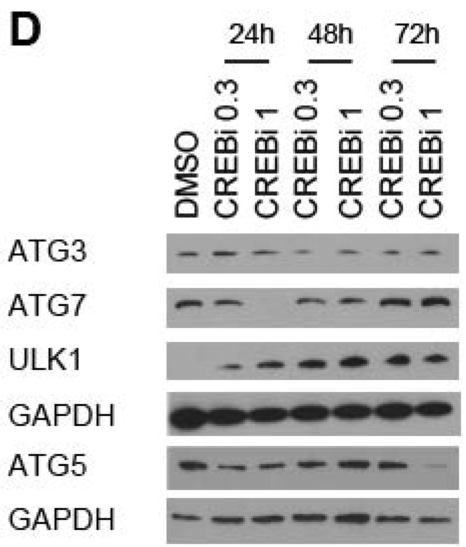

D
24h
48h
72h
DMSO
CREBi 0.3
CREBi 1
CREBi 0.3
CREBi 1
CREBi 0.3
CREBi 1
ATG3
ATG7
ULK1
GAPDH
ATG5
GAPDH

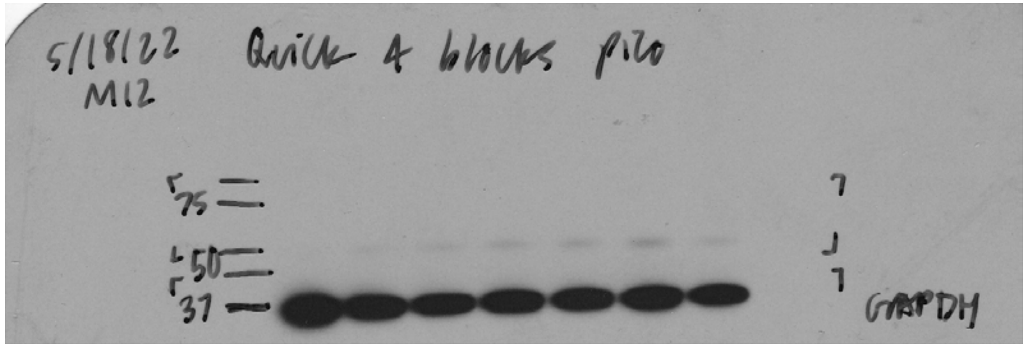

5/18/22
M12
Quick 4 blocks pico
75
50
37
GAPDH

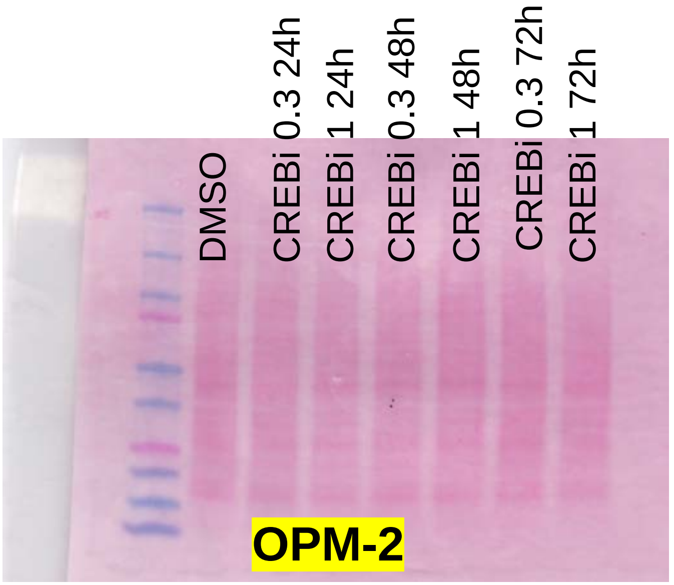

DMSO
CREBi 0.3 24h
CREBi 1 24h
CREBi 0.3 48h
CREBi 1 48h
CREBi 0.3 72h
CREBi 1 72h
OPM-2

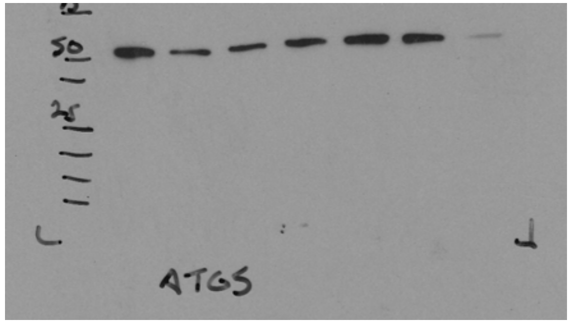

50
25
ATG5

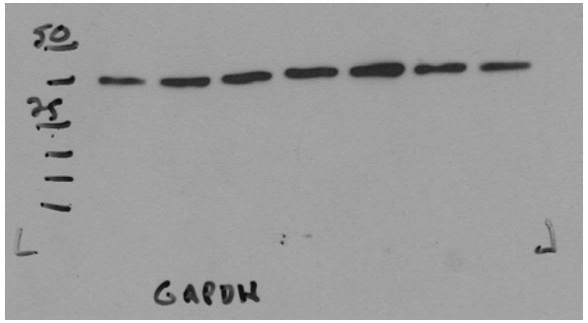

50
25
GAPDH

Figure 2I

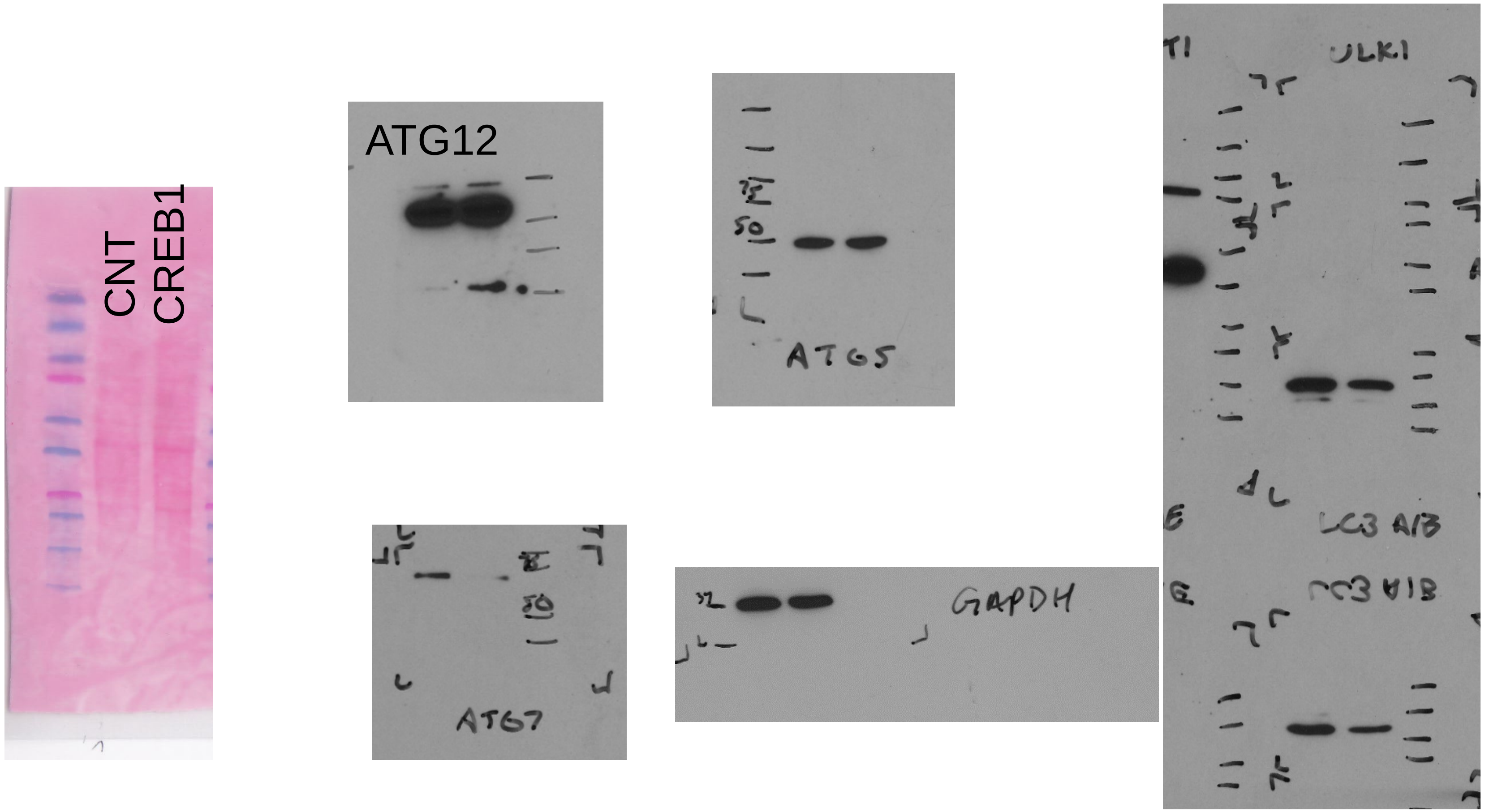

CNT
CREB1
ATG12
ATG5
ATG7
GAPDH
ULK1
LC3 A/B

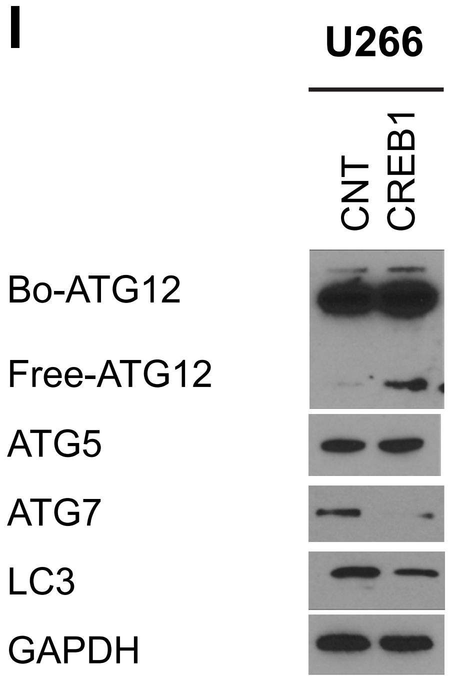

I
U266
CNT
CREB1
Bo-ATG12
Free-ATG12
ATG5
ATG7
LC3
GAPDH

Figure 2K

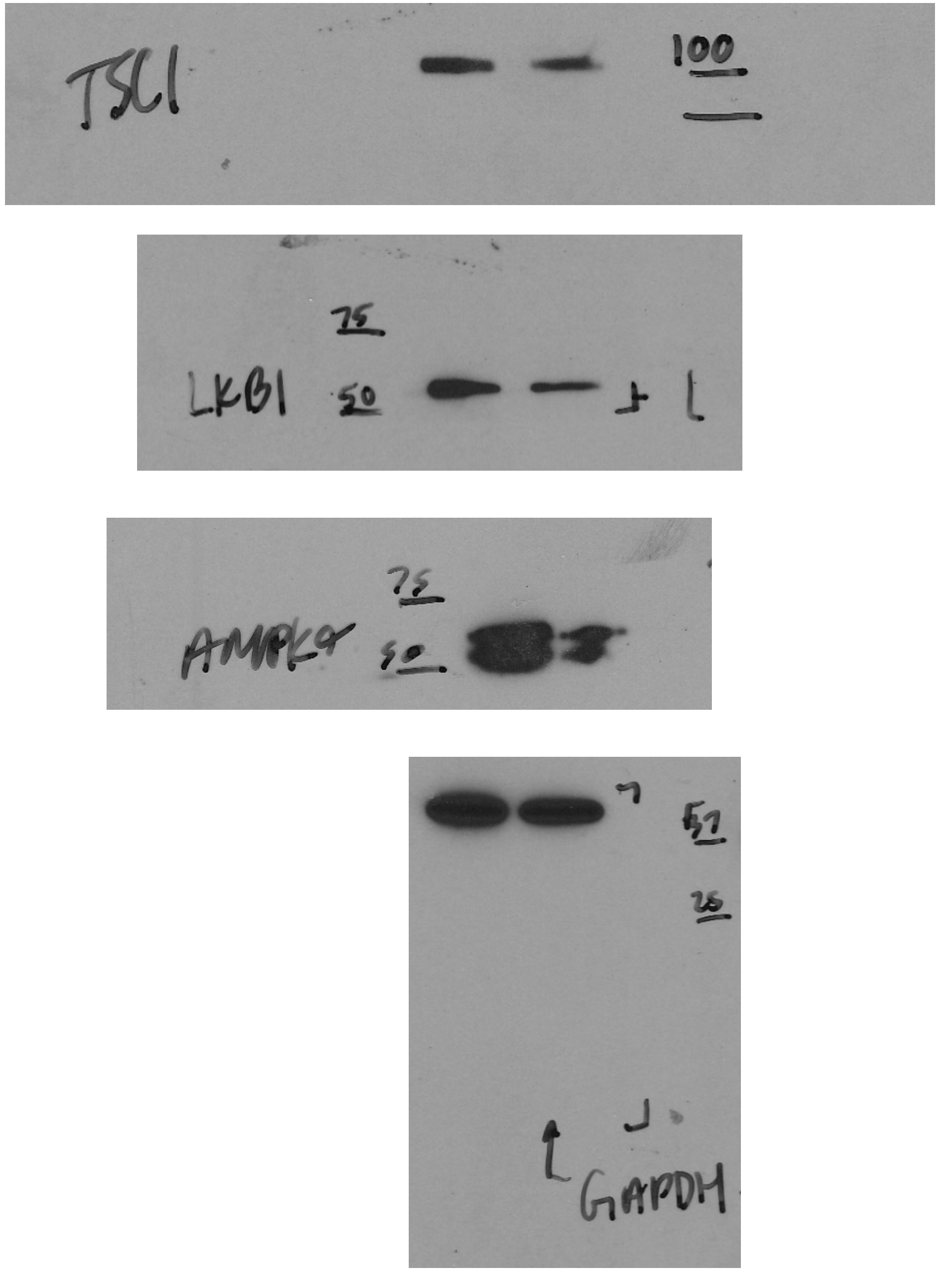

U266

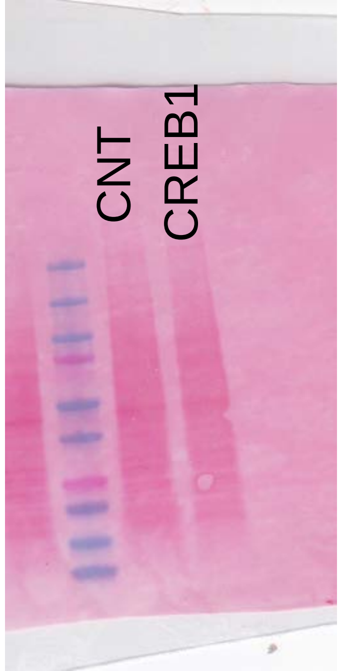

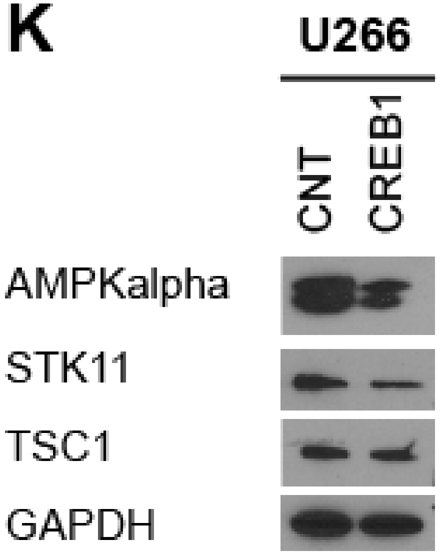

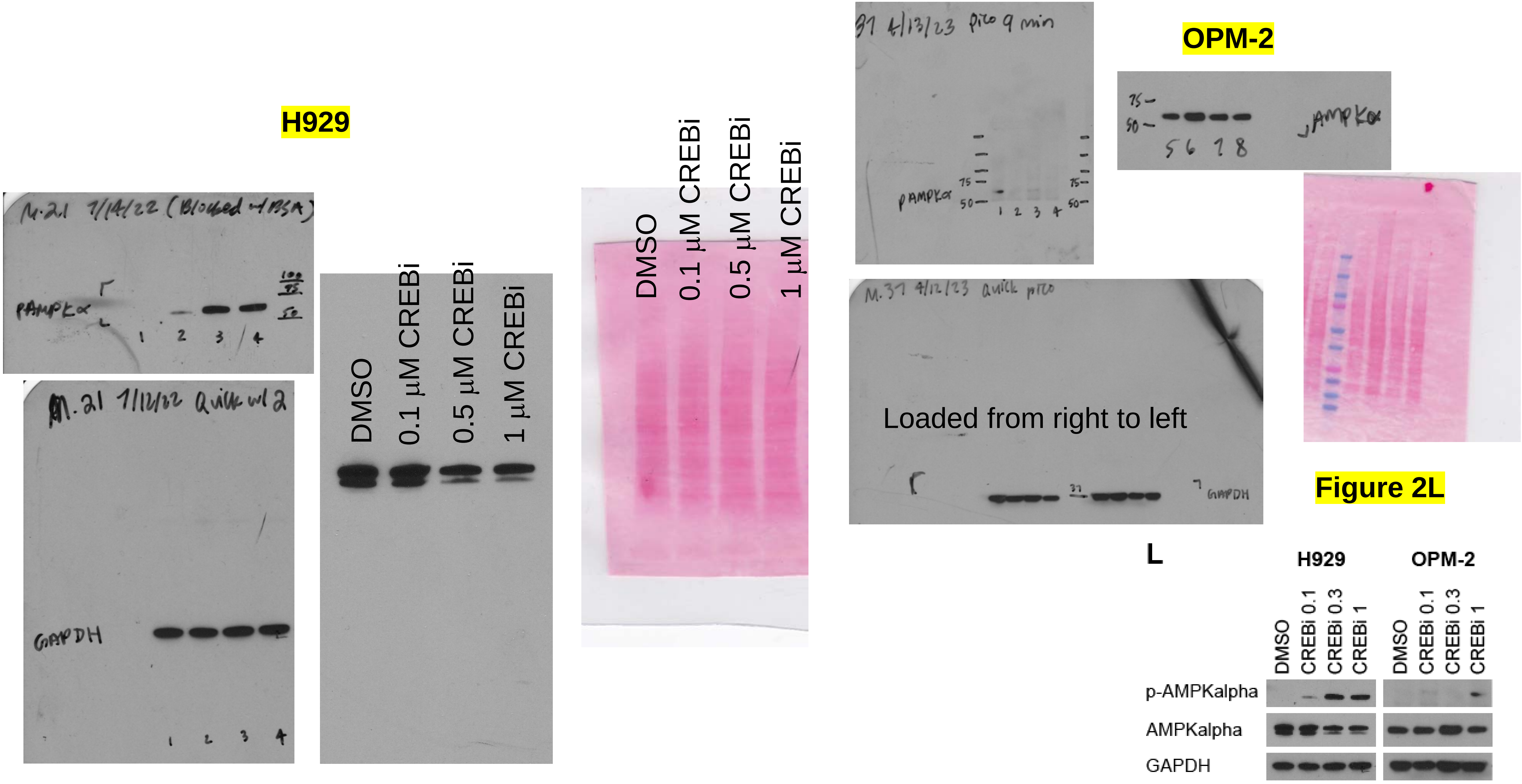

H929
OPM-2
DMSO
0.1 µM CREBi
0.5 µM CREBi
1 µM CREBi
DMSO
0.1 µM CREBi
0.5 µM CREBi
1 µM CREBi
Loaded from right to left
Figure 2L
L
H929
OPM-2
DMSO
CREBi 0.1
CREBi 0.3
CREBi 1
DMSO
CREBi 0.1
CREBi 0.3
CREBi 1
p-AMPKalpha
AMPKalpha
GAPDH

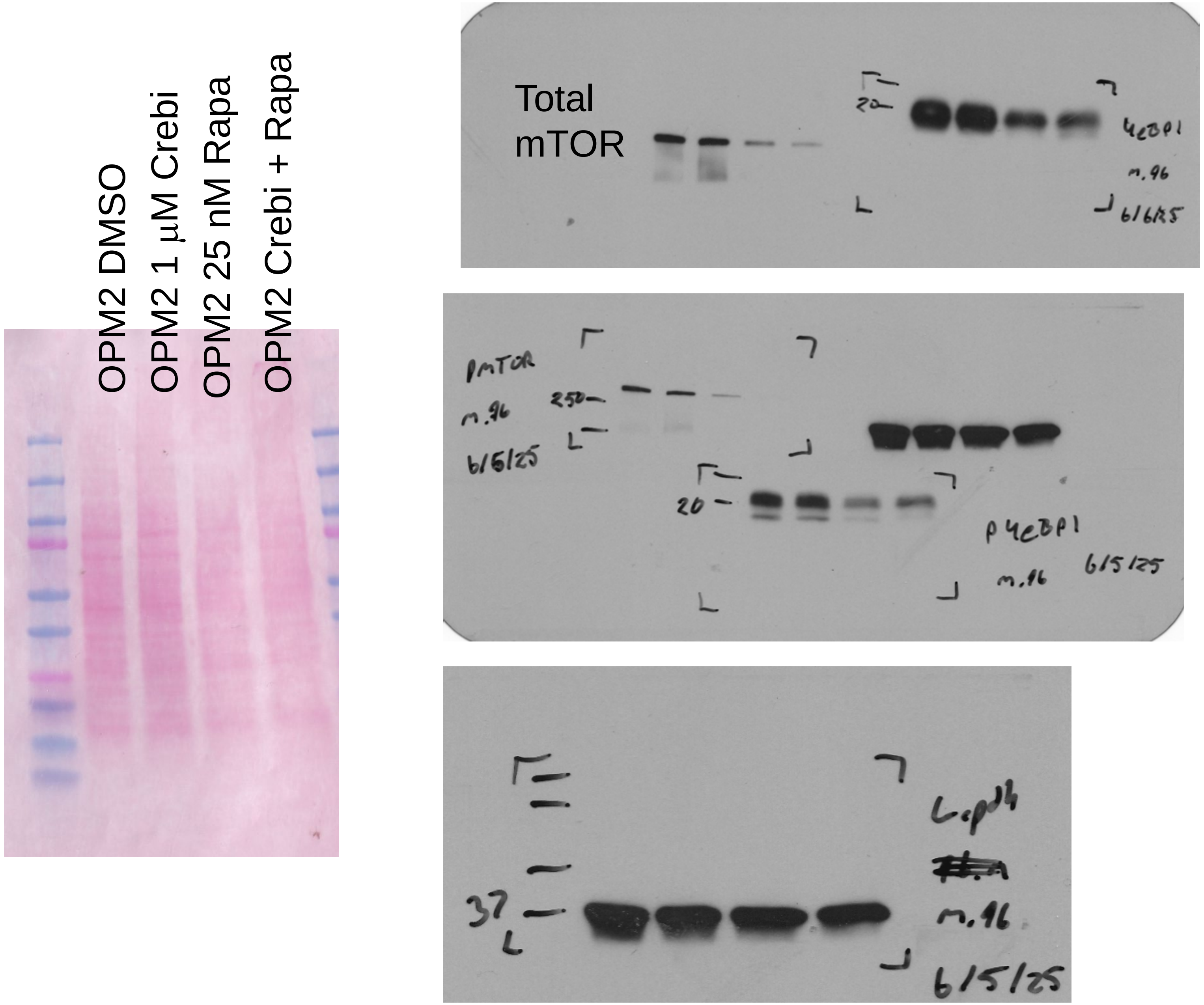
OPM2 DMSO
OPM2 1 μM Crebi
OPM2 25 nM Rapa
OPM2 Crebi + Rapa
Total mTOR
20
4EBP1
m.96
pmTOR
250
6/5/25
20
p4EBP1
Gapdh
37

Figure S4H

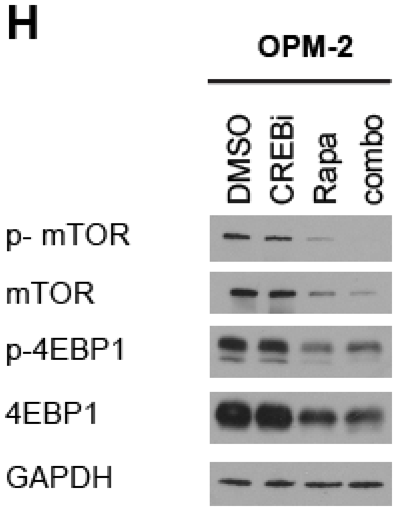
H
OPM-2
DMSO
CREBi
Rapa
combo
p- mTOR
mTOR
p-4EBP1
4EBP1
GAPDH

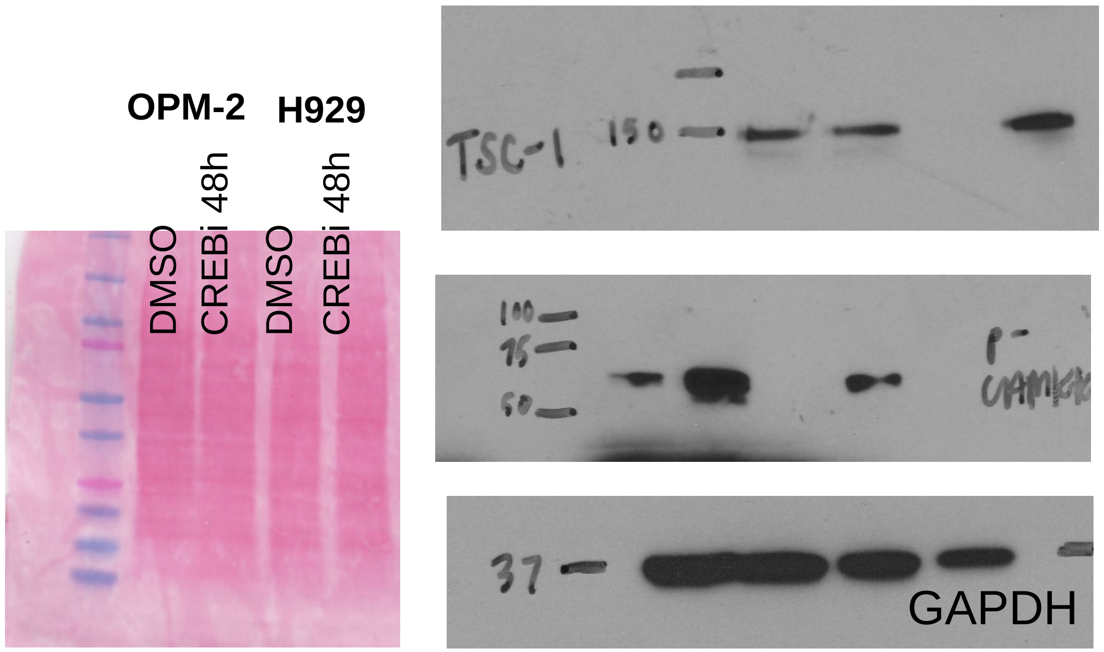
OPM-2
H929
DMSO
CREBi 48h
DMSO
CREBi 48h
TSC-1
150
100
75
50
p-CAMKK
37
GAPDH

Figure S4J

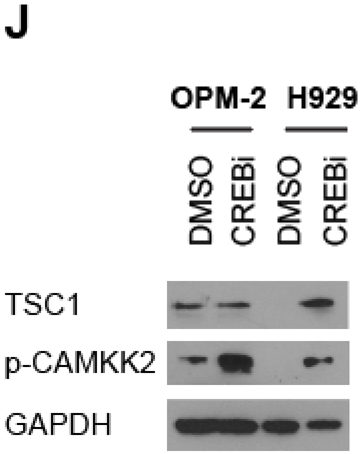
J
OPM-2
H929
DMSO
CREBi
DMSO
CREBi
TSC1
p-CAMKK2
GAPDH

Figure S5C

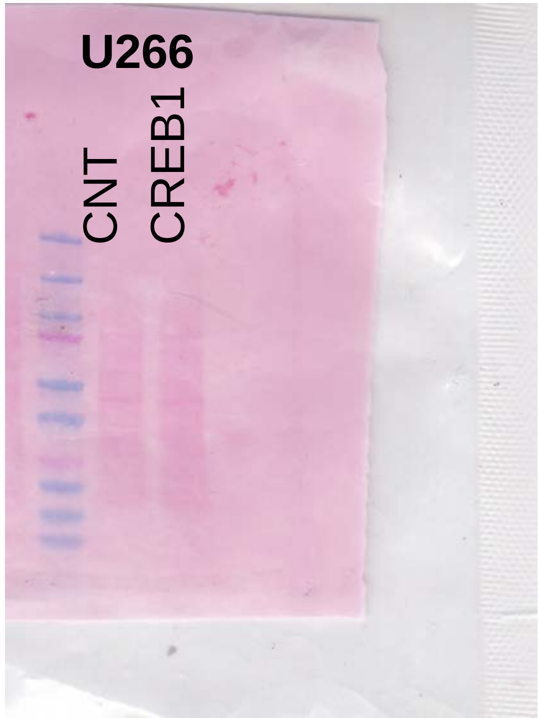
U266
CNT
CREB1

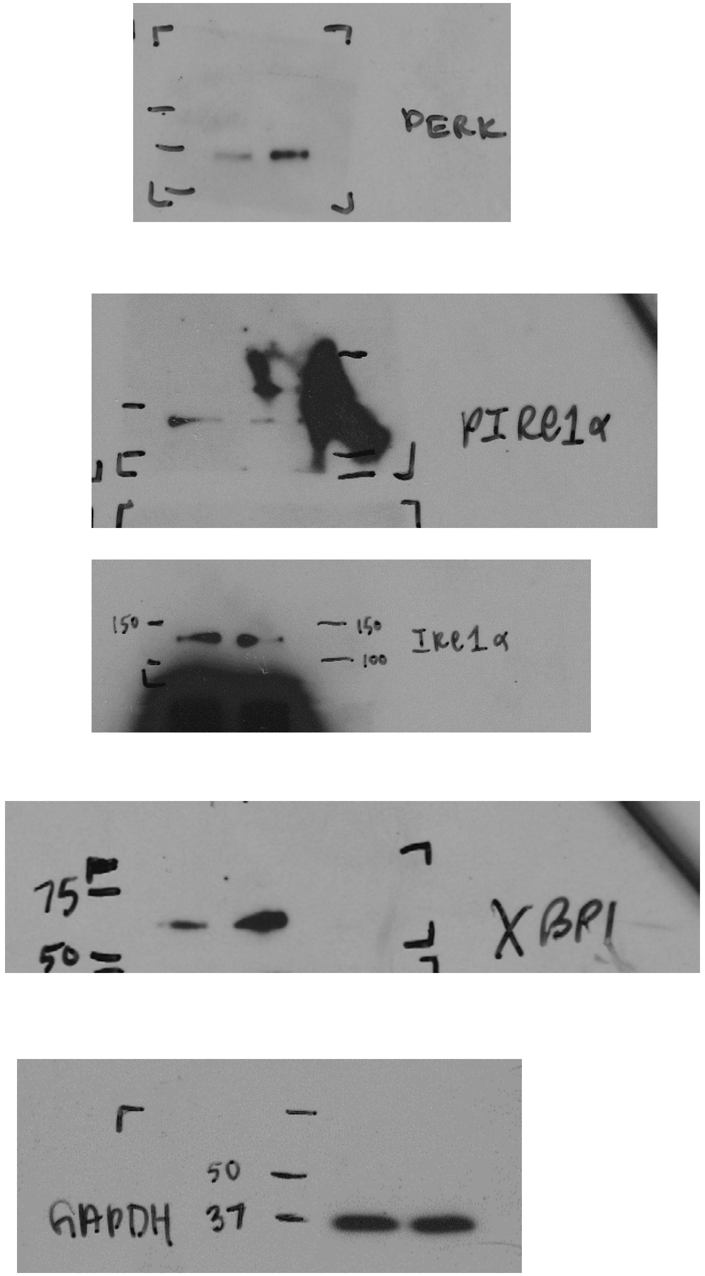
PERK
pIRe1α
150
150
100
IRe1α
75
50
XBPl
GAPDH
50
37

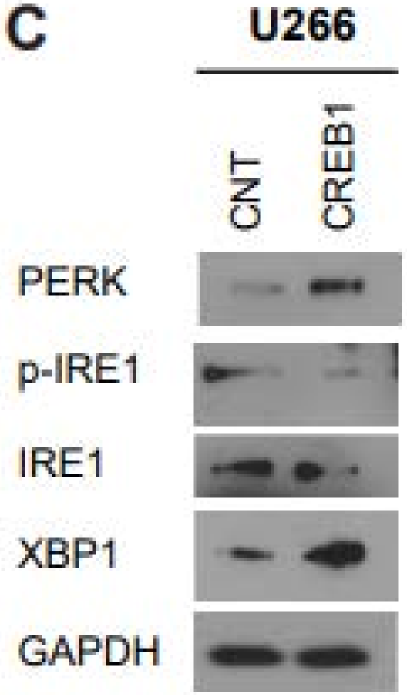
C
U266
CNT
CREB1
PERK
p-IRE1
IRE1
XBP1
GAPDH

Figure S5D

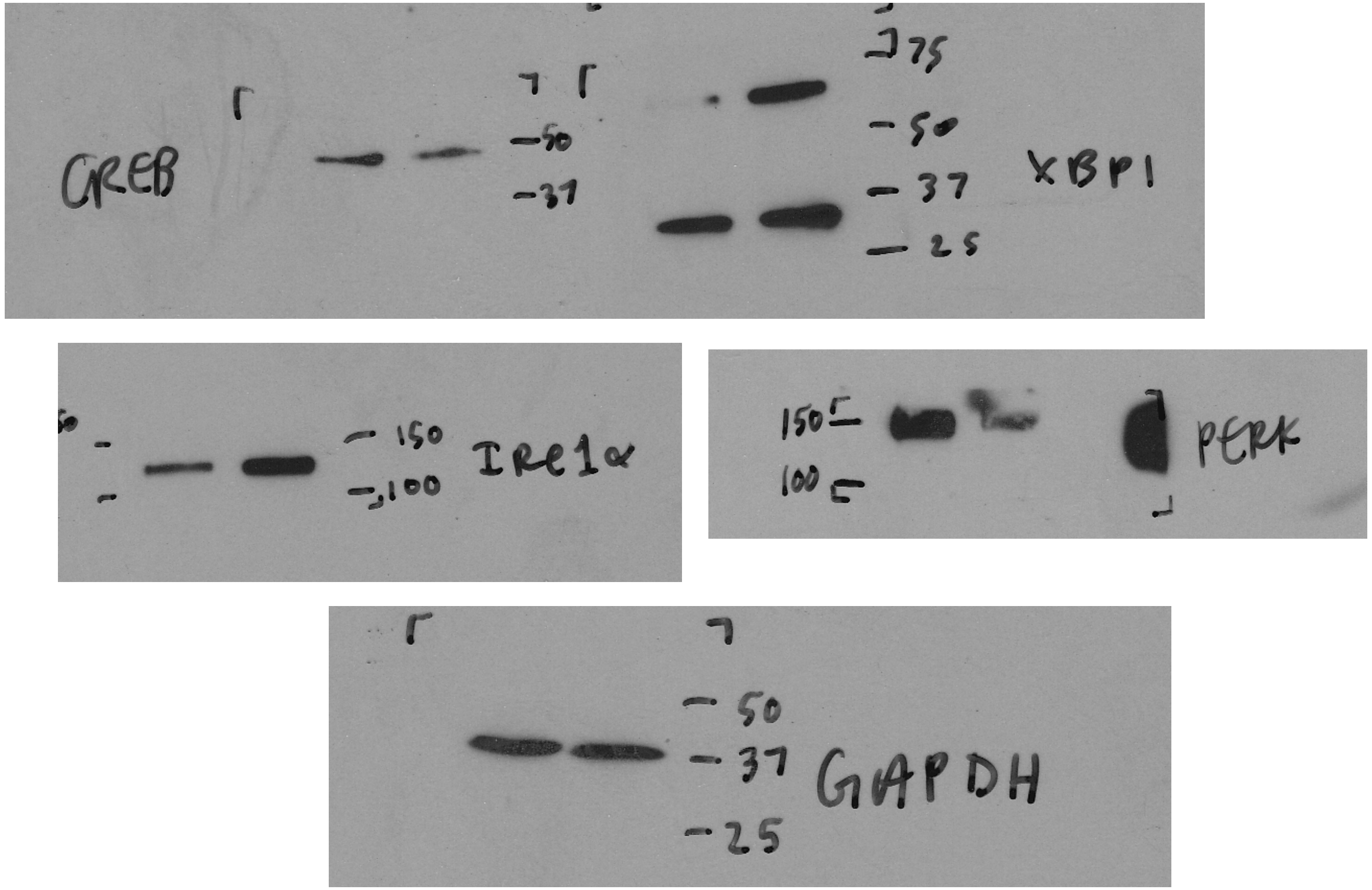
CREB
-50
-37
75
-50
-37
-25
XBP1
-150
-100
IRE1α
150
100
PERK
-50
-37
-25
GAPDH

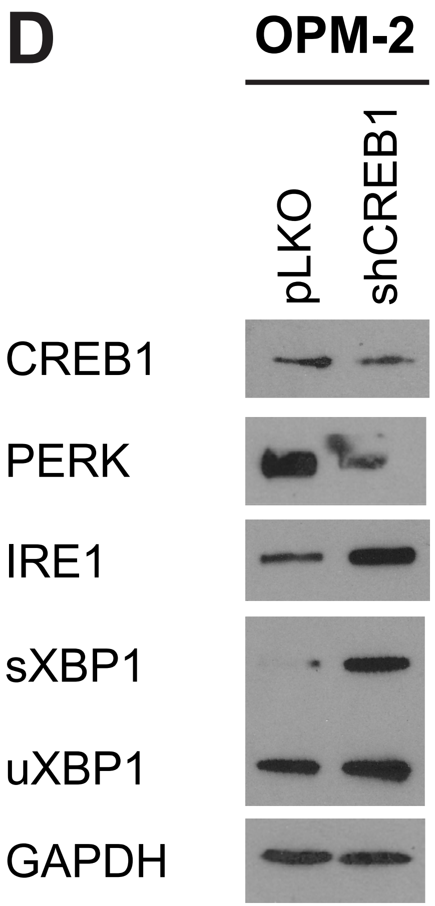
D
OPM-2
pLKO
shCREB1
CREB1
PERK
IRE1
sXBP1
uXBP1
GAPDH

Figure 3G **Figure 3H**

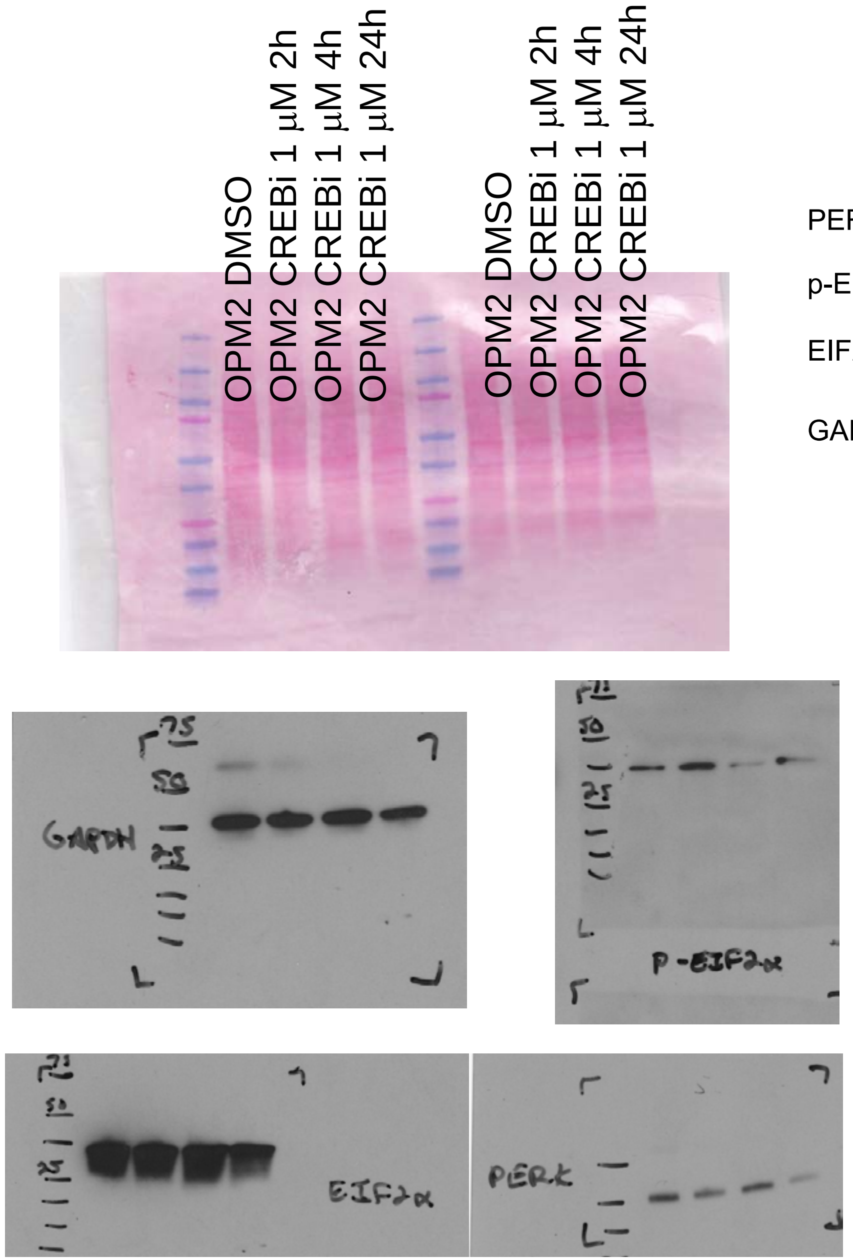

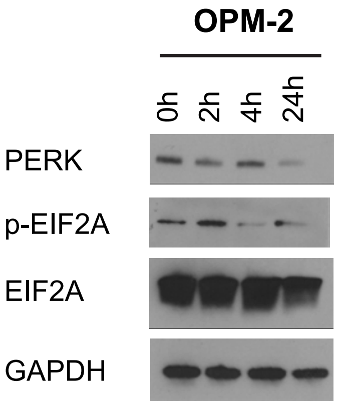

Figure 3H

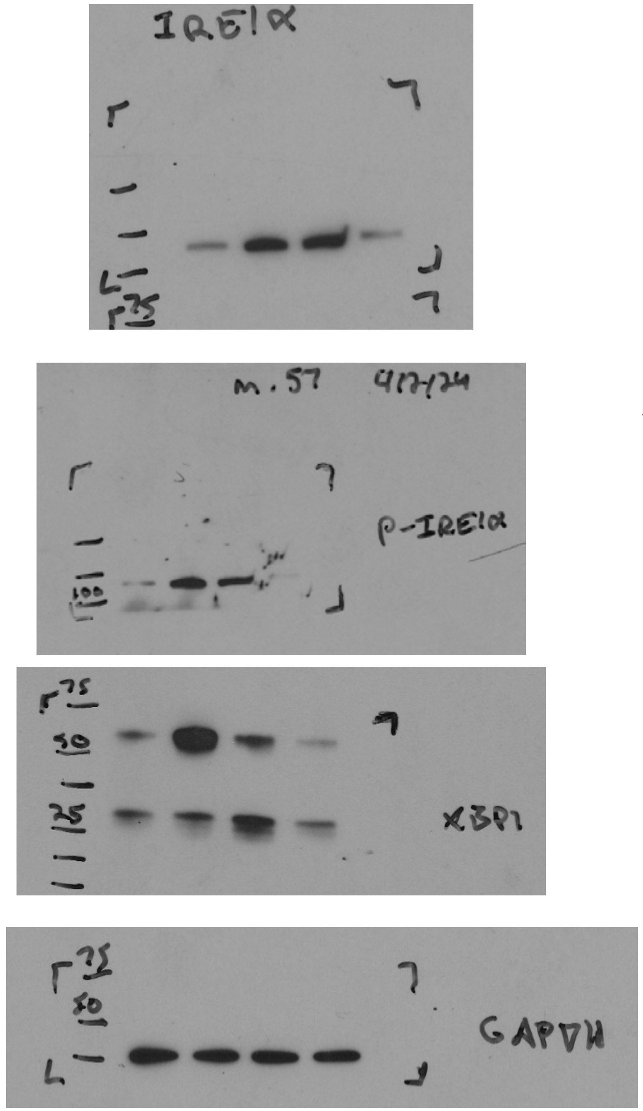

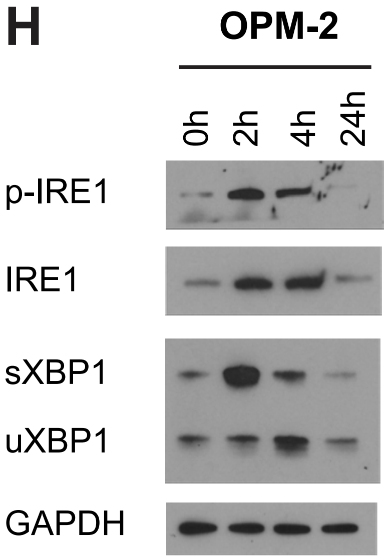

Figure 3I

OPM-2

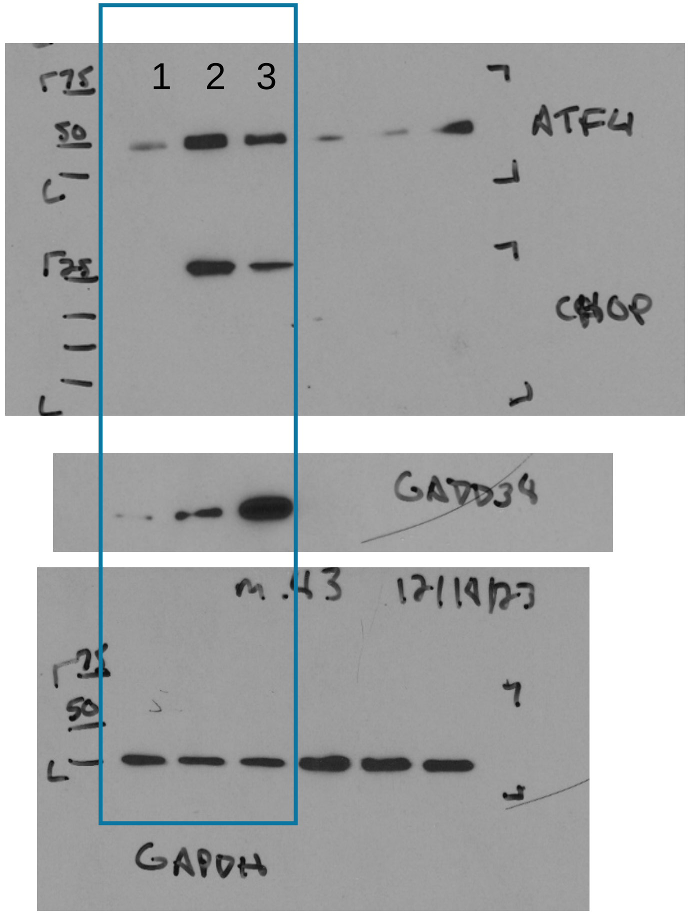

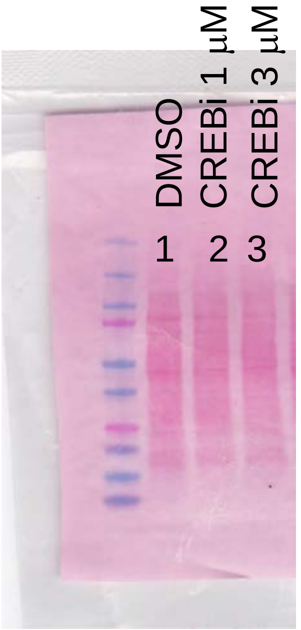

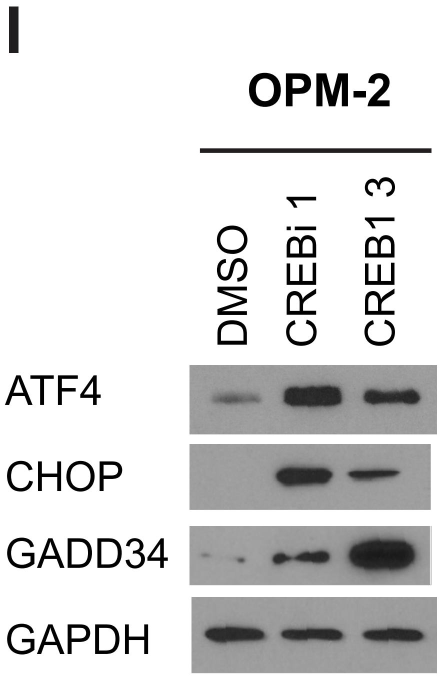

Figure 3K

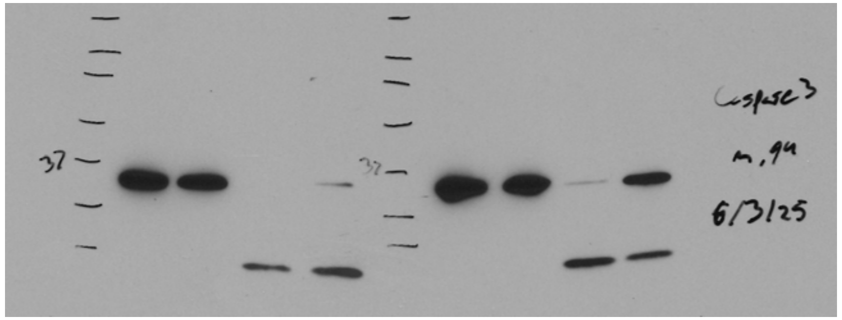
Caspase3
m.94
6/3/25
37

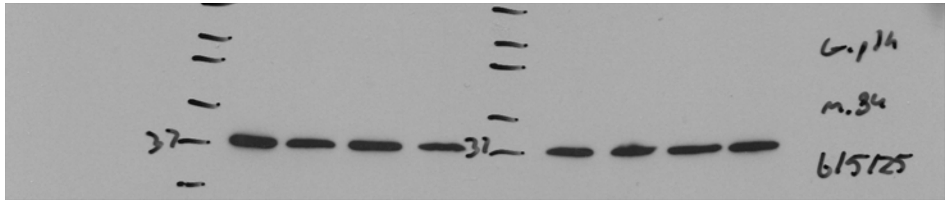
Gapdh
m.84
6/5/25
37

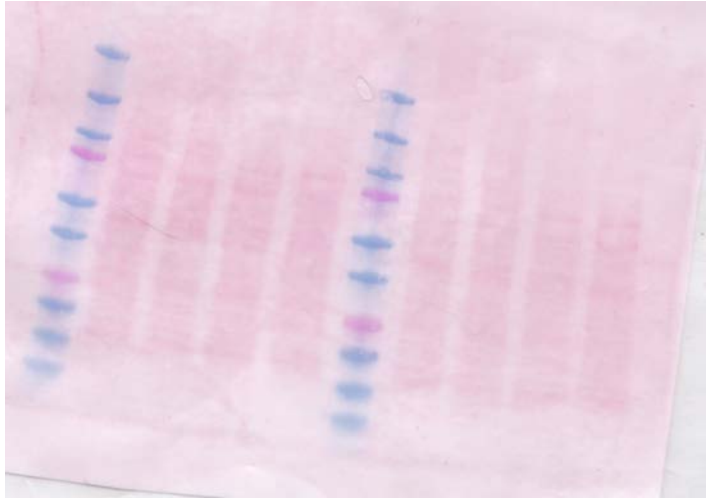

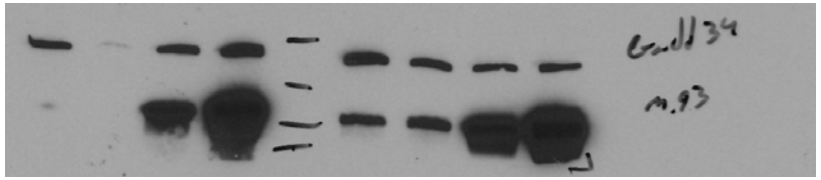
Gadd34
m.93

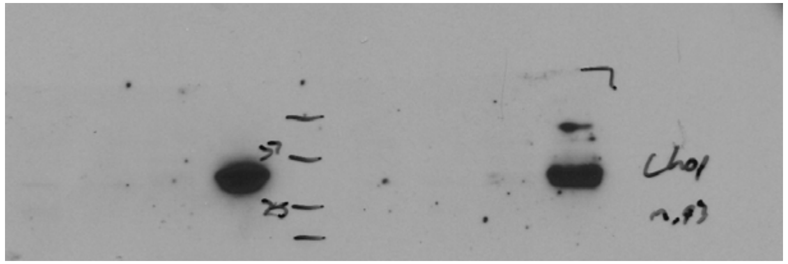
Chop
m.93
37
25

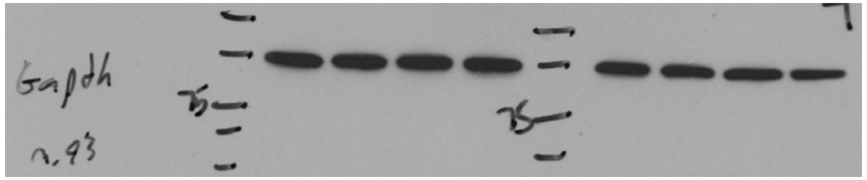
Gapdh
m.93
75

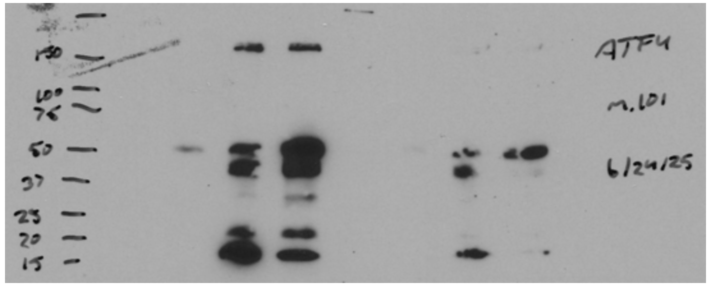
ATF4
m.101
6/24/25
150
100
75
50
37
25
20
15

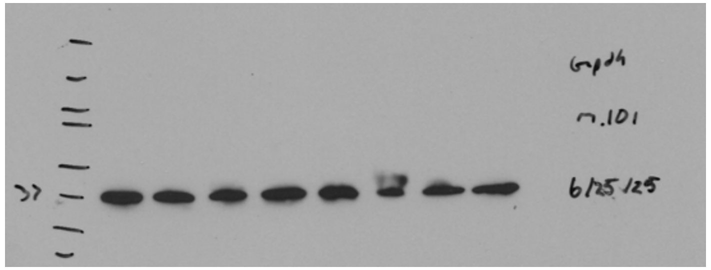
Gapdh
m.101
6/25/25
37

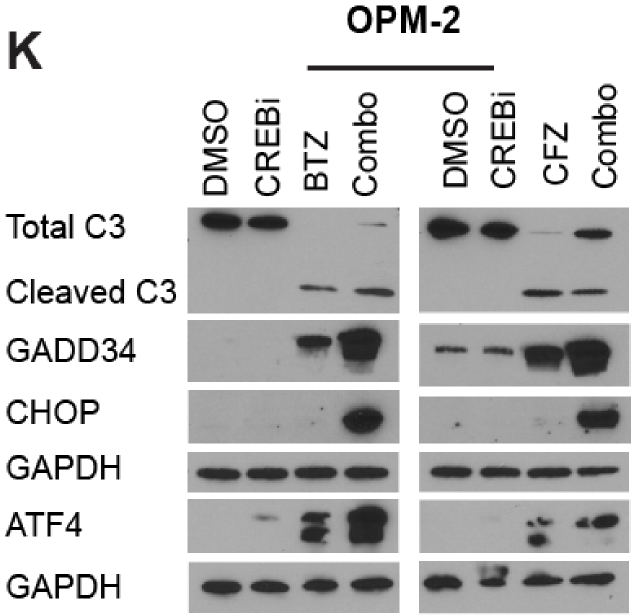
K
OPM-2
DMSO
CREBi
BTZ
Combo
DMSO
CREBi
CFZ
Combo
Total C3
Cleaved C3
GADD34
CHOP
GAPDH
ATF4
GAPDH

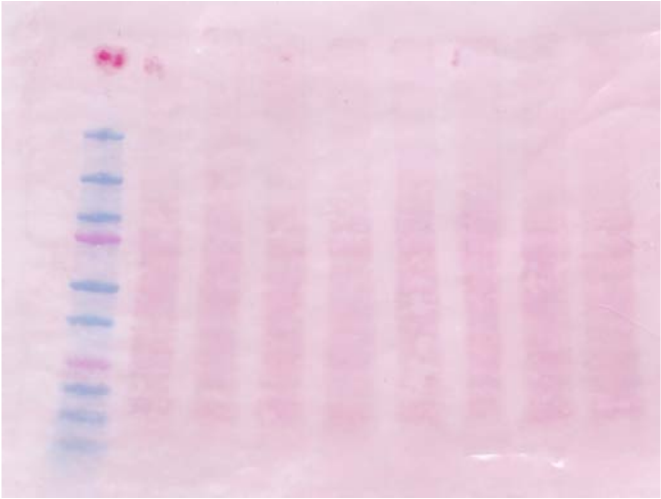

Figure S7C

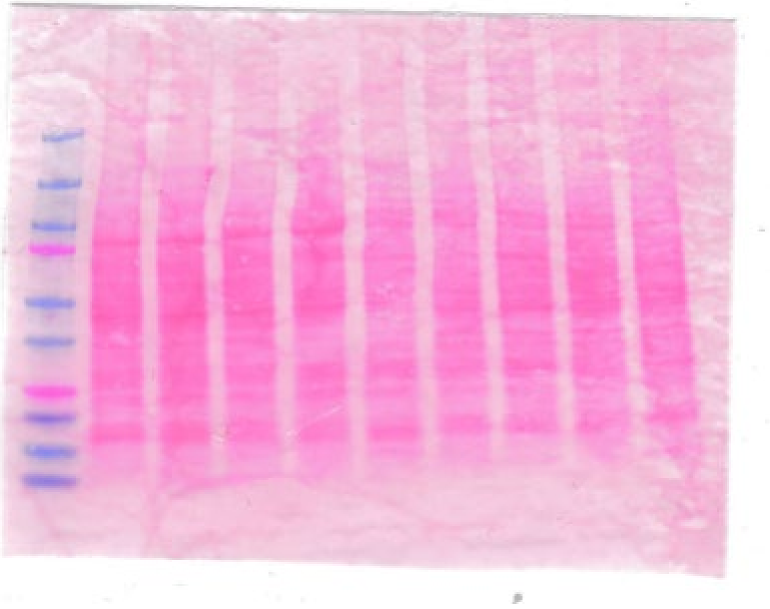

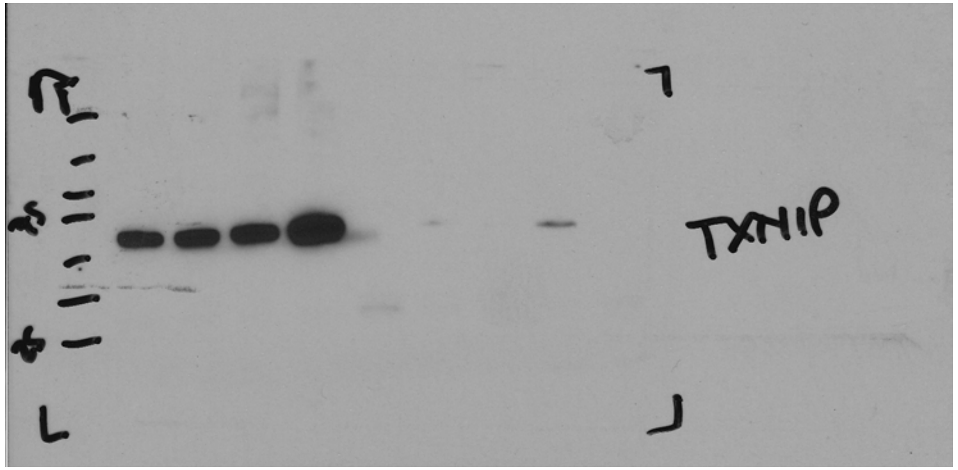
TXNIP

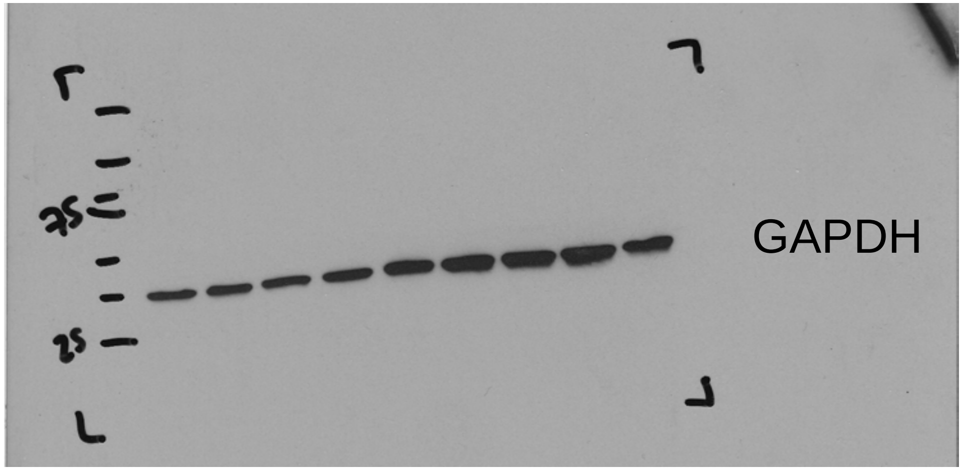
GAPDH

C

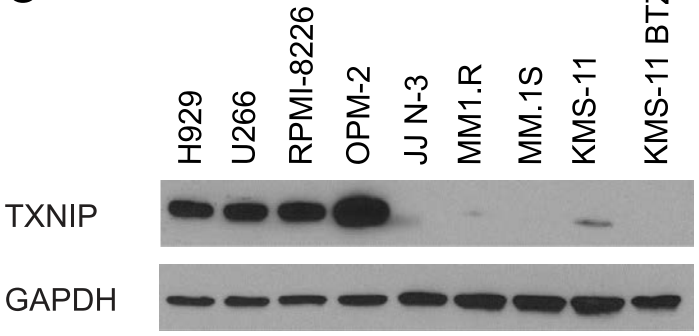
H929
U266
RPMI-8226
OPM-2
JJ N-3
MM1.R
MM.1S
KMS-11
KMS-11 BTZ
TXNIP
GAPDH

Figure S7D

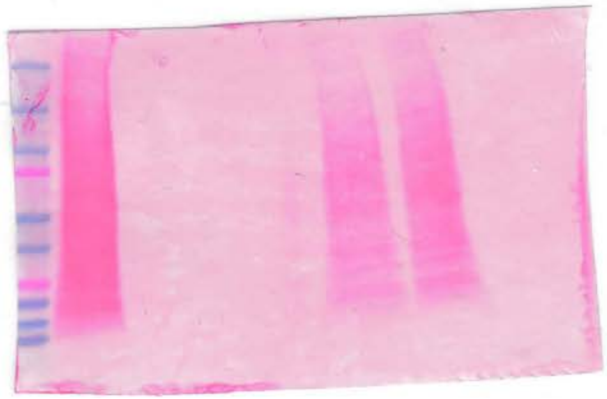

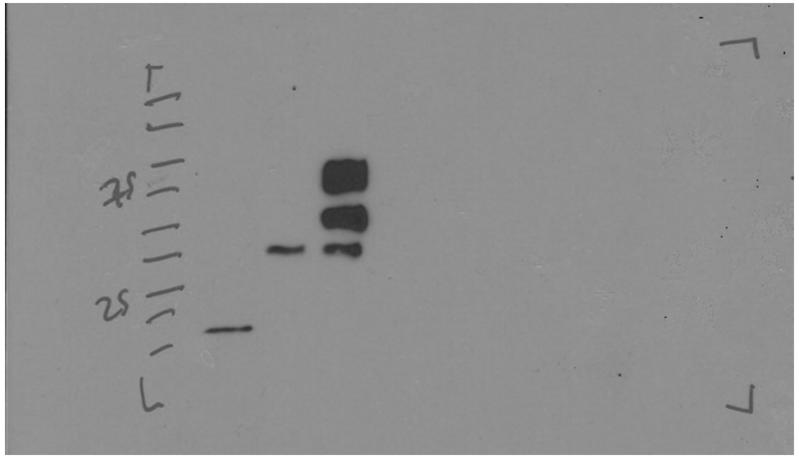

75
25

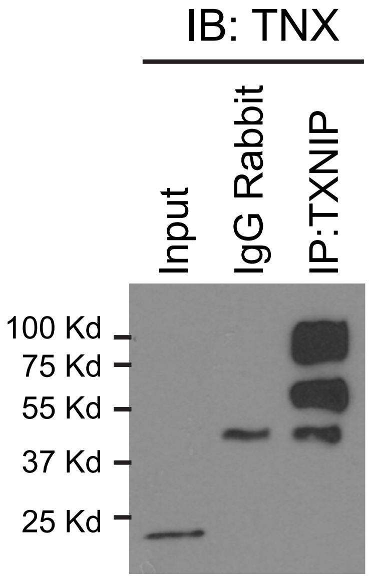

IB: TNX
Input
IgG Rabbit
IP:TXNIP
100 Kd
75 Kd
55 Kd
37 Kd
25 Kd

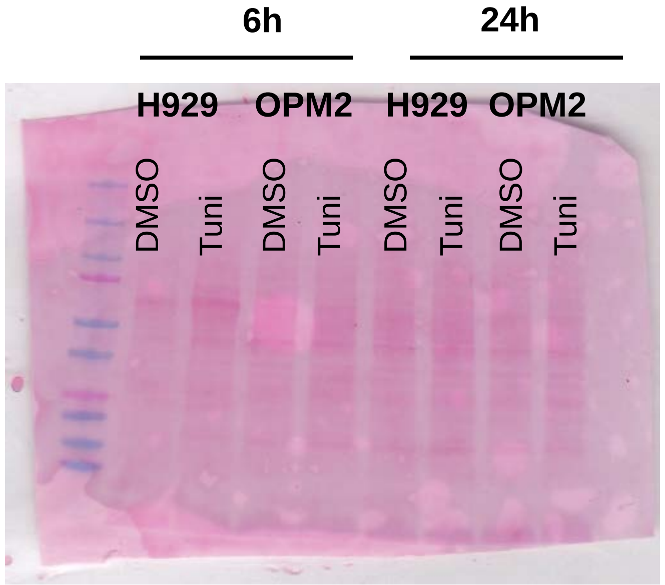

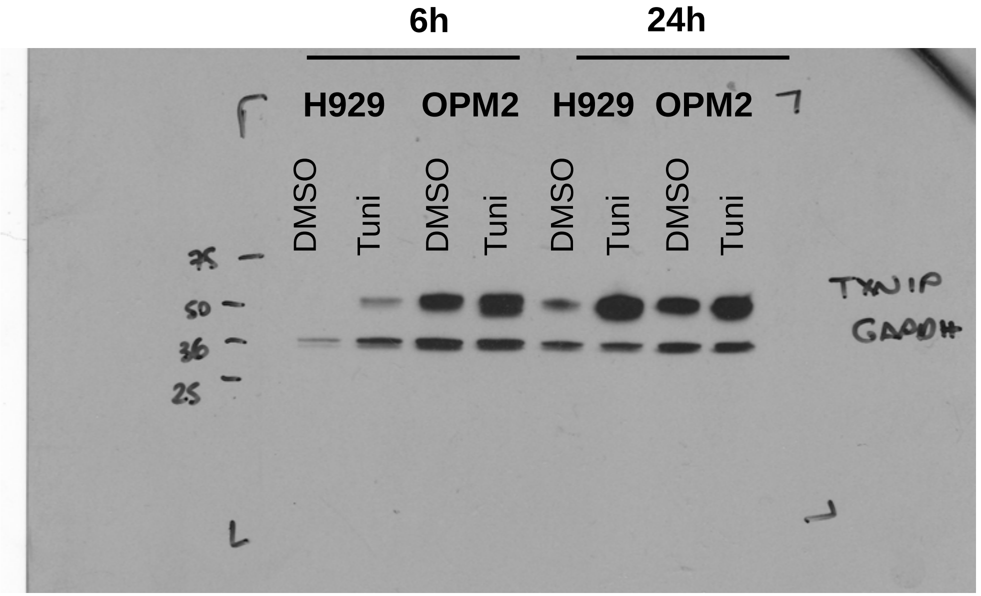

Figure S7E

Only 24h treatment is shown in the figure

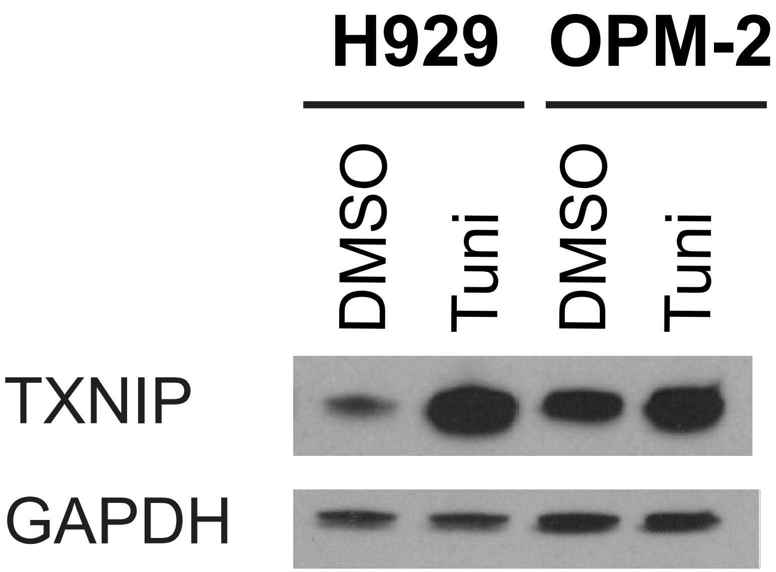

Figure 4I

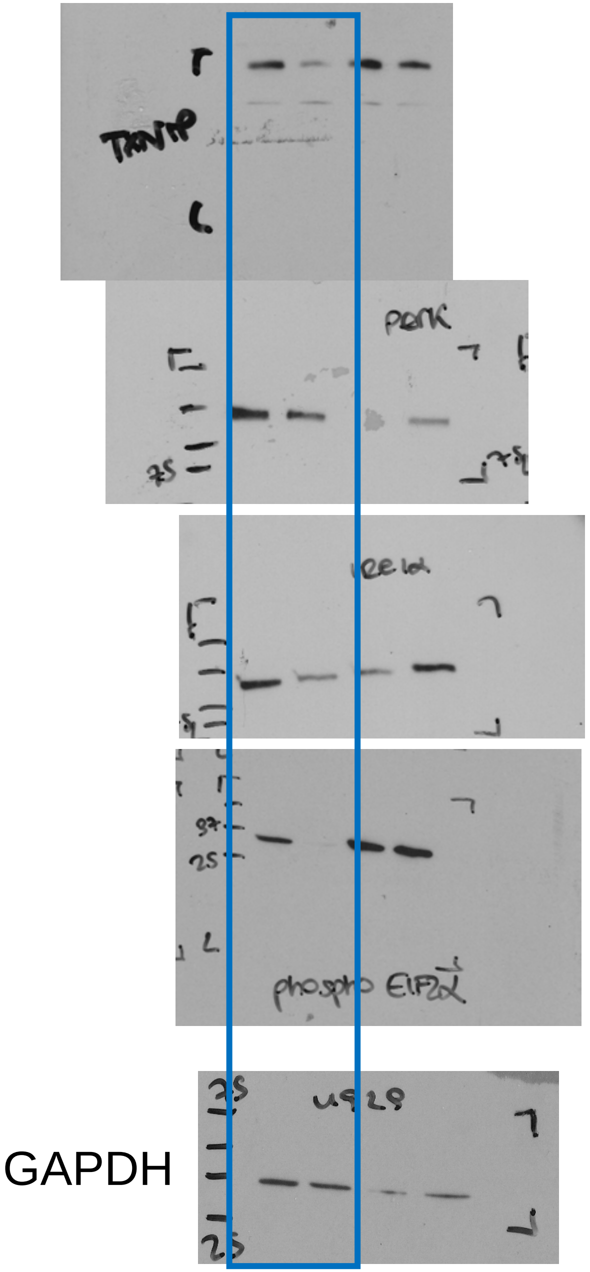

GAPDH

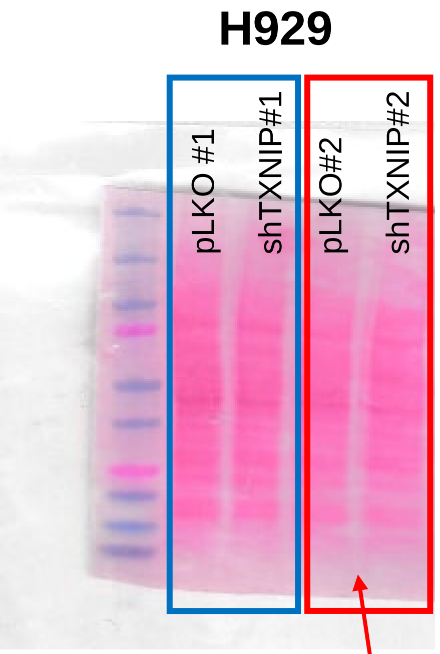

In red silencing for TXNIP did not work and therefore data were not used

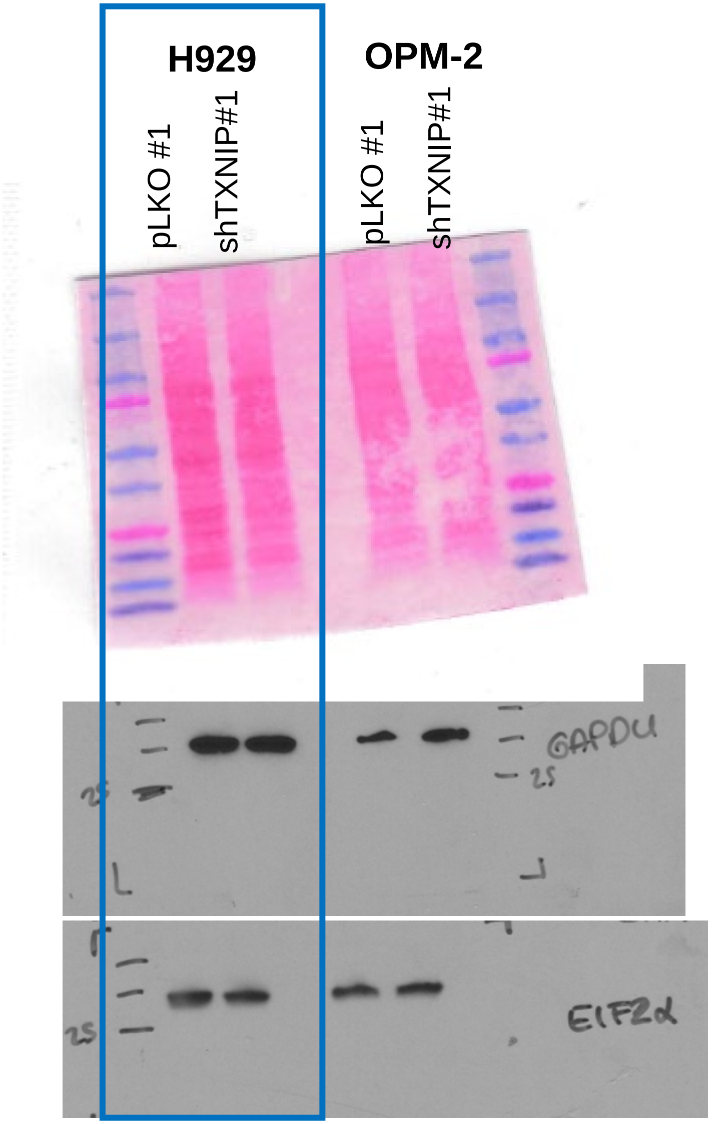

I

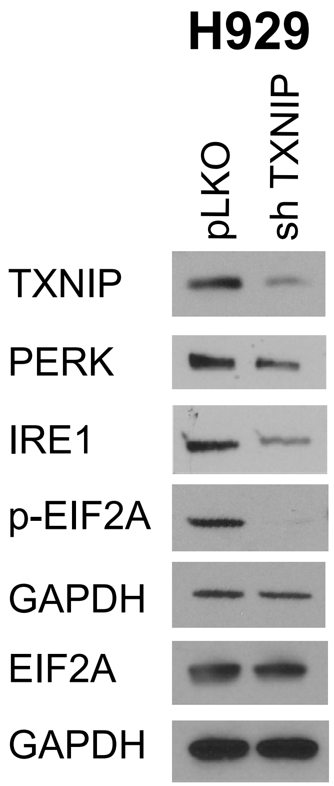

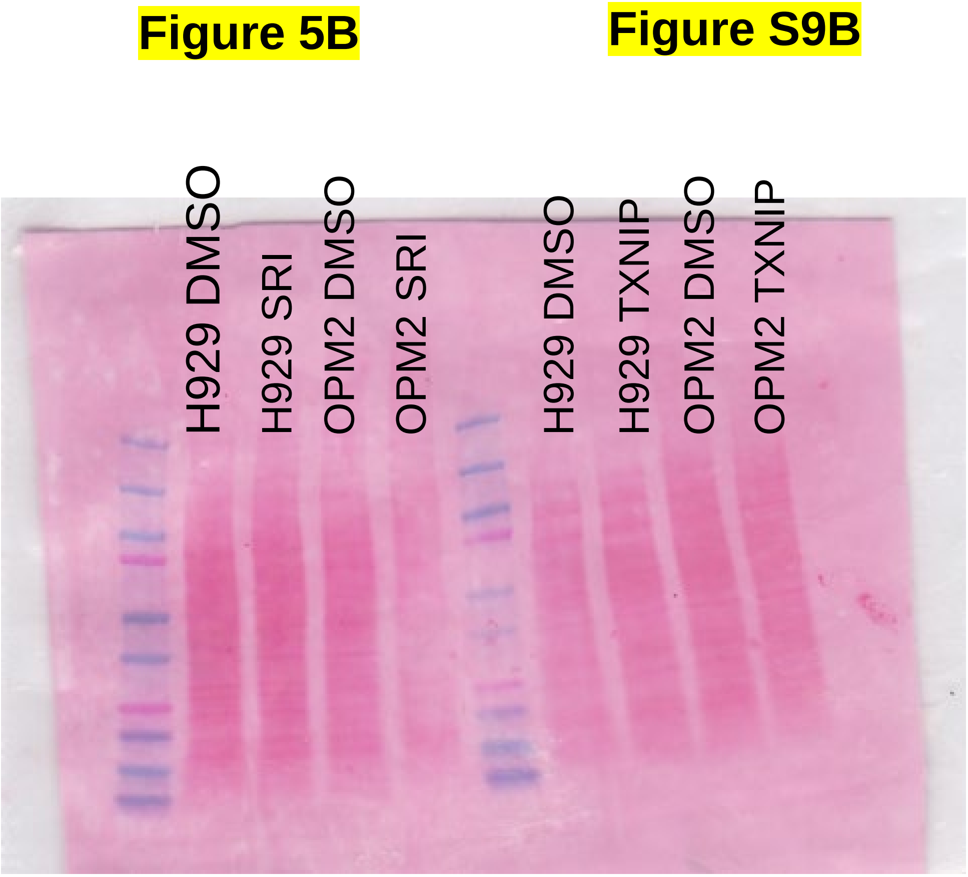
Figure 5B
Figure S9B
H929 DMSO
H929 SRI
OPM2 DMSO
OPM2 SRI
H929 DMSO
H929 TXNIP
OPM2 DMSO
OPM2 TXNIP

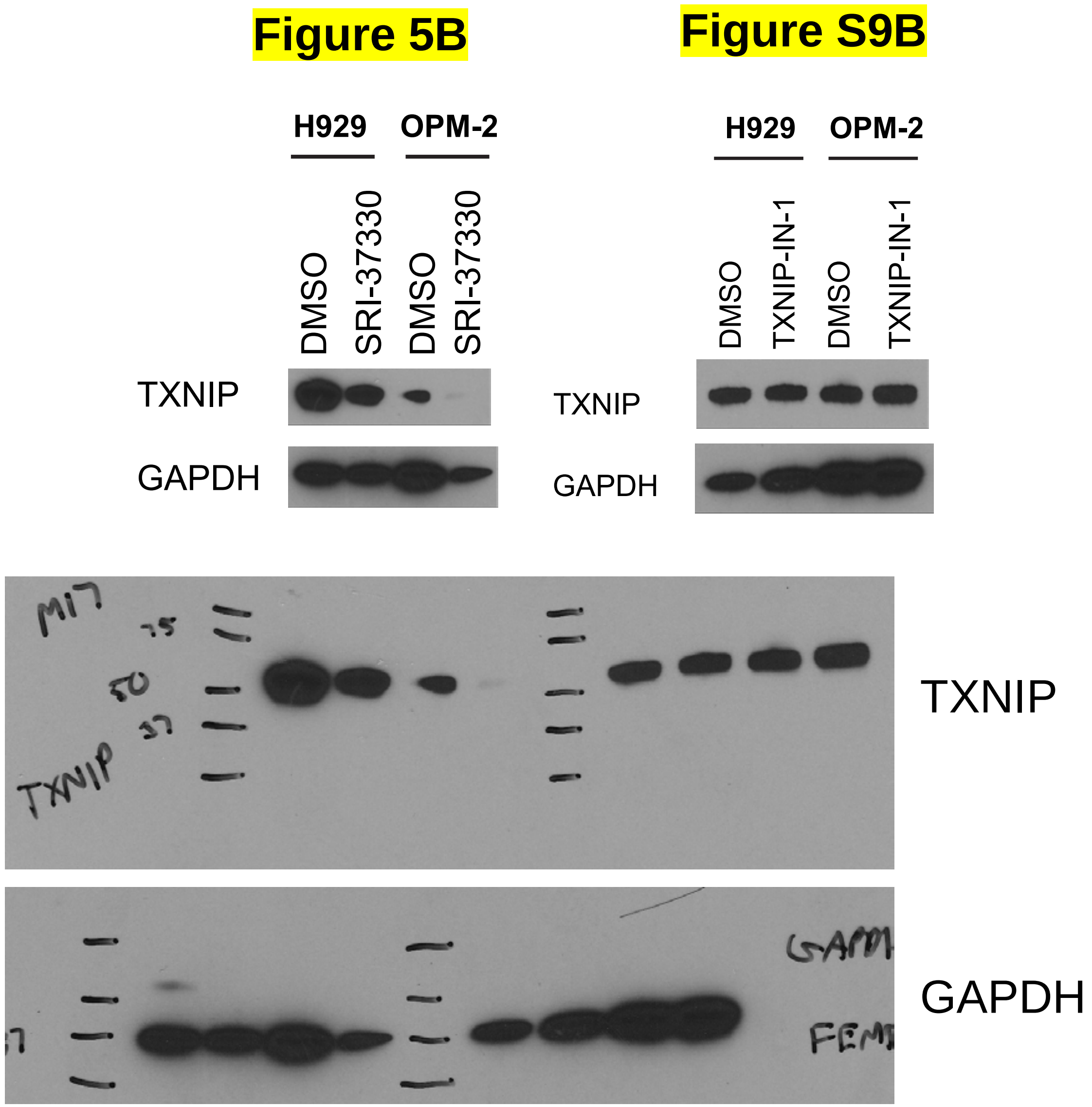
Figure 5B
Figure S9B
H929
OPM-2
DMSO
SRI-37330
DMSO
SRI-37330
H929
OPM-2
DMSO
TXNIP-IN-1
DMSO
TXNIP-IN-1
TXNIP
GAPDH
TXNIP
GAPDH
TXNIP
GAPDH

Figure 5G

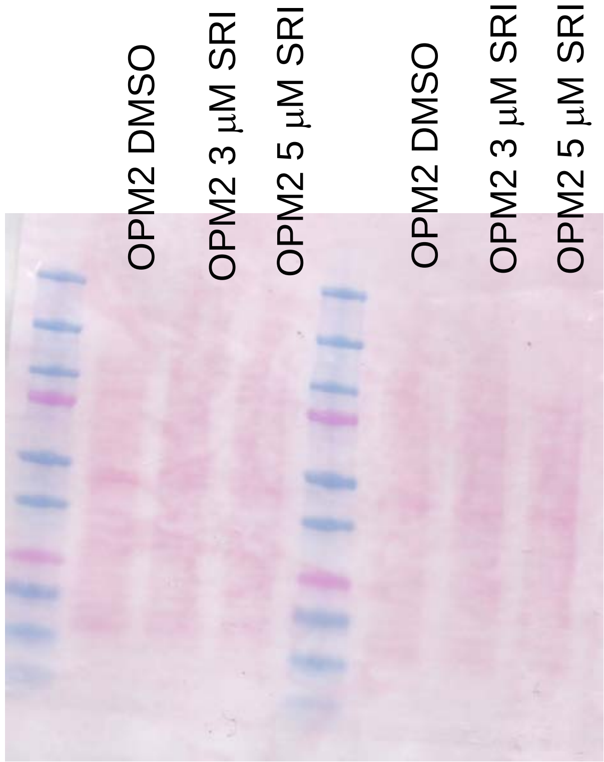
OPM2 DMSO
OPM2 3 µM SRI
OPM2 5 µM SRI
OPM2 DMSO
OPM2 3 µM SRI
OPM2 5 µM SRI

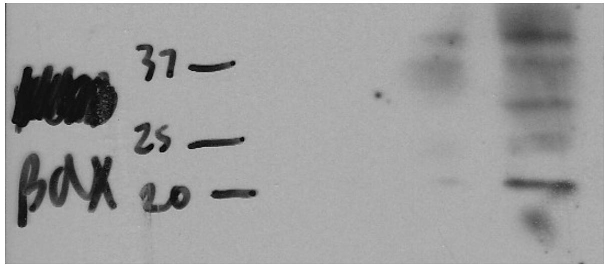
37
25
20
Bax

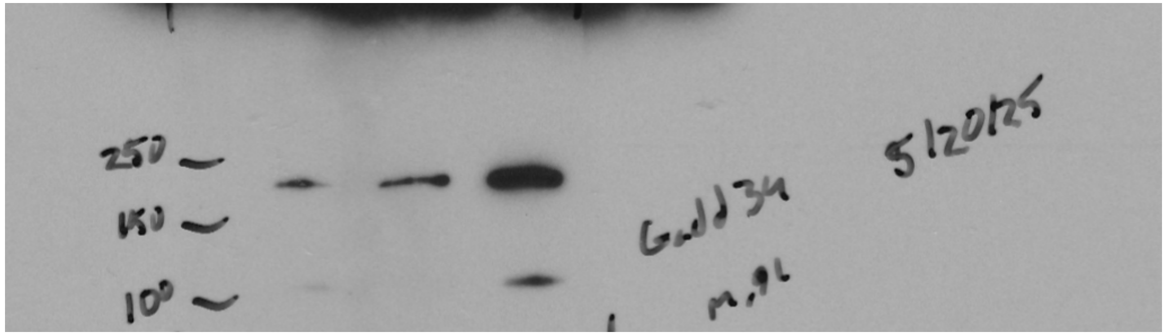
250
150
100
Gadd34
5/20/25

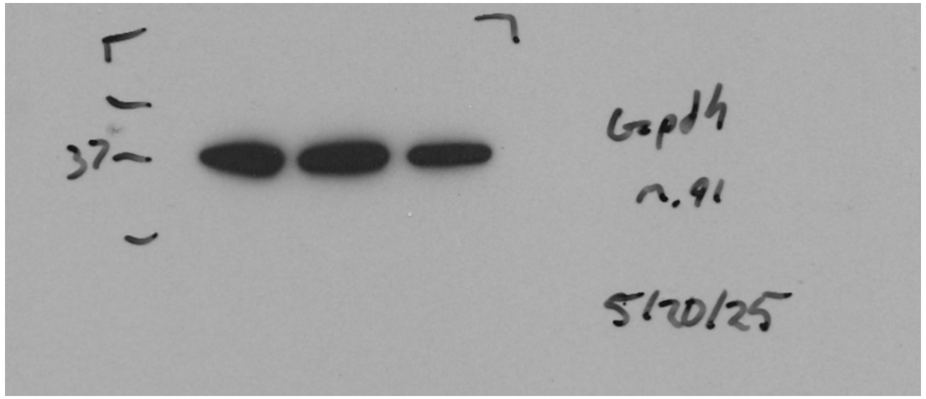
37
Gapdh
5/20/25

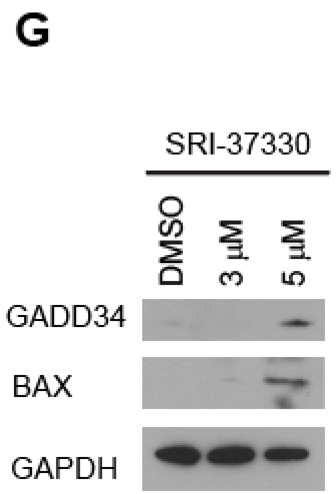
G
SRI-37330
DMSO
3 µM
5 µM
GADD34
BAX
GAPDH

Figure 5J

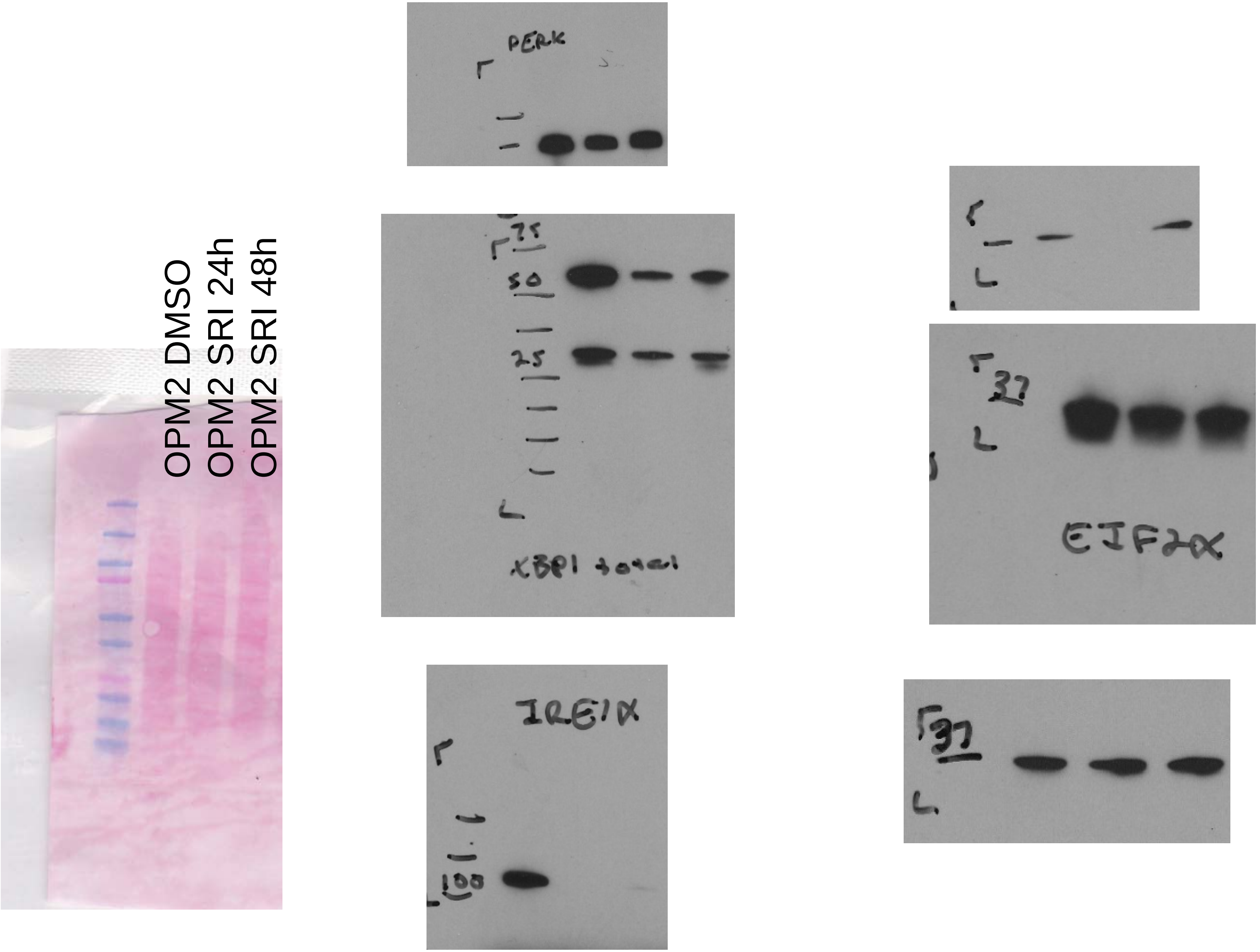

OPM2 DMSO
OPM2 SRI 24h
OPM2 SRI 48h
PERK
75
50
25
XBP1 total
IRE1α
100
37
EIF2α
37

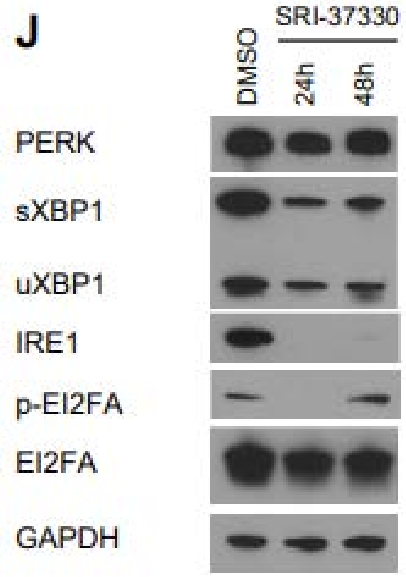

J
SRI-37330
DMSO
24h
48h
PERK
sXBP1
uXBP1
IRE1
p-EI2FA
EI2FA
GAPDH

Figure S10C

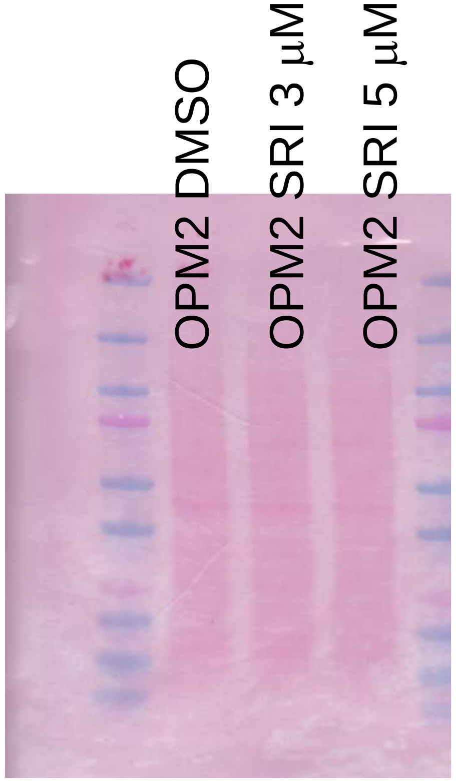
OPM2 DMSO
OPM2 SRI 3 µM
OPM2 SRI 5 µM

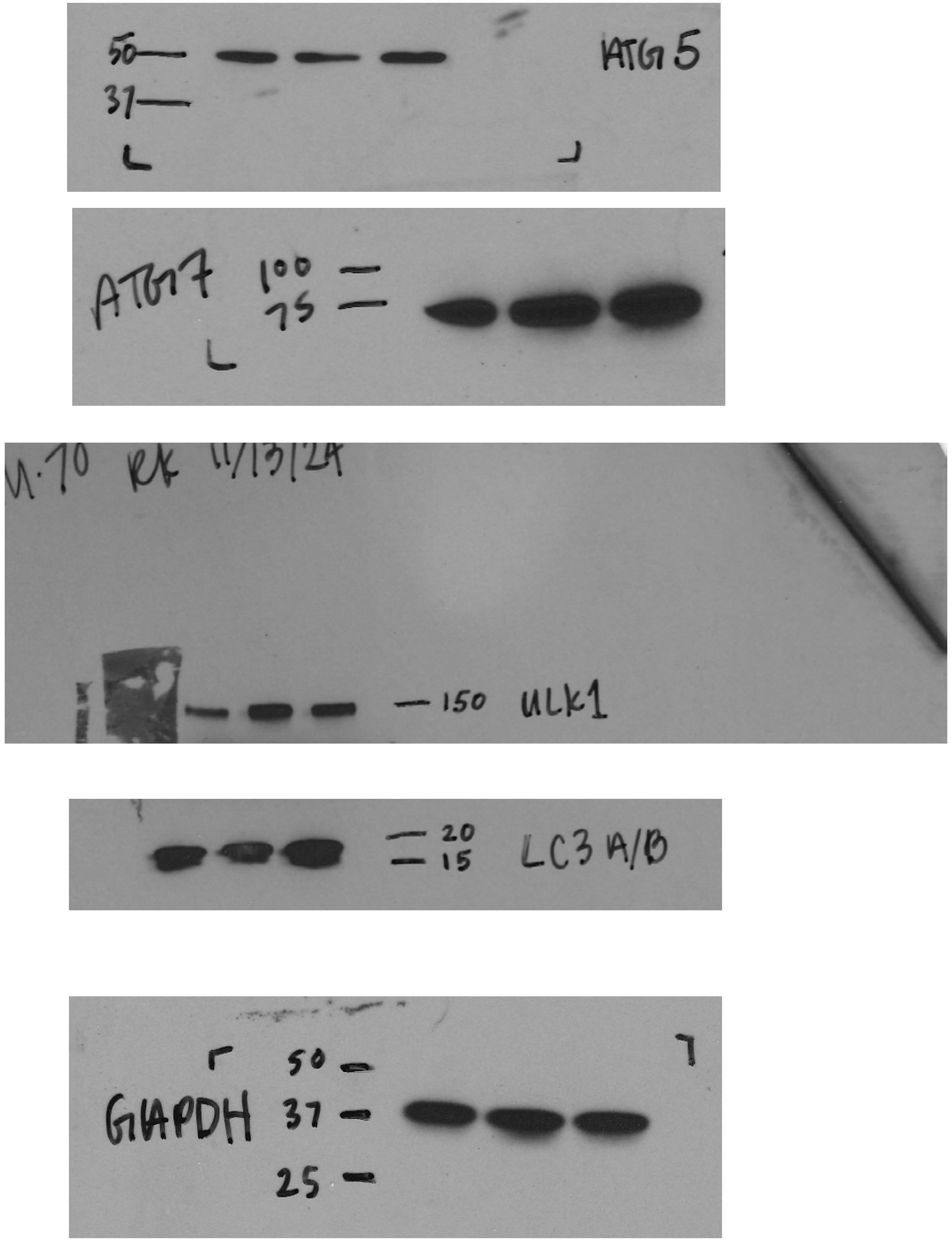
ATG5
50
37
ATG7
100
75
150
ULK1
20
15
LC3 A/B
GAPDH
50
37
25

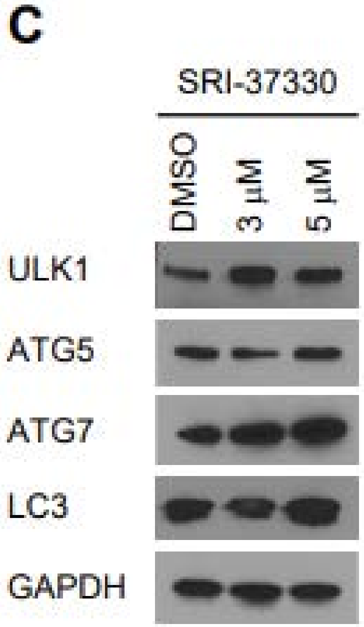
C
SRI-37330
DMSO
3 µM
5 µM
ULK1
ATG5
ATG7
LC3
GAPDH

Figure S10E

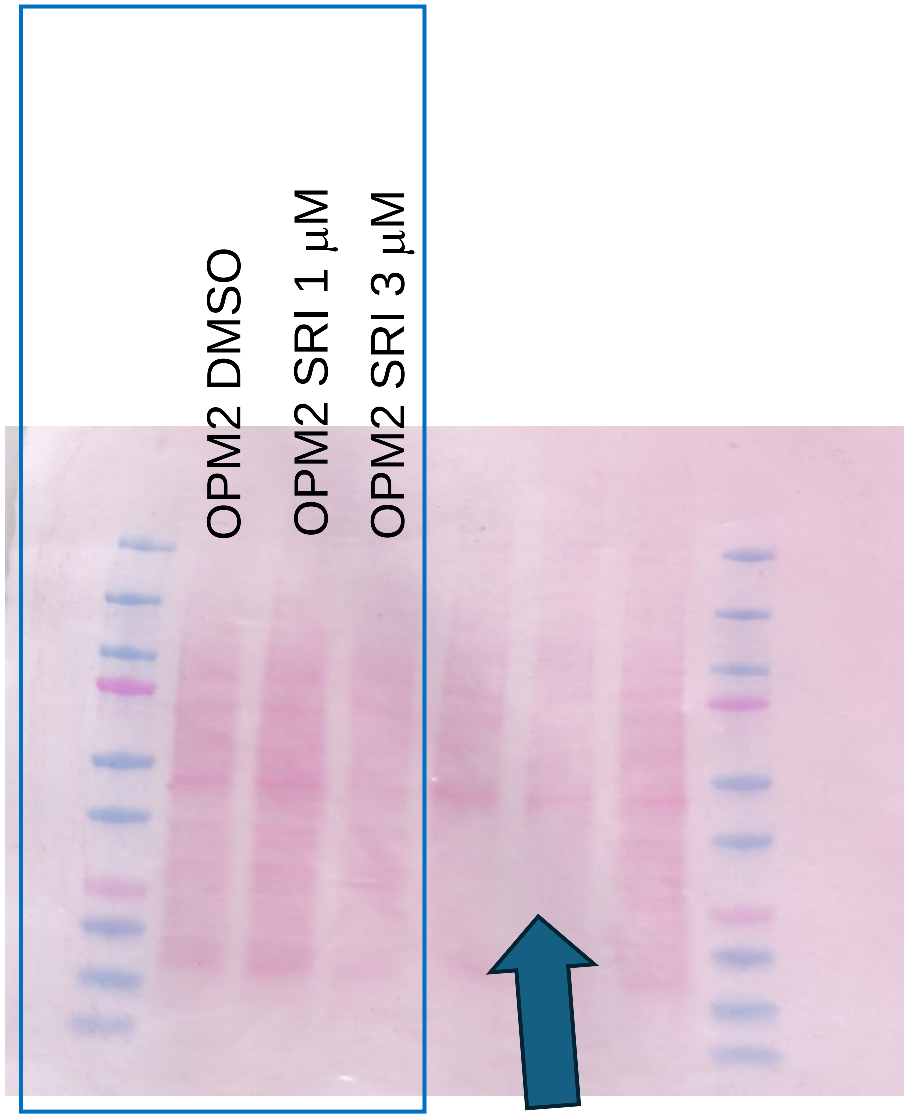

Issue with Ponceau-Not used in the paper

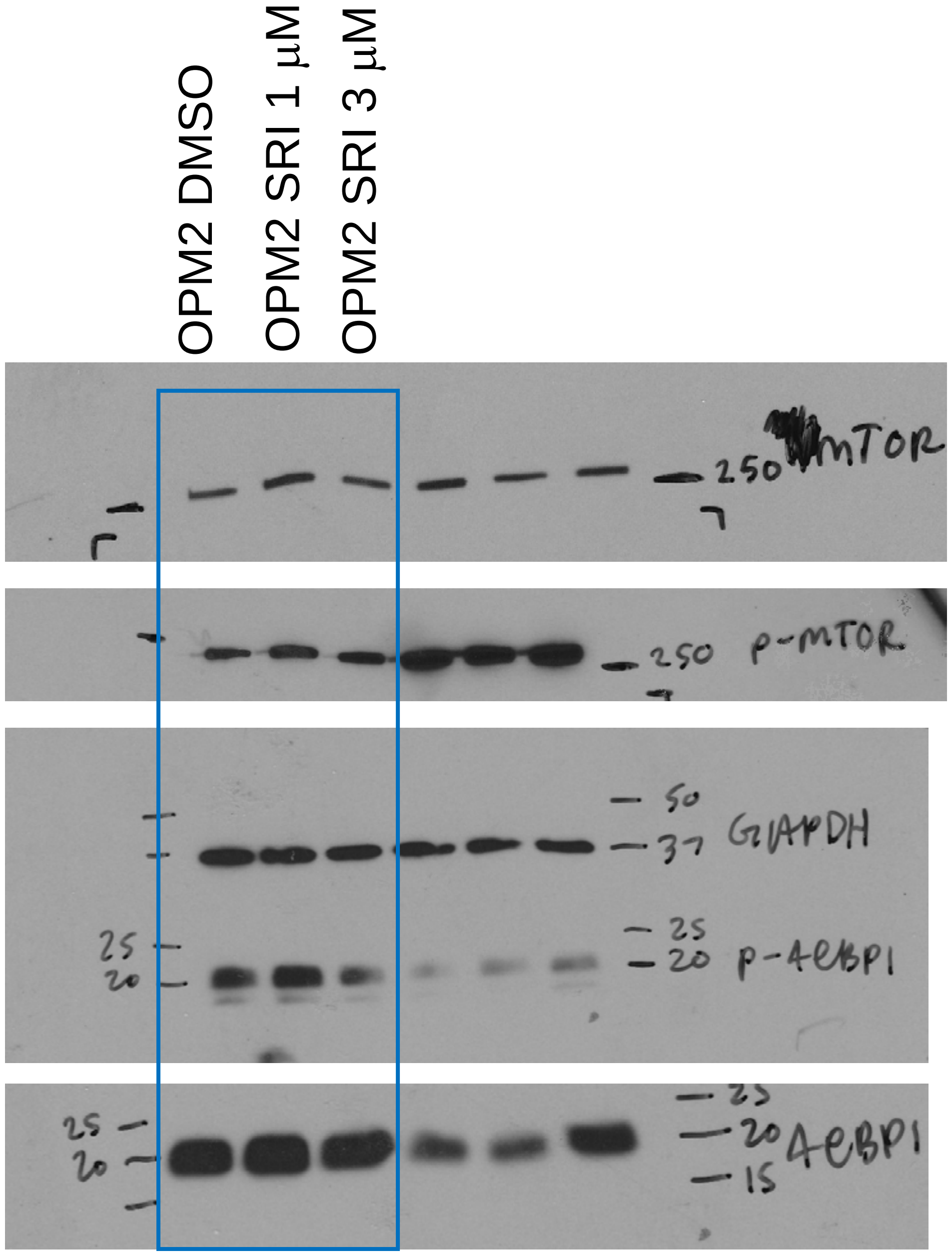

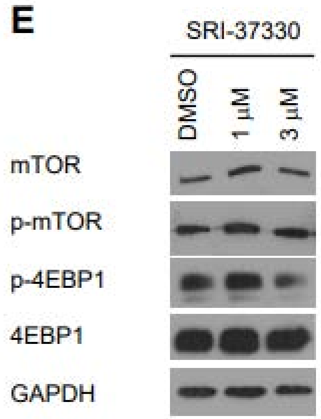

Supplement: Supplementary file 2 — Western blot original films [file 41419_2025_8246_MOESM2_ESM.pdf]
